# Mcadet: A feature selection method for fine-resolution single-cell RNA-seq data based on multiple correspondence analysis and community detection

**DOI:** 10.1371/journal.pcbi.1012560

**Published:** 2024-10-28

**Authors:** Saishi Cui, Sina Nassiri, Issa Zakeri

**Affiliations:** 1 Department of Epidemiology and Biostatistics, Dornsife School of Public Health, Drexel University, Philadelphia, Pennsylvania, United States of America; 2 Roche Pharma Research and Early Development, Roche Innovation Center Basel, Basel, Switzerland; Max-Delbruck-Centrum fur Molekulare Medizin in der Helmholtz-Gemeinschaft, GERMANY

## Abstract

Single-cell RNA sequencing (scRNA-seq) data analysis faces numerous challenges, including high sparsity, a high-dimensional feature space, and biological noise. These challenges hinder downstream analysis, necessitating the use of feature selection methods to identify informative genes, and reduce data dimensionality. However, existing methods for selecting highly variable genes (HVGs) exhibit limited overlap and inconsistent clustering performance across benchmark datasets. Moreover, these methods often struggle to accurately select HVGs from fine-resolution scRNA-seq datasets and minority cell types, which are more difficult to distinguish, raising concerns about the reliability of their results. To overcome these limitations, we propose a novel feature selection framework for scRNA-seq data called Mcadet. Mcadet integrates Multiple Correspondence Analysis (MCA), graph-based community detection, and a novel statistical testing approach. To assess the effectiveness of Mcadet, we conducted extensive evaluations using both simulated and real-world data, employing unbiased metrics for comparison. Our results demonstrate the superior performance of Mcadet in the selection of HVGs in scenarios involving fine-resolution scRNA-seq datasets and datasets containing minority cell populations. Overall, we demonstrate that Mcadet enhances the reliability of selected HVGs, although the impact of HVG selection on various downstream analyses varies and needs to be further investigated.

## Author summary

scRNA-seq brings both great opportunities and challenges for transcriptomic analysis. While scRNA-seq enables the characterization of cell heterogeneity at an unprecedented resolution, analytical issues like sparsity, noise and bias can severely compromise interpretation if not addressed properly. To extract meaningful biological signals, effective feature selection is critical. We propose Mcadet, a novel framework for feature selection in scRNA-seq data. Mcadet aims to accurately identify informative genes from fine-resolution datasets and datasets with minority cell types where existing methods falter.

Through comparative analysis on diverse simulated and real-world datasets, we demonstrate Mcadet’s superior performance in selecting highly informative genes based on many evaluation metrics in different scenarios, by effectively isolating intrinsic variation and allowing multi-resolution community detection.

By improving the process of feature selection of scRNA-seq data, Mcadet makes it easier for researchers from various backgrounds to work with scRNA-seq data. This means that people can benefit from more reliable and accessible insights into single-cell transcriptomics.

## 1. Introduction

### 1.1 Background

Single-cell RNA sequencing has emerged as a powerful tool for characterizing complex human or animal tissues and cell types, enabling the examination of RNA expression differences at a single-cell resolution. Recent advancements in scRNA-seq techniques have significantly contributed to our understanding of biological systems by allowing simultaneous measurement of transcript levels in thousands of individual cells **[1,2]**. This approach overcomes the limitations of bulk RNA sequencing, which lacks individual resolution.

A crucial component of scRNA-seq analysis is the expression matrix, which represents the number of transcripts detected for each gene and cell. The analysis workflow of scRNA-seq data can be divided into two main sections: pre-processing and downstream analysis. Pre-processing involves quality control, normalization, data correction, feature selection, and visualization, while downstream analysis includes clustering, differential expression analysis, annotation, and gene dynamics analysis **[3,4]**. However, scRNA-seq data face challenges such as high dropout rates, noise, and technical variabilities, which can impede downstream analysis **[5]**. Additionally, scRNA-seq datasets often contain a large number of genes, many of which may not provide relevant information for a specific analysis. Therefore, feature selection plays a vital role in working with multidimensional scRNA-seq datasets.

Numerous methods have been proposed for the selection of HVGs in scRNA-seq data. However, these methods often exhibit limited agreement in the genes identified as highly variable and demonstrate inconsistent clustering performance when applied to different datasets. Currently, there is no consensus on which method outperforms the others. In a study conducted by Yip et al. **[6]**, a comparison of seven feature selection methods for scRNA-seq data revealed substantial differences among the approaches, with each tool demonstrating optimal performance under different scenarios. This highlights the need to carefully consider the choice of feature selection method based on the specific characteristics and objectives of the dataset being analyzed. Furthermore, existing methods are limited to fixed resolutions and lack the ability to handle fine-resolution datasets, which are more difficult to distinguish in terms of biological functions and specific gene expressions compared to coarse-resolution scRNA-seq datasets, details are described in **Sec. 4.1**.

In this paper, we introduce Mcadet as a novel feature selection framework inspired by Cell-ID **[7]** and corral **[8]**. Cell-ID is a gene signature extraction method that enhances the biological interpretation at the individual cell level, enabling the identification of novel cell types or states. On the other hand, corral is a dimensionality reduction technique specifically designed for scRNA-seq data, demonstrating superior clustering performance compared to the standard correspondence analysis (CA) and glmPCA approaches. Both of the methods rely on CA or MCA, which is a statistical technique utilizing singular-value decomposition (SVD). This technique enables the simultaneous projection of individuals and variables into a shared low-dimensional space, making it suitable for non-negative, count-based data to investigate the relationship between samples and categorical variables. CA has a rich historical background, and numerous variations and extensions have been developed for diverse research contexts across various fields **[9,10]**. However, its utilization in scRNA-seq data analysis has been relatively limited until recently when the two aforementioned papers introduced its application. Therefore, it is highly valuable to explore the potential of applying CA in this particular field.

Mcadet utilizes MCA and Leiden community detection to select informative genes from scRNA-seq data and facilitate cell population recovery. Our framework aims to accurately select informative genes, handle fine-resolution datasets and minority cell populations, and allow parameter customization for various resolutions, thus addressing the limitations of current approaches.

In order to evaluate its performance, we conducted a comparative analysis of Mcadet against seven established feature selection methods for scRNA-seq data, as outlined in **Sec. 4.3**. Our assessment involved applying these feature selection methods to a variety of simulated and real-world datasets. We employed several evaluation metrics, such as the Jaccard similarity index for accuracy, silhouette score, adjusted Rand index, and other measures for assessing clustering performance. Our results demonstrate that Mcadet outperforms other methods in terms of the quality of selected genes, particularly in fine-resolution or difficult-to-differentiate scRNA-seq datasets. Overall, our novel feature selection framework, Mcadet, offers an improved toolbox for scRNA-seq analysis, providing more accurate feature selection and enhanced capabilities for handling challenging scRNA-seq datasets.

In **Sec. 1.3**, we begin by presenting an in-depth review of existing feature selection approaches for scRNA-seq analysis. The results of the feature selection performance comparison on both real-world and simulated datasets are displayed in **Sec. 2**. In **Sec. 3**, we conclude the study with a discussion on the proposed feature selection framework, highlighting its advantages and limitations. Finally, in **Sec. 4**, we detail the datasets employed, encompassing both simulated and real-world datasets as well as the workflow of Mcadet, the approach to conducting the feature selection method comparison, and the description of evaluation metrics are presented.

### 1.2 Overview of Mcadet

**Fig 1:**
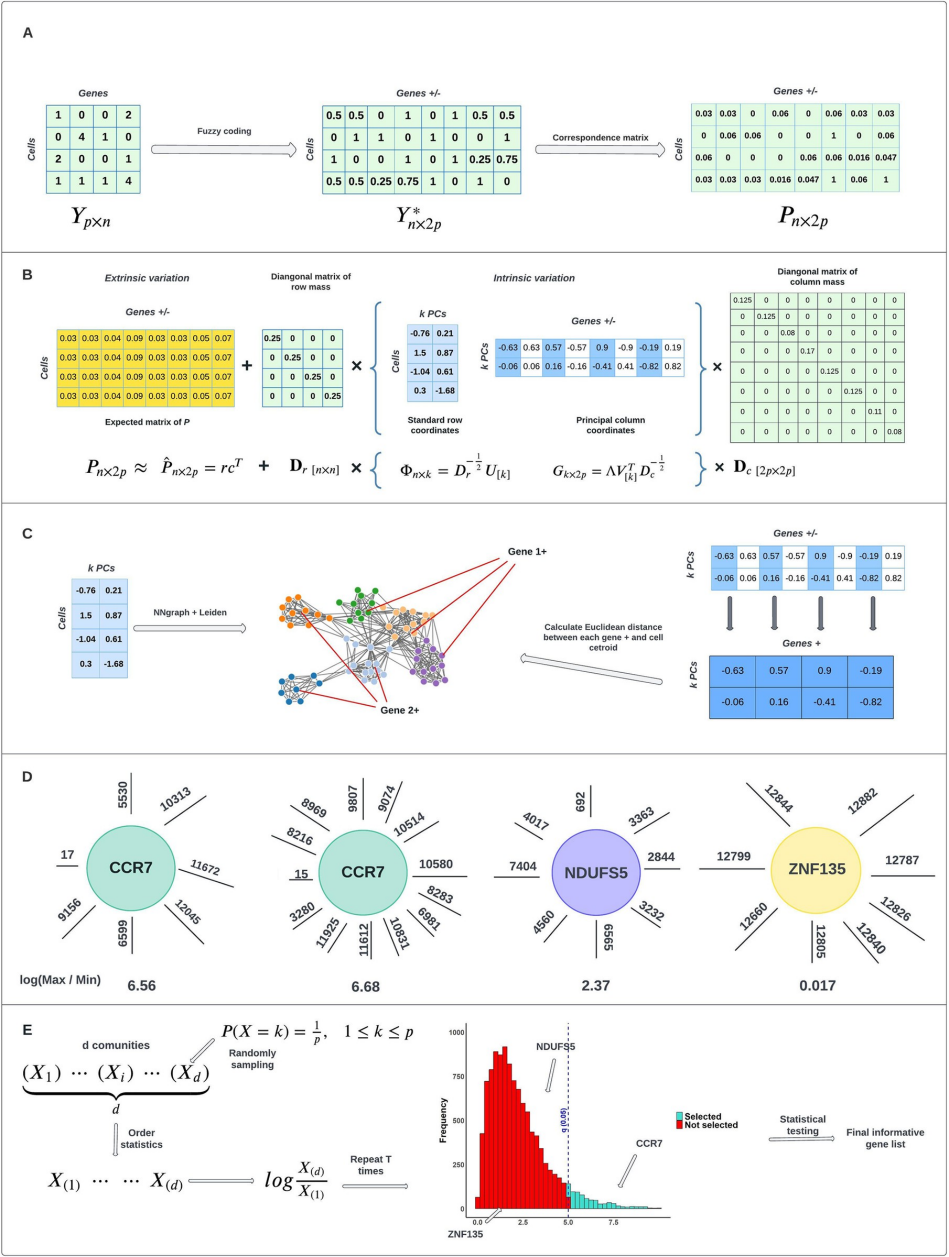
Schematic of Mcadet workflow. A. Matrix pre-processing. B. MCA decomposition. C. Leiden community detection (clustering). D. Calculate and rank Euclidean distances. E. Statistical testing.

**Fig 1** summarizes our feature selection framework. In general, our method encompasses five major steps for analysis:

#### A. Matrix Pre-processing

In this step, we utilize fuzzy coding to double the column variables, transforming an *n*×*p* matrix into an *n*×2*p* matrix, where *n* represents the number of cells and *p* represents the number of genes. Additionally, a correspondence matrix ***P*** is constructed. Detailed procedures are available in **Sec. 4.2**.

#### B. Multiple Correspondence Analysis

We decompose the Pearson residual matrix to extract intrinsic variation, which includes standard row coordinates and principal column coordinates. The detailed methodology for this decomposition is provided in the **Sec. 4.2**.

#### C. Calculate and Rank the Distances

Both the standard row coordinates of cells and the principal column coordinates of genes are projected into the same embedded space using the top *k* principal components. We apply the Leiden algorithm to partition the cells into clusters. Subsequently, we calculate the centroid coordinates for each cell cluster and determine the Euclidean distances between each cell cluster and each gene. In the resulting visualization, dots represent cells, text labels represent genes, and different colors indicate distinct cell clusters. Red straight lines illustrate the Euclidean distances. The proximity of a variable (column) to an observation (row) in the Euclidean space of the reduced dimensional MCA biplot suggests a stronger association between the variable and the observation. This property is substantiated in the **Sec. 4.2**.

#### D. Create New Metric

We introduce a new metric by calculating the log ratio between the maximum rank and the minimum rank to represent gene variability. For instance, “CCR7” is a well-known driver gene for Naive CD4+ T cells. In our example in Fig 1 panel D (left), it ranks 17th in Euclidean distance among all available genes to a particular cell cluster. We do not need to know the exact cell cluster, as feature selection is blind and does not require knowledge of which gene is informative or a driver for a specific cell type. The maximum rank for "CCR7" is 12,045, indicating that it is very close to one cell cluster but far from another, making it potentially informative for a specific cell type. The new metric value for "CCR7" is a relatively high 6.56. Conversely, the "NDUFS5" gene, a housekeeping gene expressed in many cell types, shows a uniform rank pattern. "ZNF135," an irrelevant gene expressed in only a few cells, ranks above 12,000 for all clusters. The statistic of log(Max rank/Min rank) is used for gene selection. Next to the left diagram of gene “CCR7”, there is another diagram which includes more distances, indicating more detected clusters. This difference arises from inputting different resolution parameters into the Leiden algorithm, which detects varying numbers of clusters. Despite changing the resolution, "CCR7" consistently ranks 15th to a specific cluster, demonstrating its close proximity to a certain cell cluster regardless of the specific cluster identity.

#### E. Statistical testing

The final step involves statistical testing. We assume that each gene’s rank pattern *X*_*j*_ is independently and identically distributed, following a discrete uniform distribution. Under this assumption, the null hypothesis posits that the gene in question is not informative, implying that its distance is ranked uniformly across all cell clusters. Conversely, the alternative hypothesis suggests that the gene does not follow this uniform distribution but instead follows another distribution that may indicate its informativeness to a particular cell cluster. The derivation of the statistical distribution is detailed in the **Sec. 4.2 and S1 Methods**. By evaluating p-values, we can reject the null hypothesis and identify a list of informative genes. The Benjamini-Hochberg (BH) procedure is applied to control the false discovery rate (FDR), ensuring the robustness of our selection process.

### 1.3 Overview of Other Feature Selection Methods

We provide a comprehensive review of feature selection approaches used in existing tools and software for scRNA-seq analysis, as summarized in **Table 1**. These approaches can be categorized into three main types: dispersion-based, dropout-rate-based, and machine-learning-based. Initially, dispersion-based methods were proposed and incorporated into scRNA-seq analysis tools such as Brennecke **[11]**, scran **[12]**, scVEGs **[13]**, Seurat **[14]**, Scry **[15]**, sctransform **[16,17]**, and Scanpy 1.9. The method introduced by Brennecke et al. **[11]** involves fitting a generalized linear model to a plot of the mean and squared coefficient of variation (CV2). This model enables the derivation of a coefficient of biological variation, and a chi-square test is then used to identify genes with high variance in their coefficient of biological variation, indicating potential variability in gene expression. Subsequent modifications led to the development of different feature selection methods, such as those implemented in scran, scVEGs, Seurat, and Scry. In the case of scran, HVGs are determined by employing local polynomial regression (LOESS) on the mean-variance relationship of log-transformed expression values. scVEGs assumes a negative binomial distribution for the mean versus coefficient of variation (CV) relationship and fits this relationship through modified local regression and nonlinear least squares curve fitting. Consequently, parameters of the gene variation model are estimated, enabling the identification of statistically significant variably expressed genes. Within the Seurat tool, three feature selection methods are available. The “vst” method fits a line to the relationship between log variance and log mean using LOESS, similar to scran. The “mvp” method utilizes the Fano factor (variance divided by mean) and categorizes Fano factors into 20 expression mean-based bins. It then normalizes the Fano factors in each bin into z-scores and selects genes accordingly. The “disp” method selects genes with the highest dispersion values. Scry calculates a deviance statistic for counts based on a multinomial model assuming a constant rate for each feature. Hafemeister et al. and Choudhary et al. **[16,17]** proposed sctransform method, which uses Pearson residuals from “regularized negative binomial regression,” where cellular sequencing depth is used as a covariate in a generalized linear model, effectively remove the impact of technical characteristics from downstream analyses while preserving biological heterogeneity. Additionally, Lause et al. **[18]** demonstrate that the model of sctransform generates noisy parameter estimates due to overspecification, resulting in biased per-gene overdispersion estimates. They show that the data are consistent with the overdispersion parameter being independent of gene expression. Using negative control data devoid of biological variability, Lause et al. [18] estimate the technical overdispersion of UMI counts and propose the use of analytic Pearson residuals. They further demonstrate that analytic Pearson residuals perform well in identifying biologically variable genes and capture more biologically meaningful variation when used for dimensionality reduction. All methods mentioned above are variance-to-mean approaches that aim to measure dispersion or noise-to-signal ratio. However, they may inadvertently select many low-expression genes due to a high rate of dropouts.

**Table 1:** Selected existing feature selection methods in scRNA-seq analysis.

FS method	Quantities/model description*	Type	Year and Reference
Brennecke	μ and CV^2^ (glm)	Dispersion	2013 [11]
Seurat Vst	μ and δ^2^ (loess)	Dispersion	2015 [12]
Seurat Mvp	Fano factor (20 bins)	Dispersion	2015 [12]
Seurat Disp	log (δ^2^/ μ)	Dispersion	2015 [12]
scran	μ and δ^2^ (loess)	Dispersion	2016 [13]
scVEGs	μ and CV (loess)	Dispersion	2016 [14]
Scry	Deviance (Multinomial)	Dispersion	2019 [15]
sctransform	Pearson residuals	Dispersion	2019 [16,17]
Scanpy 1.9	Analytic Pearson residuals	Dispersion	2021 [18]
M3drop	Michaelis-Menten	Dropout rate	2019 [19]
NBdrop	Negative binomial	Dropout rate	2019 [19]
FEAST	consensus cell clustering and F-test	Machine learning	2021 [22]
DUBStepR	Stepwise regression	Machine learning	2021 [23]
Triku	NNgraph and Wasserstein distance	Machine learning	2022 [24]

*Mean and variance are denoted as μ and δ^2^. Coefficient of variation is denoted as CV, which is σ/μ.

To address this issue, Andrews et al. **[19]** introduced two new feature selection methods, M3drop and NBdrop, which are based on dropout rates. M3drop is designed for read counts from full-transcript sequencing protocols (e.g., SmartSeq2), while NBdrop is suitable for counts of UMIs from tag-based protocols (e.g., 10X Chromium), both methods can be applied to scRNA-seq count data regardless of the sequencing technology. The key distinction between the two methods lies in their underlying statistical models: M3Drop fits a Michaelis-Menten model to describe the relationship between mean expression and dropout rate, whereas NBDrop employs a negative binomial model for the same purpose. Both methods operate under the assumption that genes with a higher proportion of zeros than expected could be biologically significant since they may be expressed in fewer cells than anticipated. These cells may correspond to specific cell types or states.

Recently, novel feature selection techniques for analyzing scRNA-seq data have emerged, incorporating machine learning and graph-based algorithms. These methods, namely FEAST **[20]**, Triku **[21]**, and DUBStepR **[22]**, differ from the model-based approaches as they leverage machine learning techniques. FEAST begins by obtaining a consensus cell clustering and then evaluates the significance of each feature using an F-test, ranking the features based on the F-statistics. Triku identifies genes that are locally overexpressed in neighboring cell groups by examining the count distribution in the vicinity of each cell and comparing it to the expected distribution. The calculation involves determining the Wasserstein distance between the observed and expected distributions, and genes are ranked based on this distance. A larger distance indicates localized expression in a subset of cells with similar transcriptomic profiles. DUBStepR is a stepwise procedure for identifying a core set of genes that strongly reflect coherent expression variation in a dataset. The authors propose a novel graph-based measure for aggregating cells in the feature space, optimizing the number of features based on this measure. We recognized the general usefulness of machine learning techniques in the field of scRNA-seq data analysis, particularly for feature selection. In this study, we selected seven existing feature selection methods for comparison: Brennecke, Scry, M3drop, NBdrop, Seurat Vst, Seurat Mvp, and Seurat Disp.

## 2. Results

In feature selection, the main goal is to identify the most relevant features that effectively describe and understand datasets. Specifically, we approached the results from three aspects. First, the quality of the selected HVGs, meaning we know the ground truth or semi-ground truth of HVGs. By comparing the genes selected by different feature selection methods using Jaccard similarity, we can determine which method yields genes most closely matching the true HVGs. Additionally, we conducted in-depth studies on the quality of genes by (1) comparing the performance of feature selection methods under different numbers of selected genes, (2) evaluating the performance of these methods on scRNA-seq datasets associated with minority cells, (3) assessing the consistency of feature selection within the same dataset, (4) comparing the log-mean gene expression of the selected HVGs, and 5) investigating the HVGs uniquely selected by Mcadet but not by other feature selection methods. Second, we evaluated whether the selected genes contribute to downstream analysis, specifically clustering and trajectory inference. Third, we made subjective judgments based on 2-D visualizations. We used various evaluation metrics, which are detailed in **Sec. 4.3**. In terms of datasets, we utilized both real-world peripheral blood mononuclear cells (PBMC) datasets and simulated datasets, each available in two resolutions: coarse and fine. Therefore, there are 4 dataset types, coarse-resolution PBMC datasets, fine-resolution PBMC datasets, coarse-resolution simulated datasets, and fine-resolution simulated datasets. Detailed information about these datasets can be found in **Sec. 4.1**.

### 2.1. The quality of the selected HVGs

#### 2.1.1 Jaccard similarity

**Fig 2:**
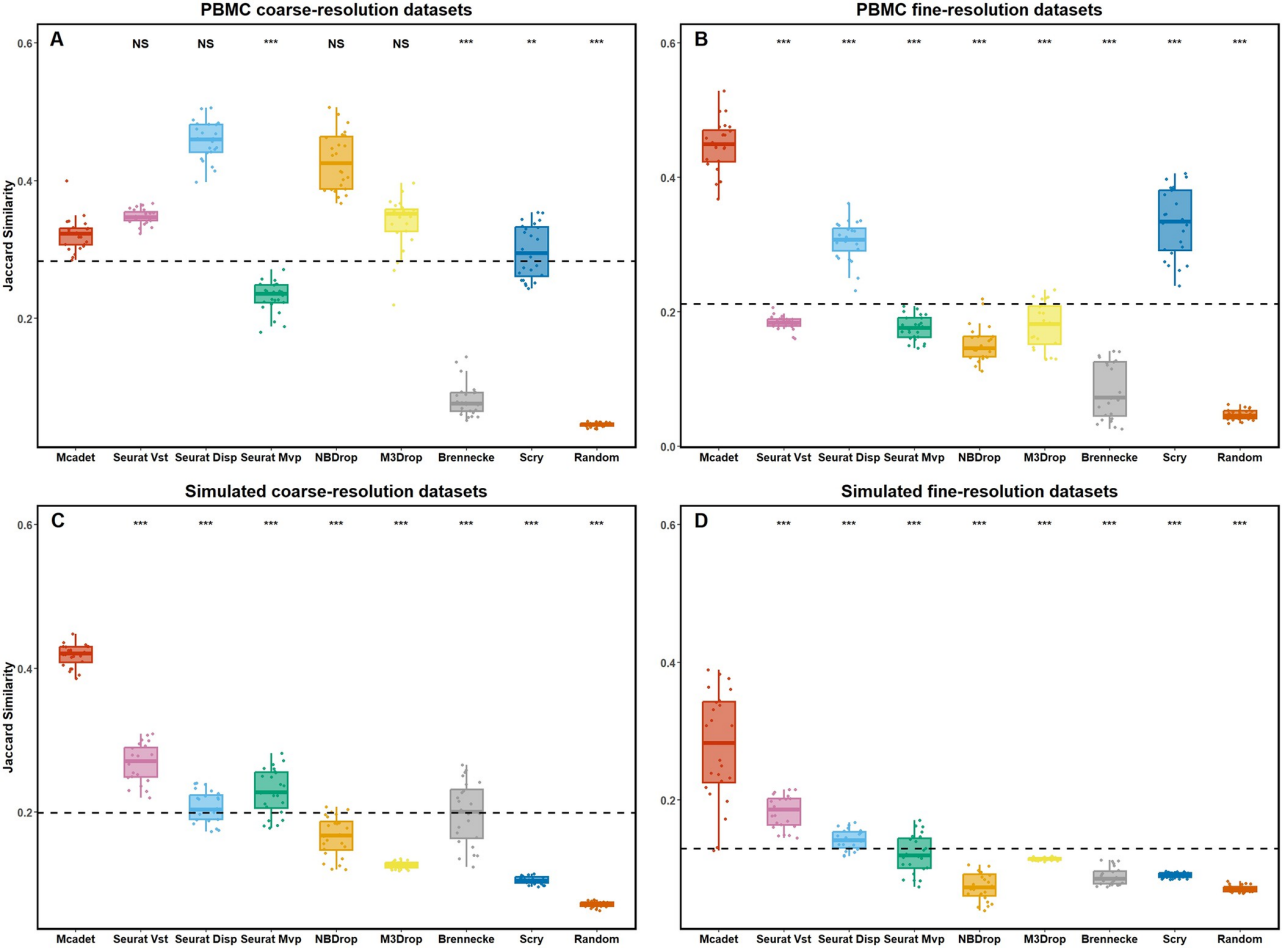
**Jaccard Similarity index for comparing feature selection performance on PBMC (A and B) and simulated datasets (C and D).** The Jaccard similarity index measures the accuracy of selecting true (or semi-true) HVGs by different FS methods. Each dot in the graph represents a dataset, and the dashed horizontal line represents the baseline mean for all methods. The p-values, obtained through one-sided pairwise t-tests, indicate the significance of the differences between Mcadet method and each other FS method. Ns: non-significance, NS: p ≥ 0.05,*: 0.01 ≤ p < 0.05, **: 0.001 ≤ p < 0.01, ***: p < 0.001.

In our study, we first conducted a comparison between our method and other feature selection methods using Jaccard similarity index to evaluate the accuracy of feature selection in selecting HVGs. **Fig 2** presents the boxplot of the Jaccard similarity by different feature selection methods across various datasets. Each dot represents a dataset from four different dataset types, the dashed horizontal line represents the baseline mean for all methods. **Fig 2B–2D** provides compelling evidence that our method achieves a significantly higher mean Jaccard similarity compared to all other Feature Selection (hereafter referred to as FS) methods in the type of PBMC fine-resolution, simulated coarse-resolution and simulated fine-resolution datasets (p < 0.001, one-sided pairwise t-test). Although our method does not outperform other methods like Seurat and NBDrop in terms of Jaccard similarity in PBMC coarse-resolution datasets (**Fig 2A**), it emerges as the top-performing method for the other types of datasets, particularly fine-resolution datasets. This outcome highlights a noteworthy enhancement in feature selection accuracy for challenging or difficult-to-differentiate fine-resolution datasets.

#### 2.1.2 The number of selected HVGs

**Fig 3:**
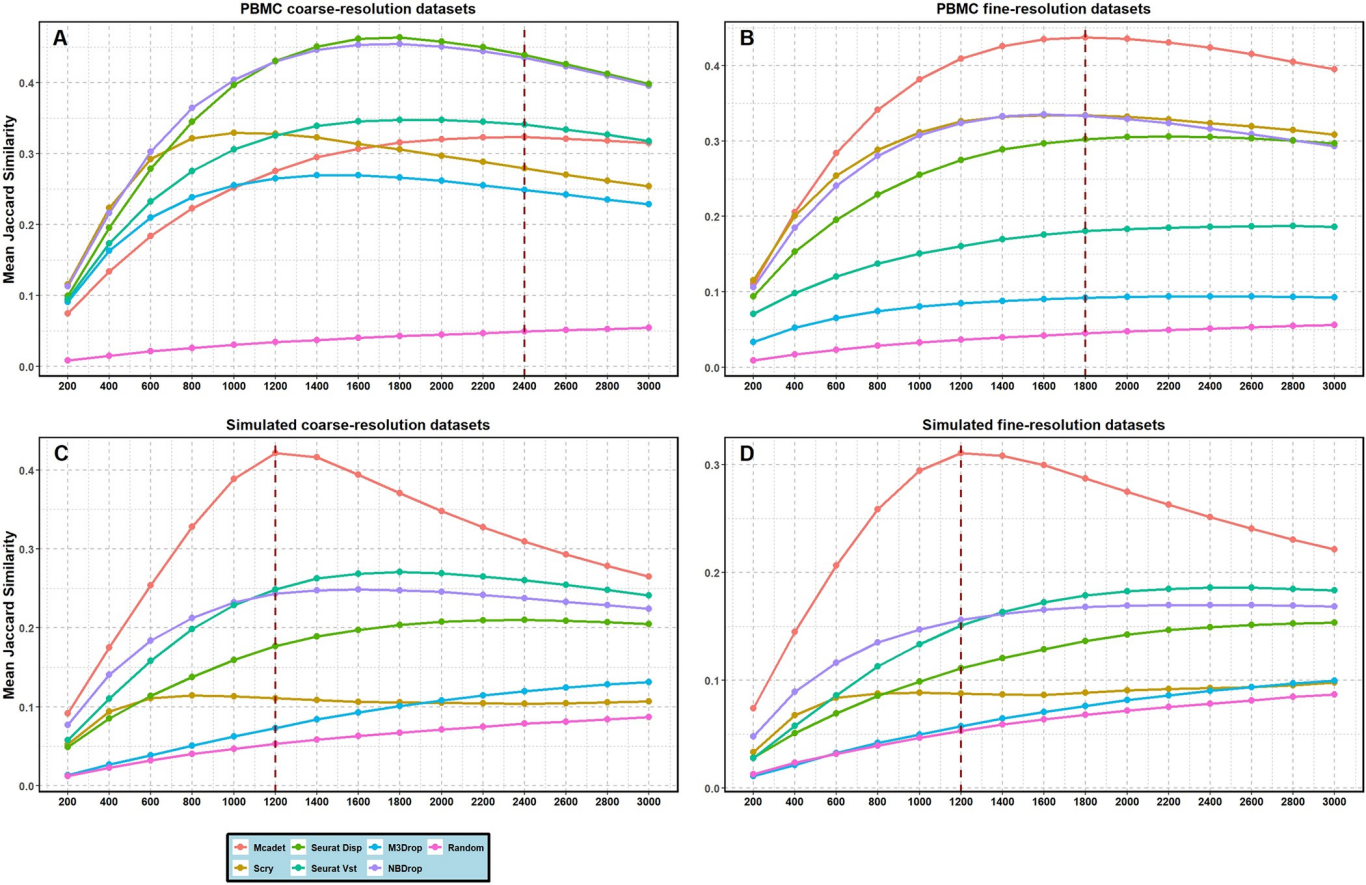
**The trend of Jaccard Similarity index as the number of selected genes increases on PBMC (A and B) and simulated datasets (C and D).** The number of selected genes range from 200 to 3,000. The Brennecke method, which does not allow for specifying the number of HVGs needed, are excluded from this comparison.

There is a consensus within the field that the feature selection step in scRNA-seq data analysis should typically involve selecting between 1,000 and 3,000 genes [4]. In a data-driven scenario, the number of informative genes can vary across different datasets, and users may not have prior domain knowledge to determine the exact number of informative genes required. In such cases, our method, along with some existing methods, offers users the opportunity to select informative genes using statistical testing (p-values) or other threshold criteria by default. **Fig 3** presents the trend of mean Jaccard similarity as the number of selected genes increases from 200 to 3,000 by different FS methods and across the four types of datasets.

The dashed vertical line indicates the number of selected genes that maximizes the Jaccard similarity index when Mcadet exhibits the best performance value. Observing the graphs, we note that the Jaccard similarity index of our method generally increases up to around 1,800 selected genes and then begins to decrease for PBMC fine-resolution datasets. Similarly, for simulation coarse- and fine-resolution datasets, the Jaccard similarity index increases until approximately 1,200 selected genes and then declines. Overall, our method outperforms other methods included in this section for these three types of datasets. However, our method does not perform as well as other methods for PBMC coarse-resolution datasets, which aligns with our earlier result that Mcadet performs better in handling fine-resolution datasets compared with coarse-resolution ones.

We conducted a comparison of the default number of informative genes selected by different feature selection methods, and the results are presented in **Tables 2** and **S1**. Due to the lack of default parameters for selecting highly HVGs in Seurat Vst, Disp, Scry, and random selection methods, we set the default number of genes to 2,000 for these methods. For the 24 PBMC coarse-resolution datasets, the median number of HVGs selected across all methods is 1,662 (IQR: [1640, 1725]). Similarly, for the 24 PBMC fine-resolution datasets, the median number of HVGs selected is 1,615 (IQR: [1423, 12315]). In the case of simulated datasets, the default number of selected genes is fixed at 2,000 for all methods. Our method yields a close match to the true number of HVGs for PBMC fine-resolution datasets (1,407 vs. 1,615). However, for PBMC coarse-resolution datasets, our method selected slightly more genes (2,555 vs. 1,662). The details for simulated datasets are in **S1 Table**, the number of genes selected by Mcadet is less than the true number.

**Table 2:** Number of HVGs selected by different feature selection methods by default.

PBMC	Coarse-resolution datasets 1662 (1640, 1725)		Fine-resolution datasets 1615 (1423, 2315)	
	N Median (IQR)	Jaccard Similarity Median (IQR)	N Median (IQR)	Jaccard Similarity Median (IQR)
Mcadet	2555 (2366, 2718)	0.32 (0.31, 0.33)	1407 (1291, 1848)	0.45 (0.42, 0.47)
NBDrop	1362 (967, 1673)	0.43 (0.39, 0.46)	257 (233, 449)	0.15 (0.13, 0.16)
M3Drop	1594 (1023, 2724)	0.35 (0.33, 0.36)	790 (378, 2112)	0.18 (0.15, 0.21)
Brennecke	5573 (2908, 10603)	0.08 (0.06, 0.09)	1272 (707, 3244)	0.07 (0.04, 0.12)
Seurat Mvp	583 (508, 651)	0.24 (0.22, 0.25)	551 (452, 847)	0.18 (0.16, 0.19)
Seurat Vst	2000 (2000, 2000)	0.35 (0.34, 0.35)	2000 (2000, 2000)	0.18 (0.18, 0.19)
Seurat Disp	2000 (2000, 2000)	0.46 (0.44, 0.48)	2000 (2000, 2000)	0.30 (0.29, 0.32)
Scry	2000 (2000, 2000)	0.29 (0.26, 0.33)	2000 (2000, 2000)	0.33 (0.29, 0.38)
random	2000 (2000, 2000)	0.05 (0.04, 0.05)	2000 (2000, 2000)	0.04 (0.04, 0.05)

Moreover, the corresponding median Jaccard index obtained from Mcadet is higher compared to the other methods in PBMC fine-resolution and all simulated datasets, indicating a higher accuracy in selecting informative genes.

#### 2.1.3 For datasets with minority cell types

**Fig 4:**
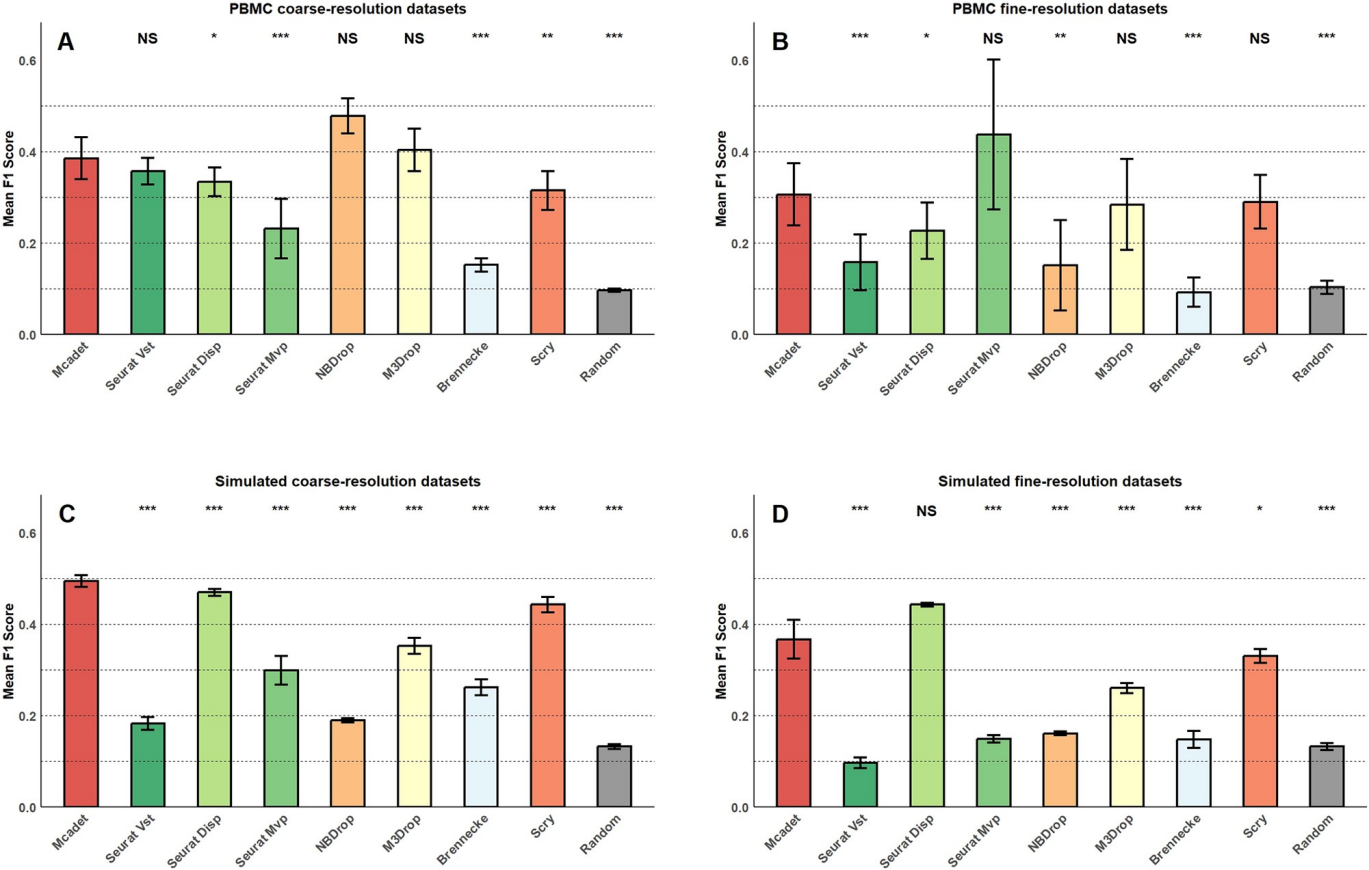
**F1 score: Performance evaluation on minority cell populations on PBMC (A and B) and simulated datasets (C and D).** The p-values, obtained through one-sided pairwise t-tests, indicate the significance of the differences between Mcadet method and each other FS method. Ns: non-significance, NS: p ≥ 0.05,*: 0.01≤ p < 0.05, **: 0.001 ≤ p < 0.01, ***: p < 0.001.

To evaluate the performance of the feature selection method in selecting HVGs for minority cell populations, we incorporated predefined minority cell populations in each dataset. In the case of the 48 simulation datasets (including both coarse- and fine-resolution datasets), cell type 1 was specifically designated as the minority cell type (as shown in **S2 Table**). For each dataset, cell type 1 consisted of only 60 cells (1%) out of a total of 6,000 cells. Additionally, 620 genes were designed as HVGs for cell type 1. In the case of the 24 coarse-resolution PBMC datasets, we deliberately selected sixty B cells from each original dataset to establish B cells as the minority cell type. Subsequently, feature selection was performed on these datasets. The semi-true HVGs for B cells were obtained using the “FindAllMarkers (cluster = ‘B’)” function in the Seurat package. As for the remaining 24 fine-resolution PBMC datasets, dnT cells were identified as the minority cell type. The semi-true HVGs for dnT cells were obtained using the “FindAllMarkers (cluster = ‘dnT’)” function in the Seurat package. The evaluation metric used here is the F1 score instead of Jaccard similarity. Because Jaccard similarity ($J=\frac{\left|A\cap B\right|}{\left|A\cup B\right|}$) is used to evaluate the similarity between two comparable sets, if *A* is the set of all (semi-) true HVGs and *B* is the set of HVGs selected by feature selection methods, then they are comparable and the Jaccard similarity can be used, as seen in the results mentioned above. However, in this experiment, *A* is merely the set of true HVGs for a minority cell type, which is a subset of *A* mentioned above. Therefore, using the Jaccard similarity might not be appropriate in this context. Here, recall is defined as |*A*∩*B*|/|*A*|, and precision as |*A*∩*B*|/|*B*|, recall can be cheated by simply selecting more HVGs (e.g., selecting all genes would result in a perfect score). Precision can also be misleading in some cases. For example, in **S1 Table**, methods like NBDrop and Seurat Mvp have very small |*B*|, which leads to inflated precision. A method with a high precision might be interpreted as favoring the selection of HVGs from the minority cell type, while ignoring those from other cell types. To strike a balance, we decided to use the F1 score, which is the harmonic mean of recall and precision. **Fig 4** illustrates the results of the minority cell analysis. Each bar represents the mean F1 score of all datasets within the scenario by each FS method. Overall, our method consistently ranks among the top three in all four data types, indicating its excellent performance in identifying informative genes for minority cell populations. Specifically, NBdrop and M3Drop performed better on PBMC coarse-resolution datasets. However, their F1 scores dropped significantly on PBMC fine-resolution datasets. Mcadet ranked among the top two in simulated datasets, with Seurat Disp and Scry also showing relatively good performance.

#### 2.1.4 Consistency within the same dataset

An additional vital factor to consider when evaluating the effectiveness of a FS method is its consistency within the same sample. Put simply, if a scRNA-seq dataset is randomly divided into two separate datasets by gene expression value with a specific probability, a good FS method will yield similar HVGs to the (semi-) true HVGs. To perform the dataset division, we employed a random splitting method, evenly dividing the datasets into two subsets. The concept of this splitting technique draws inspiration from a method known as count splitting **[23]**. This was achieved by generating *X*_(1),*ij*_~*Binom*(*X*_*ij*_, *ε*), where *X*_*ij*_ represents the expression value of gene *j* in cell *i* from the original scRNA-seq dataset. Here, *ε* denotes the probability. Consequently, *X*_(1),*ij*_ corresponds to the expression value of gene *j* in cell *i* within the first split dataset. The expression value of gene *j* in cell *i* within the second split dataset, denoted as *X*_(2),*ij*_, is calculated as *X*_*ij*_−*X*_(1),*ij*_.

**Fig 5:**
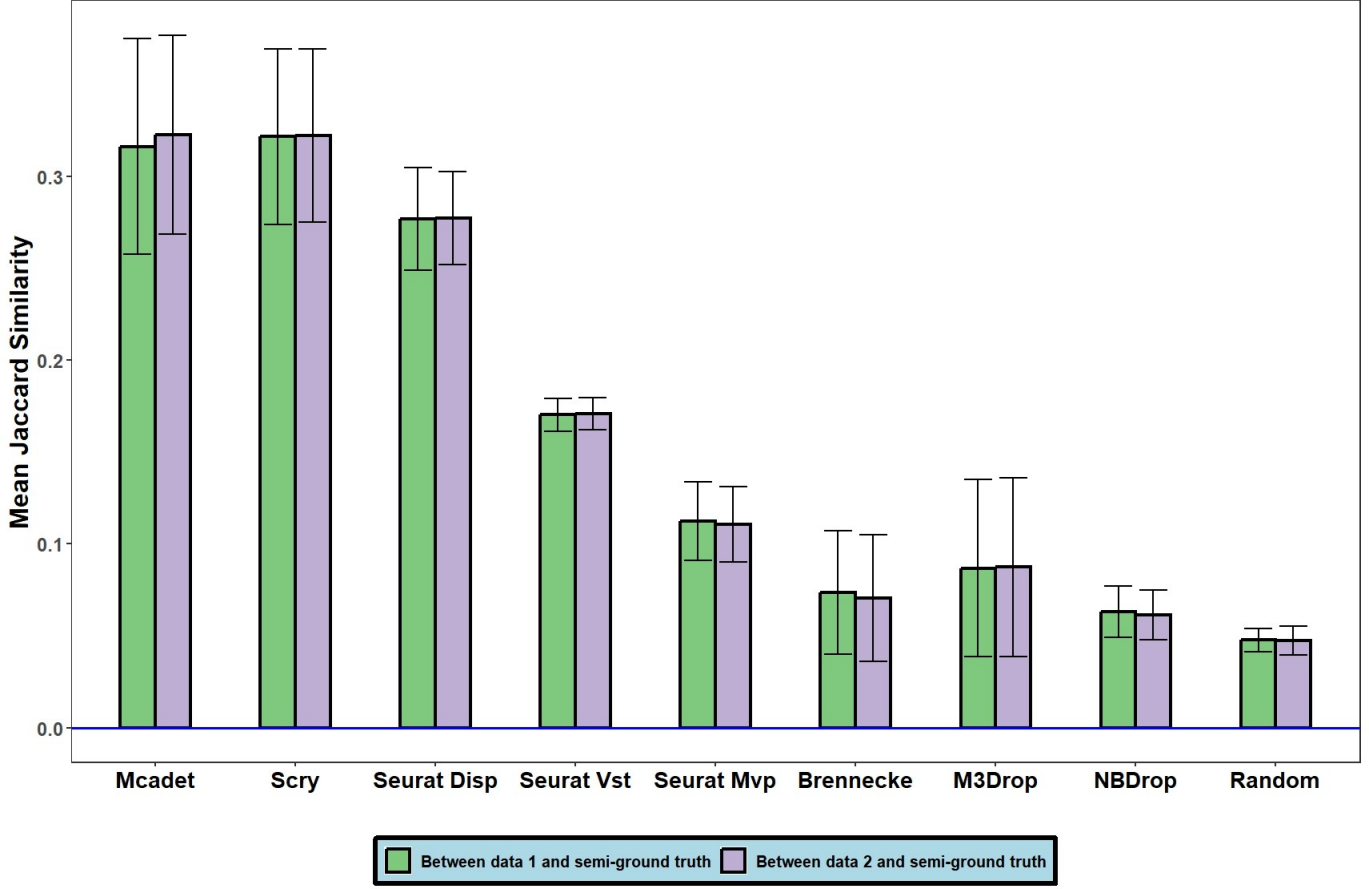
Comparison of the mean Jaccard similarity of genes selected by different FS methods with semi-true HVGs in PBMC fine-resolution datasets after splitting with a probability of *ε* = 0.5. Error bars represent the standard deviations. Data 1 and 2 are the two split datasets of original datasets.

**Fig 5** demonstrates the comparison between genes selected by different FS methods and semi-true HVGs in PBMC fine-resolution datasets after splitting with a probability of *ε* = 0.5. Data 1 and 2 represent the two split datasets of the original datasets. It shows that Mcadet and Scry achieve the highest Jaccard similarity, indicating their capability to handle lower sequencing depth. We also included other splitting probabilities, **S1 Fig** presents different scenarios with splitting probabilities from 0.1 to 0.4. For instance, in the top panel, the left bar represents the dataset with about 0.1 times of original gene expressions, and the right bar represents the dataset with 0.9 times of that. We observe that as the split becomes more uneven from 0.4 to 0.1, the differences between Mcadet’s bars increase, which makes sense because as the split becomes more uneven, especially with a 0.1 split, the sequencing depth of the 0.1 dataset becomes very shallow, thus reducing the Jaccard similarity. We find that except for Scry and the random control group, other methods show increased differences in this process. Therefore, Scry appears to handle shallow sequencing depth the best. Mcadet can handle shallow depth with a probability of around 0.4, but its performance significantly declines below 0.3 sequencing depths effectively. This result provides a valuable insight that if the gene expression is artificially reduced due to technical issues like sequencing depth, our method can handle the situation well when the reduction is moderate (1≥*p*≥0.4). However, for more aggressive count splitting (*p*<0.4), other methods such as Scry perform better.

#### 2.1.5. Investigating the expressions of selected HVGs

Another worthwhile analysis is to investigate and analyze the expression density of HVGs selected by different FS methods, comparing it with the expression density of (semi-) true HVGs to see if the agreement or overlap is high.

**Fig 6:**
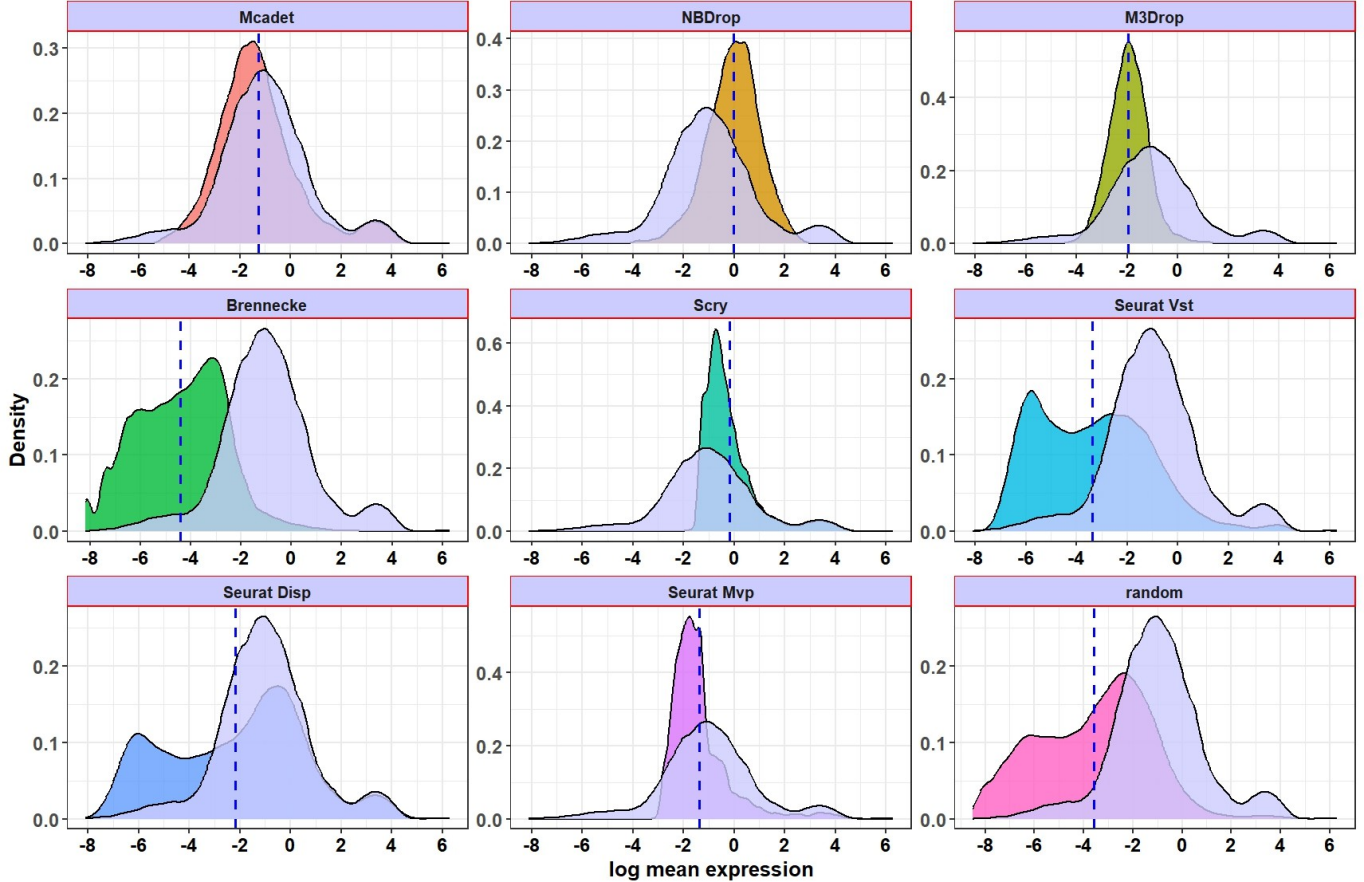
Density plot of log-mean expression for selected genes in PBMC fine-resolution datasets. The light lavender density represents the true HVGs in each panel. The dashed vertical blue line represents the mean for each distribution.

**Fig 6** presents the density plot of the log-mean expression of the pooled selected genes for each FS method in PBMC fine-resolution datasets. The dashed vertical blue line represents the mean for each distribution. The light lavender density in each panel represents the true HVGs. We can see that the density plots for Mcadet and the true HVGs both exhibit a bimodal distribution and are very similar, with the largest overlap. Conversely, NBdrop and Scry tend to favor selecting highly expressed genes. Specifically, Scry shows a high concentration of genes with a particular mean expression value. On the other hand, Brennecke tends to select lowly expressed genes. This also indicates that for particular scRNA-seq data, the (semi-) true HVGs can encompass both highly and lowly expressed genes. It is important to note that certain lowly expressed genes can still hold significant importance in terms of information content. Additionally, the log-mean gene expression density for Seurat Disp and Vst appears to exhibit a trimodal distribution, which differs significantly from that of the true HVGs. This result indicates that our method is balanced in capturing genes across a wide range of expression levels and closely aligns with the gene expression density of semi-true HVGs in PBMC fine-resolution datasets. The figure for PBMC coarse-resolution datasets can be found in **S2 Fig**. However, from that figure, we can observe that, compared to PBMC fine-resolution, the density of genes selected by Mcadet differs more significantly from the corresponding true HVGs density. This also echoes the earlier result that our selection method is particularly useful for fine-resolution datasets.

#### 2.1.6. HVGs selected by Mcadet only

**Fig 7:**
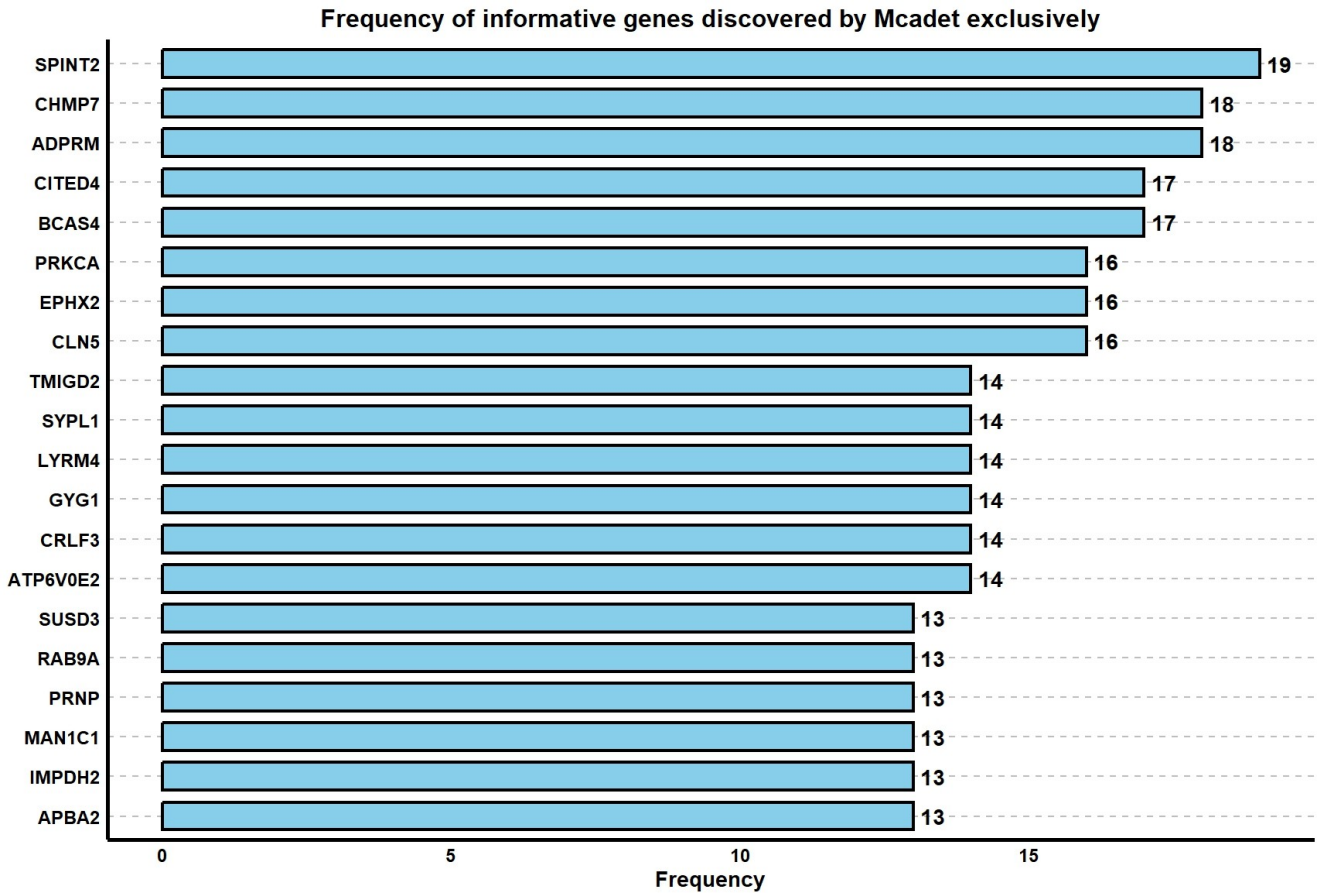
Frequency bar plot of informative genes discovered by Mcadet exclusively.

In this study, we applied the following criteria to identify informative genes selected by Mcadet but not by other feature selection methods in PBMC fine-resolution datasets:

$$D=(A\cap B)-(C_{1}\cup C_{2}\cup C_{3}\cup C_{4}\cup C_{5}\cup C_{6}\cup C_{7})$$

where *A* represents the semi-ground truth of the informative gene set. *B* represents the informative gene set selected by Mcadet. *C*_1_ to *C*_7_ represent the informative gene sets selected by the seven other FS methods included in our study (excluding random selection). This criterion defines set *D* as the gene set containing truly informative genes selected by Mcadet but not selected by any of the other FS methods. By examining set *D*, we can highlight discoveries uniquely enabled by Mcadet. For each PBMC fine-resolution dataset, we obtained a set *D*. We repeated this process across all 24 datasets, aggregated all *D* sets, and calculated the frequencies of the informative genes identified exclusively by Mcadet. The frequency histogram in **Fig 7** displays the top 20 genes with the highest frequencies. For instance, the most frequently identified gene, SPINT2, appeared 19 times out of 24 datasets, indicating its repeated discovery by Mcadet but not by other methods. The interpretation is similar for other genes in the histogram. This approach allows us to demonstrate the unique advantages of Mcadet in identifying significant genes within PBMC fine-resolution datasets.

Next, we selected the top five genes from this frequency plot for further investigation. SPINT2 (Serine Peptidase Inhibitor, Kunitz Type 2) is a gene known for its role in inhibiting serine proteases, and it has been studied primarily in the context of cancer and other diseases. SPINT2’s involvement is broadly related to its inhibitory functions and its impact on cellular processes such as proliferation and migration, which can be relevant across various cell types, including different subsets of T cells **[24,25]**.

**Fig 8:**
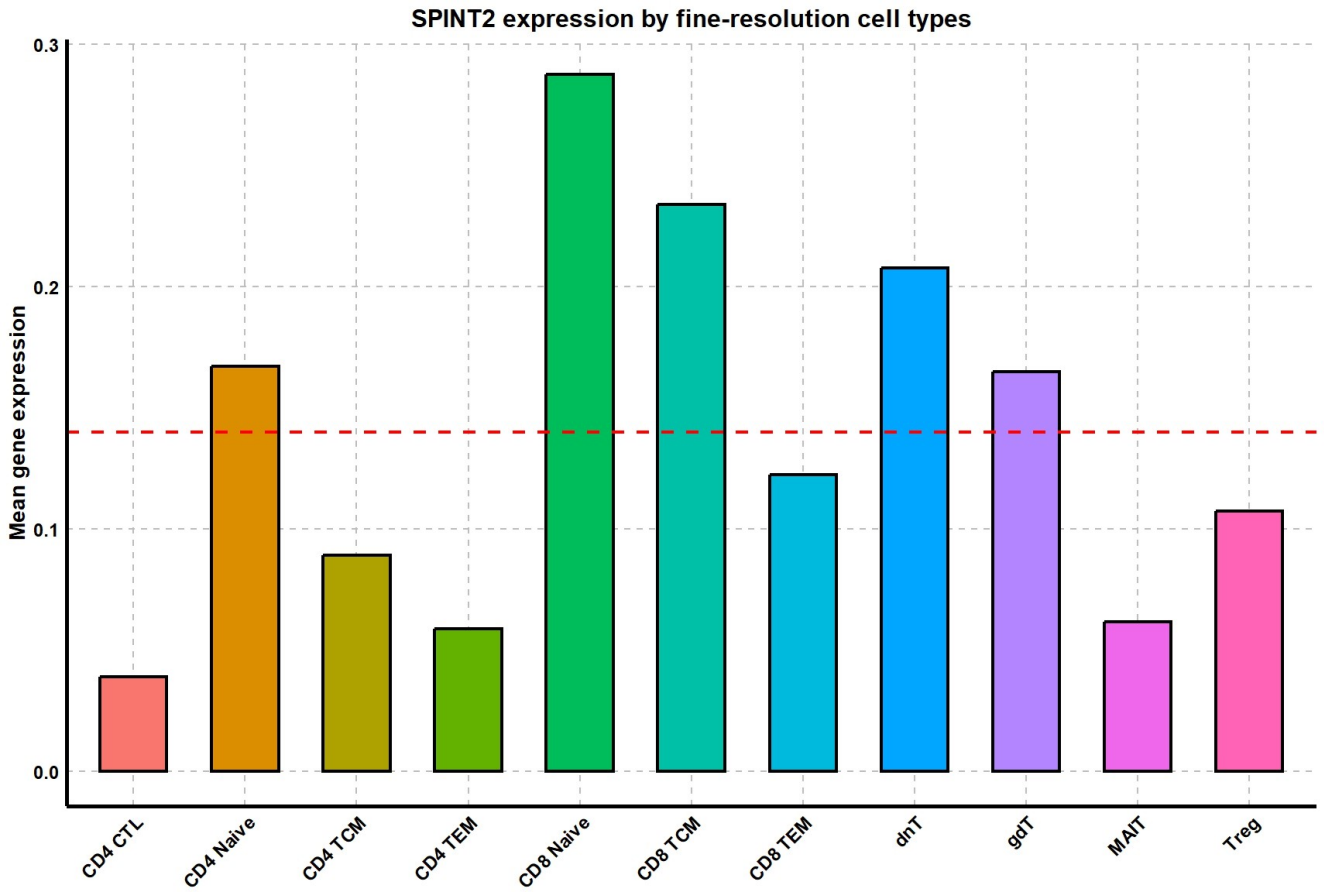
Comparison of the mean gene expression of gene SPINT2 by different fine-resolution PBMC cell types. The horizontal red dashed line represents overall mean.

The bar plot in **Fig 8** illustrates the mean gene expression of SPINT2 across different fine-resolution cell types. Notably, SPINT2 is highly expressed in CD8 naive cells, suggesting its potential relevance to this cell type. Therefore, if this gene is selected, it can serve as a marker to distinguish CD8 Naive cells from other CD8 cells in downstream analyses such as clustering, thereby improving the clustering results. However, there is currently insufficient evidence to classify SPINT2 as a specific marker gene for CD8 naive T cells. Studies on the functional roles and expression profiles of SPINT2 have not highlighted it as significant in the context of CD8 naive T cells. Therefore, we propose that using Mcadet for feature selection in fine-resolution scRNA-seq data can uncover many previously unidentified informative genes. On the other hand, even if the genes selected by Mcadet are not widely recognized as informative genes for a specific cell type, they may still be highly expressed in that cell type in the current dataset. This high expression could be due to various factors such as the donor’s condition, gender, age, or disease status, making these genes worth further attention. This capability is something other feature selection methods might not achieve. For the other four top genes—CHMP7, ADPRM, CITED4, and BCAS4—their details are provided in **S3–S6 Figs**.

### 2.2. Contribution to the downstream analysis

#### 2.2.1 HVGs associated with clustering

**Fig 9:**
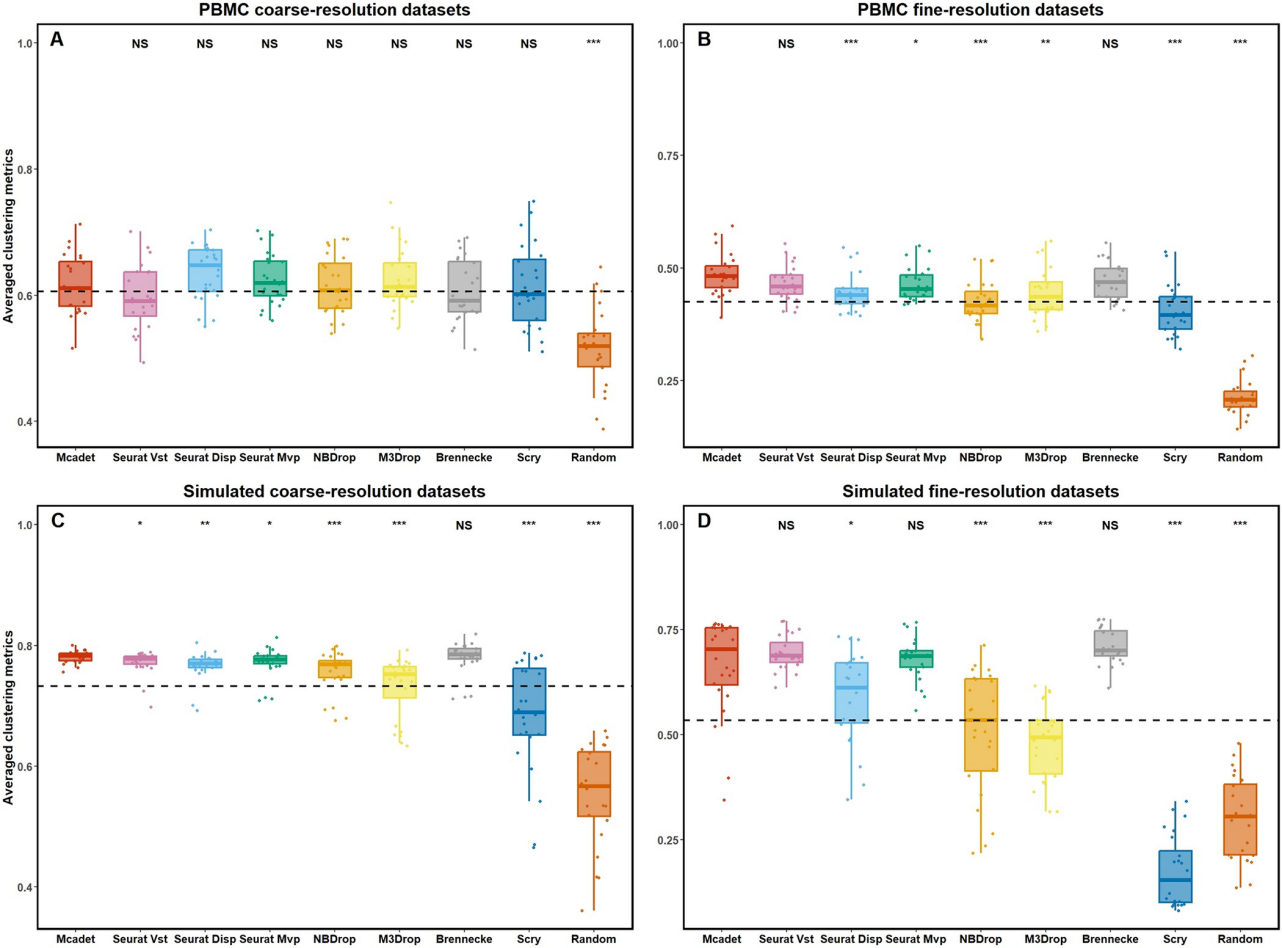
Averaged clustering metrics for comparing feature selection performance on PBMC (A and B) and simulated datasets (C and D). The p-values, obtained through one-sided pairwise t-tests, indicate the significance of the differences between Mcadet method and each other FS method. Ns: non-significance, NS: p ≥ 0.05,*: 0.01 ≤ p < 0.05, **: 0.001 ≤ p < 0.01, ***: p < 0.001.

As detailed in **Sec. 4.3**, we used five metrics to evaluate clustering performance. Here, we combined these five metrics by taking their average, which is represented on the y-axis of **Fig 9** as the averaged clustering metrics. From **Fig 9**, it is evident that the reference method of random selection demonstrates the poorest clustering performance across all types of datasets. In the PBMC coarse-resolution dataset, Mcadet does not outperform any FS method except for random selection (NS, one-sided pairwise t-test). However, in the PBMC fine-resolution dataset, Mcadet performs well, ranking in the top three. In the simulated datasets, Mcadet also ranks highly. Unlike in the PBMC datasets, Mcadet performs well in the simulated coarse-resolution datasets. The separate plots of these five metrics are shown in **S7–S11 Figs**, which can provide insights into which method performs better on each specific metric. Similar to **Fig 3**, the trend of mean averaged clustering metrics is shown in **S12 Fig**. From the figure, it is evident that the random selection method, compared to other FS methods, consistently remains at a low level. Additionally, as the number of selected genes increases, the evaluation metric also improves, which makes sense. However, we also observe that except for the PBMC fine-resolution datasets, where Mcadet gradually surpasses all other methods as the number of selected genes increases, Mcadet does not show great differences from other methods in other types of datasets. The results indicate that for clustering in downstream analysis, the advantage of Mcadet is not as pronounced as it is for gene selection quality in **Section 2.1**, particularly in the PBMC datasets, whether coarse-resolution or fine-resolution.

#### 2.2.2 HVGs associated with trajectory inference

Besides clustering, another important step in downstream analysis is trajectory inference. Therefore, we conducted an analysis comparing the impact of Mcadet and other FS methods on trajectory inference. This experiment was inspired by Ranek et al **[26]**. We employed SymSim package **[27]** to simulate scRNA-seq datasets. In **Sec. 4.1**, we introduced the SPARSim package **[28]** for generating simulated datasets. However, in **Sec. 2.2.2**, we switched to SymSim because this package is particularly well-suited for generating continuous scRNA-seq data with developmental trajectories. SymSim allows for inputting various custom types of phylogenetic trees, making it ideal for creating datasets for trajectory inference. We simulated 5 tree differentiation trajectories containing 2,000 cells and 5,000 genes. For each differentiation trajectory, we generated 10 datasets by setting different random seeds. The diagrams of these five types of trees are shown in **S7–S11 Figs**.

**Fig 10:**
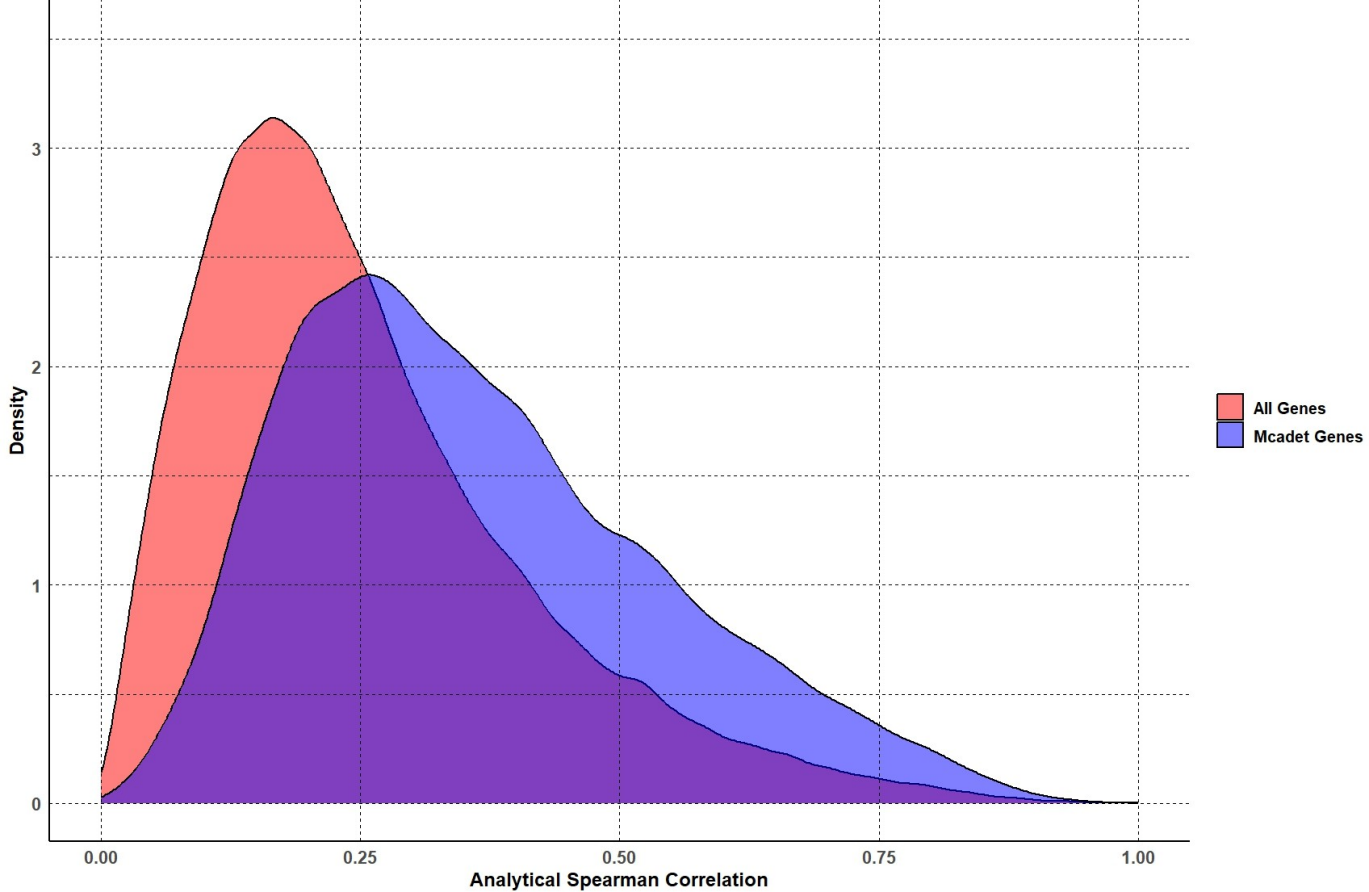
Density plot of the analytical Spearman correlations. Density distribution of the analytical Spearman correlations for genes selected by Mcadet (red) compared to all genes (blue) across 50 generated continuous scRNA-seq datasets.

We applied Mcadet to 50 generated continuous scRNA-seq datasets with 5 different trajectory structures. After identifying the selected genes by Mcadet, we calculated the Spearman correlations between their gene expressions and the pseudotime for each branch. The branch information and corresponding pseudotime, considered as the ground truth, were derived from the SysSim package. For each gene, we determined the maximum absolute value of the Spearman correlations across all branches as the analytical Spearman correlation. We aggregated all selected genes from the 50 generated datasets to obtain the pooled analytical Spearman correlations. **Fig 10** illustrates the density distributions of the analytical Spearman correlations for the genes selected by Mcadet and all genes in the generated datasets. From the figure, we can observe that the density peak of the analytical Spearman correlations for the genes selected by Mcadet shifts to the right. Additionally, the density of genes with analytical Spearman correlations greater than 0.5 is higher for the selected genes compared to all genes. This indicates that Mcadet has the capability to perform feature selection on continuous data with varying trajectory structures.

**Fig 11:**
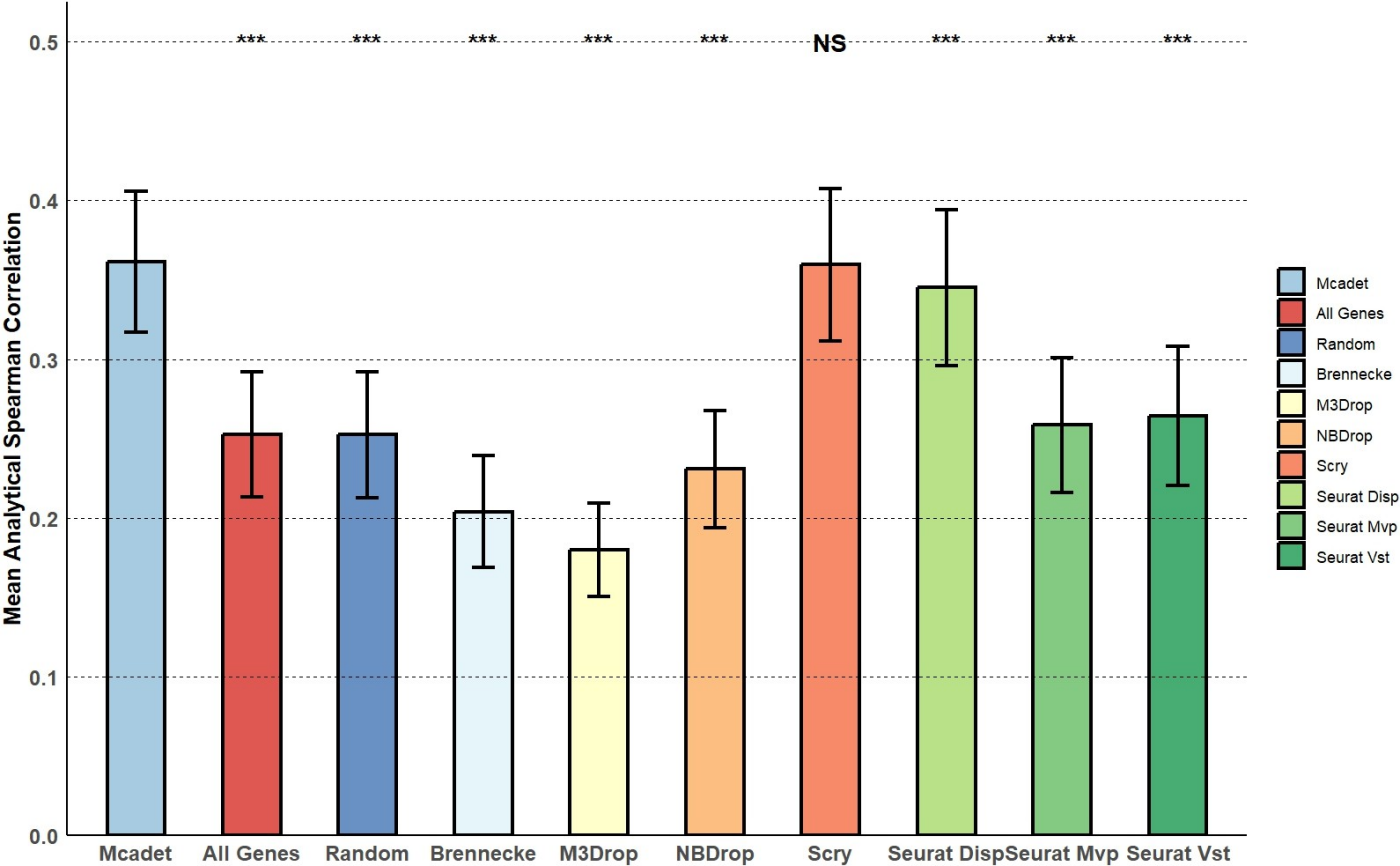
Comparison of feature selection methods using mean analytical Spearman correlations. The p-values, obtained through one-sided pairwise t-tests, indicate the significance of the differences between Mcadet method and each other FS method. Ns: non-significance, NS: p ≥ 0.05,*: 0.01 ≤ p < 0.05, **: 0.001 ≤ p < 0.01, ***: p < 0.001.

Similarly, we applied other FS methods to the 50 generated continuous scRNA-seq datasets. We calculated the analytical Spearman correlation for each selected gene, pooled these correlations across the 50 datasets, and then calculated the mean and standard deviation of the analytical Spearman correlation for each FS method. **Fig 11** shows the bar plot comparing these methods. The second and third bars from the left represent the results for all genes and 1,000 randomly selected genes from the total 5,000 genes, respectively. We can observe that Mcadet performs the best, significantly outperforming all methods except for Scry. The pairwise comparisons were done by one-sided t-tests. This result indicates that Mcadet’s feature selection has the capability to perform feature selection on continuous data with varying trajectory structures and outperforms many other commonly used methods.

#### 2.3. 2-D visualizations, biplot and UMAP

**Fig 12:**
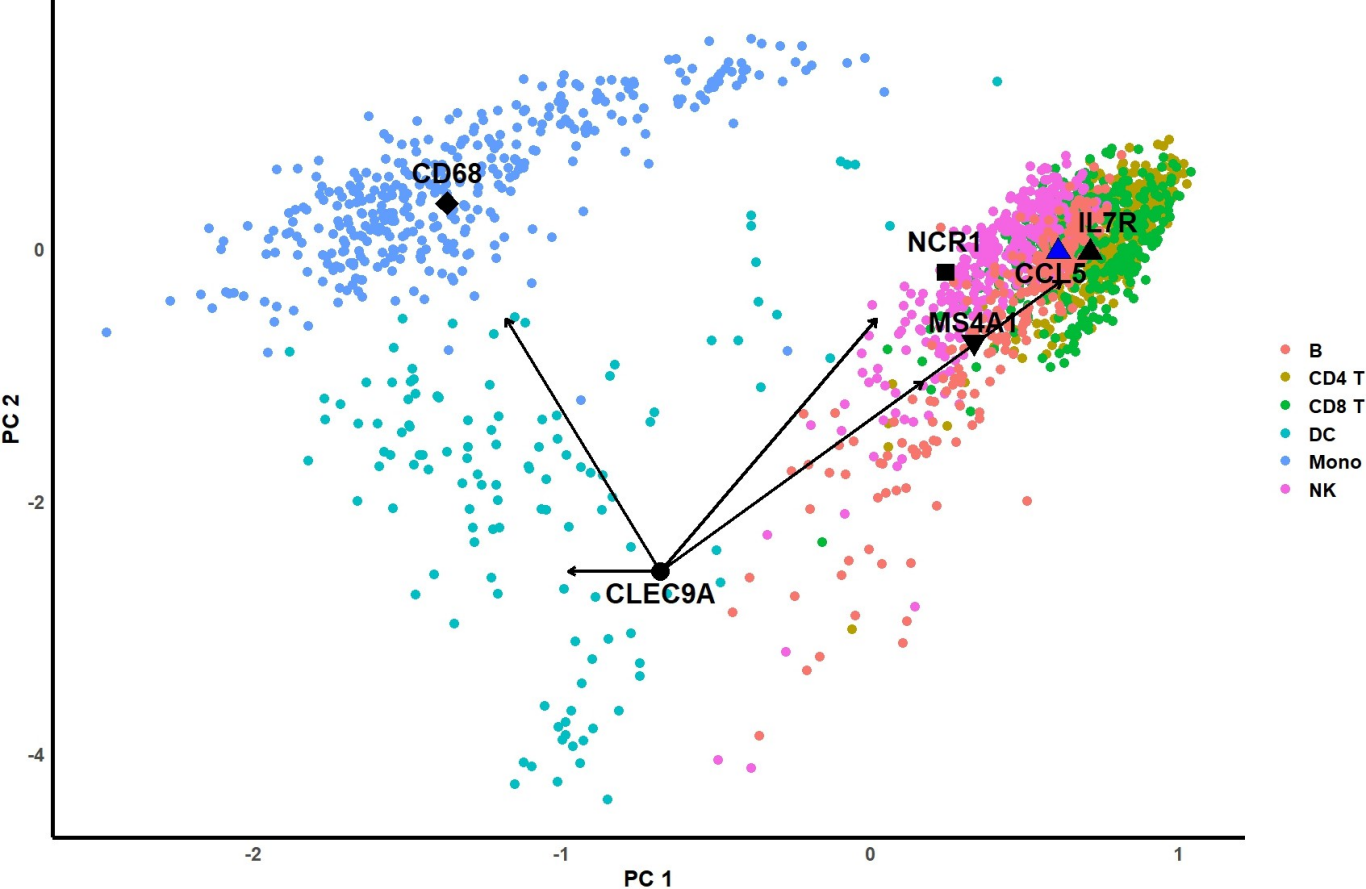
2D biplot of a coarse-resolution PBMC dataset. X-axis and Y-axis are the first two PCs of standard row coordinates of cells (dots) and the principal coordinates of genes (+) (texts). The black arrows represent the Euclidean distance from genes to the cell centroid.

**Fig 13:**
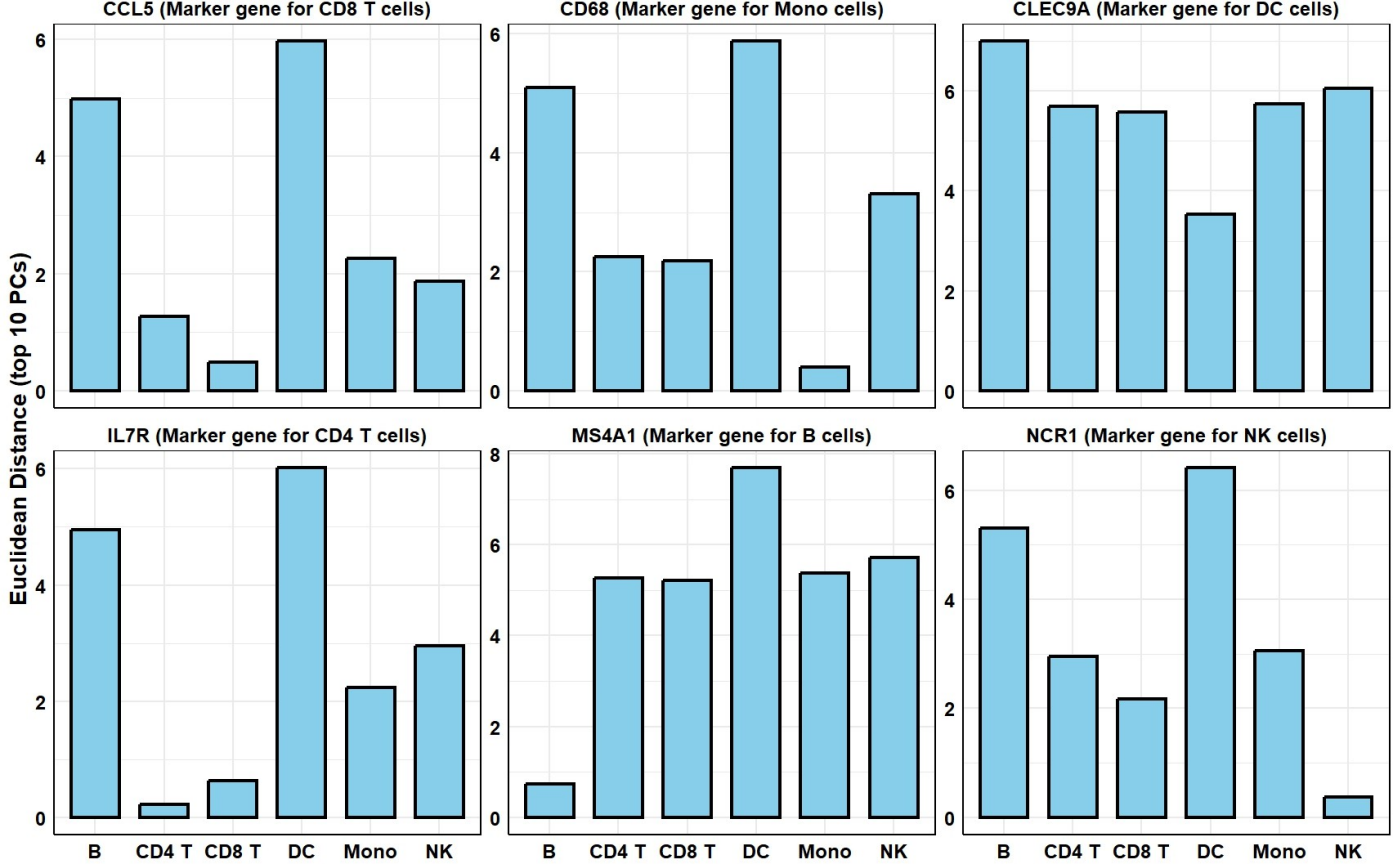
The Euclidean distances between different marker genes to the centroid of each coarse-resolution PBMC cell type. Top 60 PCs were used to calculate the Euclidean distances in the embedded biplot space.

In **Sec. 4.1**, we discussed that a key function of MCA is to simultaneously project row and column variables into the same embedded space, known as biplot space **[29]**. It is important to note that this biplot can be multi-dimensional; the “bi” in biplot refers to both rows and columns, not just two dimensions. However, in the visualizations in this section, we use a 2-D biplot for illustration. **Fig 12** is a 2-D biplot of a coarse-resolution PBMC dataset, generated using standard row coordinates and principal column coordinates after MCA. We illustrated 6 cell types; each dot represents a cell. Different colors represent different cell types. We observed that the clusters of these cell types appear flattened in this 2-D visualization. This is likely because the first 2 PCs may not fully capture the true pattern in the high-dimensional space. However, we can still distinguish the three clusters in this embedded space. It is widely known that the important marker genes for these cell types are MS4A1 for B cells, IL7R for CD4 T cells, CCL5 for CD8 T cells, CD68 for monocytes, NCR1 for NK cells, and CLEC9A for DC cells. These marker genes have been depicted in the biplot using triangular, square, or round shapes filled with black. The black arrows represent the distances between the DC cell marker gene, CLEC9A, and the centroids of all cell clusters. It is evident that this gene is closest to the centroid of the DC cell cluster. Similarly, other marker genes are positioned near their respective cell type clusters. However, since this representation is limited to a two-dimensional space, it may not fully capture the distance information. The bar plot in **Fig 13** below provides a clearer view. We used the top 60 principal components to calculate the Euclidean distances between the cell type centroids and each marker gene. The results show that for each cell type’s marker gene, the shortest distance is to the centroid of the corresponding cell type cluster. Notably, CCL5 and IL7R are close to both CD8 T cell and CD4 T cell clusters, which is expected given the similarity between these T cell subtypes.

**Fig 14:**
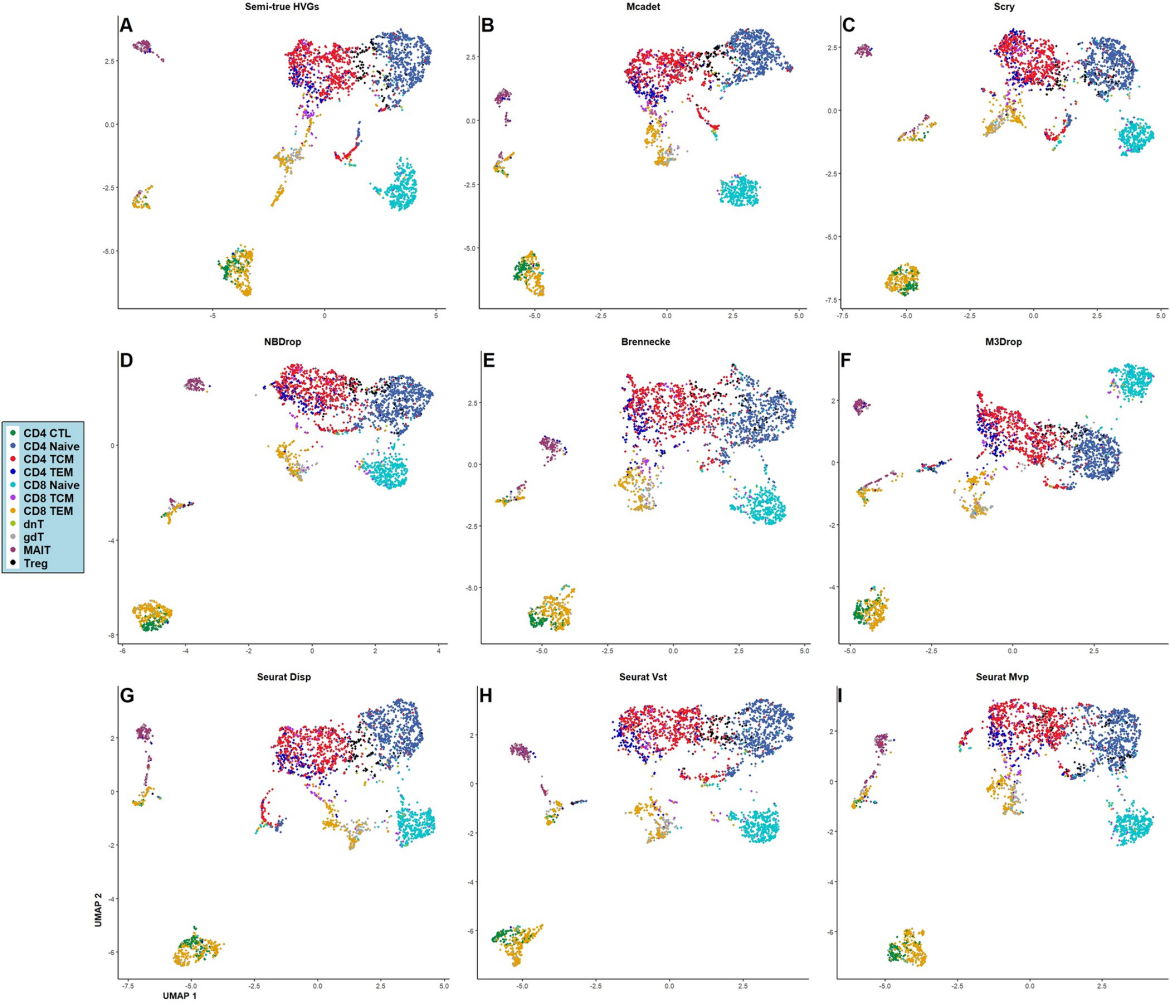
UMAP visualization of a fine-resolution PBMC dataset with true annotated labels by different FS methods. A-G: HVGs of semi-ground truth; HVGs selected by Mcadet, HVGs selected by Scry, HVGs selected by NBDrop, HVGs selected by Brennecke, HVGs selected by M3Drop, HVGs selected by Seurat Disp, HVGs selected by Seurat Vst, HVGs selected by Seurat Mvp.

**Fig 15:**
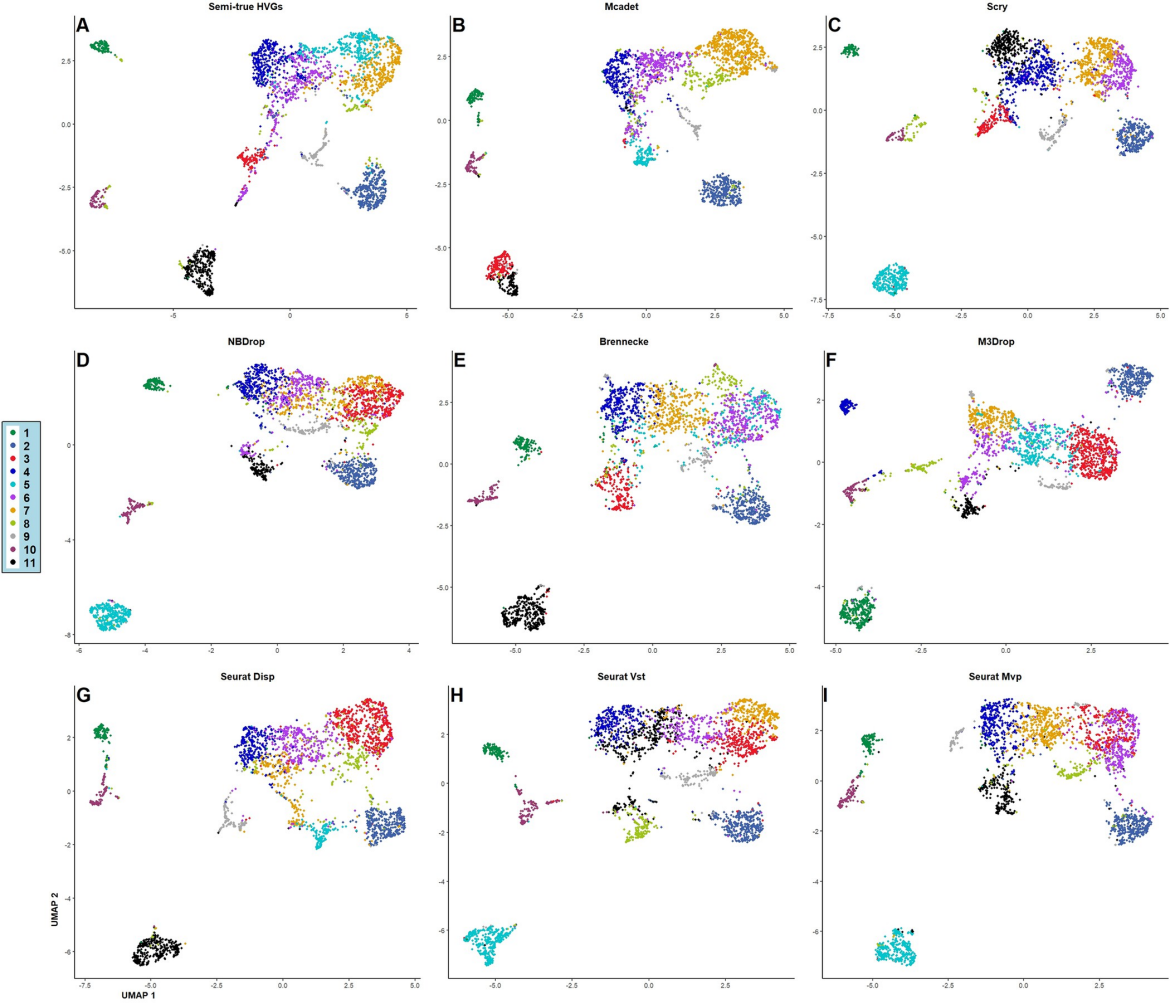
UMAP visualizations of the same fine-resolution PBMC dataset with Fig 14, colored by *k*-means clustering labels by different FS methods. A-G: HVGs of semi-ground truth; HVGs selected by Mcadet, HVGs selected by Scry, HVGs selected by NBDrop, HVGs selected by Brennecke, HVGs selected by M3Drop, HVGs selected by Seurat Disp, HVGs selected by Seurat Vst, HVGs selected by Seurat Mvp.

In addition to using 2-D biplot visualization to demonstrate the core FS process of Mcadet, we also employed 2-D UMAP (Uniform Manifold Approximation and Projection) **[30]** visualization to subjectively show the differentiation capabilities of various FS methods on different cell types with true labels, as well as the distinction between different cell labels after downstream clustering analysis. **Fig 14** shows the UMAP visualization of a fine-resolution PBMC dataset with true labels and clustering labels by different FS methods. **Fig 15** shows UMAP visualizations of the same fine-resolution PBMC dataset with **Fig 14**, colored by *k*-means clustering labels by different FS methods. Specifically, we performed PCA to retain the top 15 PCs, followed by *k*-means clustering, as we know that PBMC fine-resolution contains 11 cell types. Different labels are then visualized with different colors, noting that the colors representing categories 1 to 11 are just *k*-means clustering labels applied after the FS method, without biological meaning.

**Fig 14A** shows that the UMAP visualization of semi-true HVGs can generally distinguish different PBMC fine-resolution cell populations. However, we observe that the cluster at the bottom of **Fig 14A** is a mixture of CD4 CTL and CD8 TEM cells. When clustered as shown in **Fig 15A**, they are grouped as type 11 in black, which could mislead downstream analyses. In contrast, **Figs 14B** and **15B**, which use Mcadet for FS, show that the mixed cluster of CD4 CTL and CD8 TEM cells is separated by *k*-means clustering, highlighting an improvement in downstream clustering due to Mcadet’s feature selection. From **Fig 14A–14I**, We can see that the visual differences between different FS methods are minimal. In **Fig 15**, we observe some differences. As claimed above, only Mcadet (**Fig 15B**) distinguishes the mixture of CD4 CTL and CD8 TEM cells in clustering, a unique capability for PBMC fine-resolution downstream clustering not achieved by any other FS methods. However, it is noteworthy that the UMAP visualization in **Fig 14B** does not display a significantly greater separation between these cell types. This suggests that while Mcadet enhances clustering accuracy, the UMAP projection may not fully capture this improvement due to its dimensionality reduction limitations.

We also included the random selection for comparison as shown in **S18 Fig**, from the figure, randomly selected genes do not effectively represent or retain biological information, neither in terms of true label representation nor downstream clustering labels. Given that UMAP is influenced by random seed, we also input two additional different random seeds. The results, shown in **S19** and **S20 Figs**, are consistent with **S18 Fig**.

## 3. Discussion

FS methods are integral to scRNA-seq analysis pipelines, as they facilitate the acquisition of a reduced-dimensional dataset that captures crucial information, promoting the interpretation and understanding of the underlying biological processes. Therefore, it is crucial to avoid the inclusion of non-informative genes or the neglect of marker genes. In the context of scRNA-seq downstream analysis, the careful selection of a suitable feature set in advance is pivotal, as it directly impacts the quality of the clustering or trajectory inference results. It is essential to acknowledge that individual FS methods operate based on specific assumptions pertaining to the characteristics that determine the relevance of genes. Certain FS methods adopt gene dispersion as a criterion, postulating that the variability in gene expression is indicative of its biological significance. Conversely, other FS methods, such as NBdrop and M3drop, identify genes with a higher-than-expected proportion of zero-counts, derived from a null distribution, as more informative. Moreover, recent advancements have introduced feature selection methods that leverage graph-based and clustering machine learning algorithms, such as FEAST and Triku, for FS in scRNA-seq data analysis. Despite the extensive development and widespread adoption of various FS methods tailored for scRNA-seq data, there is still a need for exploration, investigation, and proposition of new FS methods adapted to address specific challenges. For instance, certain fine resolution datasets or datasets with minority cell populations necessitate specialized FS methods that have yet to be thoroughly explored or developed. Further research in this area holds promise for enhancing scRNA-seq analysis and uncovering novel insights into cellular heterogeneity and biology.

In this study, we present Mcadet, a novel feature selection method designed specifically for scRNA-seq data. Mcadet leverages MCA and community detection techniques to address the challenges posed by fine-resolution datasets and datasets containing minority cell populations. Using a diverse collection of datasets comprising both simulated and real-world PBMC datasets, we evaluated the performance of Mcadet in identifying highly informative genes. Our assessment considered various evaluation metrics for the quality of the selected genes and for the impact of downstream analysis. Notably, Mcadet demonstrated excellent performance on datasets in fine resolution in terms of the quality of the selected genes, which are often challenging to differentiate. It also exhibited outstanding ability in detecting HVGs in the datasets associated with continuous cell populations or minority cell populations. Furthermore, Mcadet successfully determined an appropriate number of HVGs, and in scenarios involving artificially reduced gene expression, our method performed well in identifying genes that exhibit both low expression levels and high variability. Particularly Mcadet demonstrated a balanced approach in selecting genes across a wide range of expression levels, the density resembles that of semi-true HVGs of fine-resolution PBMC datasets, without exhibiting any bias or preference. However, for clustering in downstream analysis, the evaluation metrics indicate that Mcadet, compared to other methods, is not exceptionally outstanding, typically ranking around the top four. While Mcadet does not outperform all competing methods on every dataset, its advantage lies in its overall accuracy, especially on fine-resolution data. Although Scry demonstrates high accuracy in feature selection, it performs less effectively than Mcadet in downstream tasks such as clustering and trajectory inference. Therefore, Mcadet provides a balanced performance.

Our method comprises five major steps: (1). Matrix pre-processing. (2). MCA decomposition. (3). Community detection for cell clustering. (4). Computation and ranking of Euclidean distances. (5). Statistical testing. The efficacy of Mcadet in identifying highly informative genes is primarily attributed to Step (2), (3) and (5), where the MCA decomposition isolates intrinsic variation, which carries greater biological significance, the application of community detection with varied resolution parameters facilitates robust cell population clustering, and a novel statistical testing approach is proposed to select a relatively suitable number of HVGs. Consequently, Mcadet exhibits exceptional performance in fine-resolution datasets. This flexibility enables the accurate detection of fine-resolution patterns and minority cell populations within the data, enhancing the analytical capabilities of Mcadet in challenging scRNA-seq scenarios.

CA is a multivariate statistical technique primarily used to explore relationships within categorical data. It is particularly effective for visualizing the associations between rows and columns in a contingency table. The input is a contingency table, where the rows represent categories of one variable and the columns represent categories of another variable. CA calculates the row and column masses, which are the relative frequencies of each category, transforming the table into a correspondence matrix. This scaling ensures that both rows and columns are on the same dimensional plane, making the subsequent standard row and principal column coordinates directly comparable. Unlike other dimensionality reduction techniques, such as PCA, which typically provide projections for either genes or cells, CA integrates both aspects within the same framework. The final step in CA involves performing SVD on the standardized residuals (the difference between observed and expected frequencies). This decomposition yields the standard row and principal column coordinates, which can then be used for further calculations or visualizations, offering a comprehensive view of the data’s underlying structure. MCA extends CA to handle more than two categorical variables, making it a powerful tool for multivariate categorical data analysis. Comparing to CA, the input of MCA is a data matrix where each row represents an observation, and each column represents a categorical variable, then the categorical data matrix is transformed to a binary indicator matrix, and the following steps are the same as CA. It is widely utilized across various fields: To analyze and visualize responses from surveys with multiple categorical questions in survey analysis; To explore complex relationships between different social and psychological factors in sociology and psychology. This versatility makes MCA an essential technique for uncovering patterns and relationships in complex categorical datasets.

Based on the above description, if we want to apply CA or MCA to a scRNA-seq data matrix, we will use MCA. In this context, each row, representing a cell, corresponds to an observation, and each column, representing a gene, corresponds to a categorical variable, making MCA more appropriate. The Cell-ID paper does not explicitly mention the reasons for using MCA instead of CA, this potential rationale aligns with the need to consider genes as separate variables and cells as distinct observations, leading to the adoption of MCA in our methodology.

Since each gene is treated as a categorical variable, we need to define categories. We use fuzzy coding to transform the data matrix. Fuzzy coding converts a variable into two or more categorical variables. It is important to note that this expansion of data into a higher-dimensional space can result in longer computation times for matrix decomposition. For example, if each gene is converted into four categories, a sample with 15,000 genes would expand to 60,000 gene categories. This significant increase in the number of categories presents challenges in terms of computation efficiency. Therefore, we adopt the “doubling” technique in correspondence analysis **[31,32]**. In this approach, each gene is divided into two categories: one representing the presence of the gene *g*_+_, and the other representing the absence of the gene *g*_−_. Fuzzy coding is advantageous because it allows us to capture the variability and nuances in gene expression more effectively. By representing each gene as both present and absent, we can better model the partial expression levels that are common in scRNA-seq data. This approach enhances the sensitivity of our analysis, providing a more detailed and accurate representation of the underlying biological processes. We also conducted a comparison by leaving out the pre-processing step. In other words, we examined the results when we bypassed fuzzy coding and directly applied MCA. **S21 Fig** presents the results of this comparison. From the figure, we observe that except for the fine-resolution simulated datasets, the performance with preprocessing is superior to that without preprocessing.

In step (4) of our approach, the calculation of Euclidean distance involves projecting all genes (*g*_+_), and cells onto the same space, referred to as a biplot. Biplots are a type of exploratory graph widely used in statistics. They represent the rows and columns of a data matrix as points or vectors in a low-dimensional Euclidean space. While biplots are typically visualized in two or three dimensions for ease of interpretation, they can be extended to higher-dimensional spaces for computational or analytical purposes. In this low-dimensional space, the proximity, as measured by Euclidean distance, between a column (e.g., gene) and a row (e.g., cell) indicates the degree of specificity of the gene to the cell. For example, if a gene *j* exhibits extreme proximity to cell cluster *A* (i.e., ranking first in distances between all genes and centroid *A*), but is distant from cluster *B*. (i.e., ranking last in distances between all genes and centroid *B*), gene *j* can be considered a candidate HVG that provides informative discriminatory power for distinguishing different cell types or cell states.

In our approach, the task of cell clustering is performed in an unsupervised manner, aiming to discover latent cell types or states within the dataset. The motivation for clustering and then computing distances between cell cluster centroids and genes, rather than directly calculating pairwise distances between each gene and cell, lies in the high computational cost. Given the large number of genes and cells in single-cell sequencing, computing pairwise distances between each gene and individual cell would be computationally cumbersome. Clustering cells into distinct types, where within-type similarity is high, significantly reduces the number of distance calculations while maintaining biological relevance. While various existing clustering algorithms, such as *k*-means, DBSCAN, and Gaussian mixture models, can be utilized, they often face limitations when applied to high-dimensional and sparse scRNA-seq data. For instance, *k*-means requires the number of clusters to be pre-specified, which is challenging for unknown samples. As a result, we turn to graph-based community detection algorithms. One drawback of community detection algorithms is their dependence on the resolution parameter, which influences the clustering outcome. Different resolution values can lead to distinct cluster assignments, making the partitioning result less robust. However, in our context, it is not necessary to precisely determine the exact number of cell types present in the sample. This is due to several reasons: (1). After clustering, the selection of features is based on a novel metric, the log-ratio between the maximum and minimum rank, which is not sensitive to the performance of a specific clustering algorithm. (2). The clustering step in our feature selection workflow aims to enhance the distinguishability of distances between genes and cells, simplifying the identification of informative features. It is not intended to determine the exact number of cell types present, as additional clustering techniques can be applied after the feature selection process. (3). To obtain a more robust clustering result, we consider a range of resolution values and evaluate the overall test statistic. (4). The true partitioning of cell populations can be dynamic, exhibiting variations in fine and coarse resolutions. Therefore, we employ the Leiden algorithm in our feature selection workflow to address this aspect.

However, it is essential to acknowledge the limitations of Mcadet. One limitation is the running time of the method, as demonstrated in **S22 Fig**. The experiments were conducted on a desktop with an AMD Ryzen 9 7950X 16-Core Processor and 64 GB RAM. It was observed that the running time of Mcadet increases linearly with the number of genes or cells in the dataset. In comparison to other feature selection methods, Mcadet exhibits significantly longer running times. This extended duration can be attributed to the inclusion of computationally intensive machine learning steps within our framework, which are absent in other compared feature selection methods. The second limitation of our approach is the current reliance on the Leiden algorithm for cell clustering. While it is currently the optimal choice for our proposed framework, advancements in clustering or network analysis may lead to the emergence of new and powerful algorithms that could replace Leiden in the future. A third limitation is that although we used a number of datasets, including 48 real-world datasets, all of them are PBMC datasets. In future work, it would be beneficial to include datasets from other cell types or biological systems to further validate the performance and generalizability of Mcadet. Furthermore, it is important to note that we did not consider all available feature selection methods, such as recently published machine learning-based approaches. Including these methods in comparative evaluations would provide a more comprehensive analysis of Mcadet’s performance and its position among other state-of-the-art techniques. Fourth, we acknowledge that we did not extensively explore optimal parameter settings for Mcadet and instead relied on default parameter configurations. Future investigations should consider conducting parameter optimization to further refine the performance of the method and explore its sensitivity to different parameter choices. Finally, all the data in this study are split both by donor and batch, so the technical biases are not present in our data modality. In the future, we will introduce integrated scRNA-seq datasets to further validate our method.

## 4. Materials and methods

### 4.1 Materials and datasets

Coarse resolution scRNA-seq data implies that the differences between various cell populations are significant. These differences are easily distinguishable in terms of biological functions and specific gene expressions. In the PBMC dataset, we can represent coarse resolution with a broad classification, such as B cells, CD4 T cells, and CD8 T cells. In contrast, fine-resolution scRNA-seq data means that the differences between different cell populations are minimal and thus difficult to distinguish. This can be represented in the PBMC data with a more detailed classification, such as CD4 Central Memory T Cells (TCM) and CD4 Effector Memory T Cells (TEM). When simulating scRNA-seq data, we can directly tune the differentially expressed (DE) parameters to either increase or decrease the differences between cell populations, thereby generating coarse- or fine-resolution datasets.

To perform the simulation study, we generated 48 simulated datasets using the SPARsim package [28] in R. SPARsim utilizes a Gamma-Multivariate hypergeometric probabilistic model to create count matrices that closely resemble the distribution of zeros observed in real count data. In a recent evaluation of 16 scRNA-seq simulation methods by Crowell et al. **[33]**, SPARsim produced results that were most similar to real data, followed by Splatter, which is a widely used simulator for scRNA-seq data. Furthermore, SPARsim was highly recommended in a benchmark study by Cao et al. **[34]** for systematic evaluation of simulation methods for scRNA-seq data. These 48 datasets are divided into two groups: 24 fine-resolution and 24 coarse-resolution datasets. Each dataset consists of 6,000 cells and 15,000 genes, with a total of 10 cell populations. The abundances of these cell populations are as follows: 25%, 20%, 16%, 10%, 8%, 7%, 6%, 4%, 3%, and 1% of the total cells.

To set up the initial parameters, we estimated them using the 10X Genomics example datasets from Zheng et al. **[35]**, specifically the human Jurkat and 293T cells, which are also included as built-in datasets in the SPARsim package. Based on the estimated parameters from 1,718 293T cells, we created 10 different cell groups with the respective abundances mentioned above. In each cell group, we designated 20 genes out of the 15,000 genes as driver genes, which were only upregulated in one type of cell. Therefore, there were a total of 200 driver genes (20 genes per cell group). Additionally, we selected another 1,800 genes to be DE genes, which were either upregulated or downregulated in two or more cell types. This resulted in a total of 2,000 informative genes.

The SPARsim package uses fold change multiplier to control the magnitude of differential expression. A fold change multiplier of 1 indicates no differential expression, while values greater than 1 represent upregulation and values less than 1 represent downregulation. For the upregulated and downregulated DE genes, we generated the fold change multipliers from uniform distributions: Unif (*a*_*up*_, *b*_*up*_) and Unif (*a*_*down*_, *b*_*down*_) respectively.

For the fine-resolution datasets, we defined 24 levels of intensities by selecting the parameters, *a*_*up*_ ∈ (1.5, 2), *b*_*up*_ ∈ (2, 2.5), *a*_*down*_ ∈ (0.4, 0.6), *b*_*down*_ ∈ (0.6, 0.8). These four parameters were chosen 24 times in equally spaced intervals within the specified ranges. Similarly, for the coarse-resolution datasets, we set up 24 levels of intensities by selecting parameters *a*_*up*_ ∈ (2, 3), *b*_*up*_ ∈ (2.5, 3.5), *a*_*down*_ ∈ (0.1, 0.3), and *b*_*down*_ ∈ (0.3, 0.5). Each dataset in the fine- or coarse-resolution group corresponds to a specific DE fold change multiplier.

Regarding the driver genes, the parameters in the uniform distributions Unif (*a*_*up*_, *b*_*up*_) were multiplied by 1.2. As the value of *a*_*up*_ increases and *a*_*down*_ decreases, the differential expression of the genes becomes more pronounced between cell groups, making these genes easier to identify using feature selection methods. Therefore, we considered the 24 datasets with more extreme differences between cell groups as fine-resolution simulated datasets, while the other 24 datasets were coarse-resolution simulated datasets.

In our study, we utilized scRNA-seq datasets provided by 10X Genomics, specifically the datasets of PBMCs from the recently published CITE-seq reference dataset by Hao et al. **[36]**. It included 24 human PBMC datasets, which represented 8 different donors and 3 different batch times. These datasets were manually annotated at three different resolutions: fine, moderate, and coarse annotation. The fine annotation contained 56 cell types, but due to some cell types having zero cells, we decided to focus on the moderate and coarse annotation for our working datasets. The 24 datasets with coarse annotation labels were considered our real-world coarse-resolution datasets. These datasets consisted of 8 different cell types: B cells, CD4 T cells, CD8 T cells, dendritic cells (DCs), monocytes (Mono), natural killer cells (NK cells), Other cells, and Other T cells. For our fine-resolution datasets, we used the moderate resolution labels but restricted them to T cells. This resulted in 24 fine-resolution datasets containing 11 T cell types: CD4 cytotoxic T lymphocytes (CTLs), CD4 naïve T cells, CD4 central memory T cells (TCMs), CD4 effector memory T cells (TEMs), CD8 naïve T cells, CD8 TCMs, CD8 TEMs, double-negative T cells (dnT), gamma-delta T cells (gdT), mucosal-associated invariant T cells (MAIT), and regulatory T cells (Tregs). To summarize, we have a total of 96 datasets: 24 datasets each for simulation coarse-resolution datasets, simulation fine-resolution datasets, PBMC coarse-resolution datasets, and PBMC fine-resolution datasets.

To determine the ground truth informative gene list for the simulated datasets, we explicitly specified which genes were considered informative, totaling 2,000 genes. Obtaining the ground truth informative gene list for the PBMC datasets, however, was more challenging since we did not have that information available. To address this, we utilized the FindAllMarks() function from the Seurat package to identify HVGs for each annotated cell type in the PBMC datasets. This approach allowed us to obtain a semi-ground truth for the PBMC datasets. We set the parameters "return.thresh = 1" and "logfc.threshold = 0" in the FindAllMarks() function to collect all HVGs. Within each cell type, we ordered the genes based on their adjusted p-values in ascending order. If there were fewer than 400 genes with adjusted p-values less than 0.05, we included all of them. However, if there were more than 400 such genes, we selected the top 400 genes based on their adjusted p-values. These collected genes were combined to create a true informative gene list. It should be noted that there might be duplicate genes in this list, specifically when a gene had an adjusted p-value less than 0.05 and was selected in more than two cell types. On average, each PBMC dataset yielded approximately 1,500–2,000 informative genes using this approach. The designation of highly variable genes (HVGs) for the simulated datasets is provided in **S2 Table**. Additionally, the summary information for the PBMC datasets, including both coarse- and fine-resolution datasets, can be found in **S3 Table**.

### 4.2 Methods (Mcadet Workflow)

#### 4.2.1 Matrix pre-processing

For the sake of convenience, we transpose the expression matrix, but this operation does not affect any calculations or interpretations. Let ***Y***_*n*×*p*_ in Eq 1 be the expression matrix of scRNA-seq data, where *n* represents the number of cells (rows) and *p* represents the number of genes (columns). *Y*_*ij*_ denotes the observed expression of the *j*^*th*^ gene in the *i*^*th*^ cell.


$$Y_{n\times p}={\left[\begin{array}{ccccc} Y_{11} & \ldots & \ldots & \ldots & Y_{1p}\\ \ldots & \ldots & \ldots & \ldots & \ldots \\ \ldots & \ldots & Y_{ij} & \ldots & \ldots \\ \ldots & \ldots & \ldots & \ldots & \ldots \\ Y_{n1} & \ldots & \ldots & \ldots & Y_{np} \end{array}\right]}_{n\times p}$$ (1)

In many scRNA-seq data analyses, it is common to apply a log-normal transformation to the raw count matrix and then perform PCA for dimensionality reduction. However, the rationale behind using log counts has limited theoretical justification, and in certain cases, it may obscure meaningful variation. Townes et al. **[15]** suggested that the zero-inflation resulting from log normalization can be problematic as it artificially amplifies the differences between zero and non-zero values. Booeshaghi et al. **[37]** demonstrated that for genes with low expression levels, the average log-transformed expression can significantly differ from the expected value *log*(*λ*), where *λ* is the parameter of the Poisson random variable *X* being modeled, thus leading to potentially misleading interpretations. Furthermore, Hsu et al. **[8]** showed that CA remains robust when applied to either count or log-count data, eliminating the need for log-transformation and its associated issues. Therefore, in our method, we retain the raw count matrix for downstream processing. The fuzzy coding system comprises membership functions and hinge points. By default, the membership function utilized is a piecewise linear (triangular membership function) **[38]**. In our approach, we employ the “doubling” technique in CA. This involves separating each variable into two categories. Consequently, the two hinge points serve as endpoints. For the *j*^*th*^ gene, *m*_1*j*_ (minimum) and *m*_2*j*_ (maximum) are used as the hinge points. The membership functions for the “positive” and “negative” doubled variables are simply defined as follows in Eq 2:

$$Z_{ij+}=\frac{Y_{ij}-m_{1j}}{m_{2j}-m_{1j}}\in [0,1],\;Z_{ij-}=\frac{m_{2j}-Y_{ij}}{m_{2j}-m_{1j}}=1-Z_{ij+}\in [0,1]$$ (2)

Then after fuzzy coding, the original raw count matrix becomes:

$$Z_{n\times 2p}={\left[\ldots \ldots \ldots \ldots \ldots Z_{ij+},Z_{ij-}\ldots \ldots \ldots \ldots \ldots \right]}_{n\times 2p}$$ (3)

where *Z*_*ij*+_+*Z*_*ij*−_ = 1, so the total sum is $\sum_{i=1}^{N}\sum_{j=1}^{P}Z_{ij+}+\sum_{i=1}^{N}\sum_{j=1}^{P}Z_{ij-}=np$. Fuzzy coding can be regarded as a form of linear transformation that restricts values between 0 and 1, similar to the stabilizing effect of log-normalization. However, one distinctive aspect of fuzzy coding in MCA is that it treats genes with high mean expression and genes with low mean expression similarly, if their presence patterns are comparable. Consequently, both genes contribute approximately equally to the biplot plot. This characteristic sets fuzzy coding MCA apart from other transformations.

#### 4.2.2. MCA decomposition

Then the correspondence fuzzy coded matrix can be constructed in Eq (4):

$$P_{n\times 2p}=\frac{1}{np}Z_{n\times 2p}={\left[\begin{array}{cccccccc} P_{11+} & P_{11-} & \ldots & \ldots & \ldots & \ldots & P_{1p+} & P_{1p-}\\ \ldots & \ldots & \ldots & \ldots & \ldots & \ldots & \ldots & \ldots \\ \ldots & \ldots & \ldots & P_{ij+} & P_{ij-} & \ldots & \ldots & \ldots \\ \ldots & \ldots & \ldots & \ldots & \ldots & \ldots & \ldots & \ldots \\ P_{n1+} & P_{n1-} & \ldots & \ldots & \ldots & \ldots & P_{np+} & P_{np-} \end{array}\right]}_{n\times 2p}$$ (4)

The row mass (margin) and column mass (margin) can be computed in Eqs 5 and 6:

$$\mathrm{Row}\;\mathrm{mass}\;r_{i}=\sum_{j=1}^{p}P_{ij+}+\sum_{j=1}^{p}P_{ij-}=\frac{p}{np}=\frac{1}{n}$$ (5)

$$\mathrm{Column}\;\mathrm{mass}\;c_{j+}=\sum_{i=1}^{n}P_{ij+},c_{j-}=\sum_{i=1}^{n}P_{ij-}$$ (6)

In standard MCA method using indicator (or “pseudo indicator”) matrix, the next step is to calculate the standardized Pearson residual matrix with each element:

$$r_{ij+/-}=\frac{observed-expected}{\sqrt{expected}}=\frac{P_{ij+/-}-r_{i}c_{j+/-}}{\sqrt{r_{i}c_{j+/-}}}$$ (7)

In matrix notation, we have:

$$D_{r}^{-\frac{1}{2}}(P-rc^{T})D_{c}^{-\frac{1}{2}}=R_{n\times 2p}={\left[\ldots \ldots \ldots \ldots \ldots r_{ij+},r_{ij-}\ldots \ldots \ldots \ldots \ldots \right]}_{n\times 2p}$$ (8)

where ***r*** is the vector of row mass, and ***c*** is the vector of column mass. ***D***_***r***_ is an *n*×*n* diagonal matrix of row mass, and ***D***_***c***_ is a 2*p*×2*p* diagonal matrix of column mass. Each element *r*_*ij*+_ or *r*_*ij*−_ is defined by standardized Pearson residual mentioned above. Next step is to decompose ***R***_*n*×2*p*_ by using singular value decomposition (SVD) to find left singular matrix ***U***, diagonal matrix of singular values ***D***_***α***_ and right singular matrix ***V*** such that:

$$R_{n\times 2p}=UD_{\alpha }V^{T},\;U^{T}U=VV^{T}=I$$ (9)

In terms of computation method of matrix decomposition, the traditional SVD requires long computation time for large matrix like data matrix in scRNA-seq. Therefore, a fast and memory efficient methods of SVD is used, here we employ irlba **[39]** package in R **[40]**.

After SVD of ***R***_***n***×**2*p***_, *K* = 60 (default is 60, one can tune this parameter as well) components are selected as low-dimensional representation. After rearrangements:

$$P-rc^{T}\approx \sum_{k=1}^{K}\lambda_{k}(D_{r}^{\frac{1}{2}}u_{k}){\left(D_{c}^{\frac{1}{2}}v_{k}\right)}^{T}=\sum_{k=1}^{K}\lambda_{k}(D_{r}D_{r}^{-\frac{1}{2}}u_{k}){\left(D_{c}D_{c}^{-\frac{1}{2}}v_{k}\right)}^{T}$$

$$=D_{r}\left[\sum_{k=1}^{K}\lambda_{k}(D_{r}^{-\frac{1}{2}}u_{k}){\left(D_{c}^{-\frac{1}{2}}v_{k}\right)}^{T}\right]D_{c}$$ (10)

Denote standard coordinates of rows and columns as:

$$\Phi_{\left[K\right]}=D_{r}^{-\frac{1}{2}}U_{\left[K\right]}\;\mathrm{and}\;\Gamma_{\left[K\right]}=D_{c}^{-\frac{1}{2}}V_{\left[K\right]}$$ (11)

Denote principal coordinates of rows and columns as:

$$F_{\left[K\right]}=D_{r}^{-\frac{1}{2}}U_{\left[K\right]}\Lambda_{\left[K\right]}\;\mathrm{and}\;G_{\left[K\right]}=D_{c}^{-\frac{1}{2}}V_{\left[K\right]}\Lambda_{\left[K\right]}$$ (12)

The reconstitution formula is given by:

$$P\approx D_{r}(1_{n}1_{2p}^{T}+\Phi_{\left[K\right]}\Lambda_{\left[K\right]}\Gamma_{\left[K\right]}^{T})D_{c}$$ (13)

Written in column-principal form:

$$P\approx D_{r}(1_{n}1_{2p}^{T}+\Phi_{\left[K\right]}G_{\left[K\right]}^{T})D_{c}$$ (14)

An asymmetric map refers to a situation where the row and column points are scaled differently, such as row points being in standard coordinates and column points being in principal coordinates. In contrast, a symmetric map scales the row and column points equally, but it lacks a distance interpretation compared to the asymmetric map **[10]**. Biplots, which are asymmetric CA maps, combine one set of points in principal coordinates and the other set in standard coordinates. In this study, we utilize a column-principal biplot, which involves using standard row coordinates $\Phi_{\left[K\right]}={\left[\phi_{1},\phi_{2},\cdots ,\phi_{n}\right]}^{T}$ and principal column coordinates $G_{\left[K\right]}={\left[g_{1+},g_{1-},\cdots ,g_{p+},g_{p-}\right]}^{T}$ for downstream calculations. Here, ***ϕ***_***i***_ represents a *K*×1 vector of standard row coordinates for cell *i*, and ***g***_***j***+/−_ represents a *K*×1 vector of principal column coordinates for gene *j*. ***g***_***j***+_ denotes the presence of the gene, while ***g***_***j***−_ indicates its absence. Each set of two categories for each gene is centered at the origin, that is, ${\vec{g}}_{j+}+{\vec{g}}_{j-}=\vec{0}$ **[7,38,41]**. Consequently, only the gene category coordinates ${\vec{g}}_{j+}$ (principal column coordinates), which convey the presence of gene expression relative to the maximum per gene, i.e., $G_{\left[K\right]}^{*}={\left[g_{1+},\cdots ,g_{p+}\right]}^{T}$, are retained for downstream analysis.

#### 4.2.3. Community detection

Clustering or community detection is performed using the Leiden algorithm, a graph-based clustering algorithm that is faster than the Louvain algorithm. We utilize the R package leidenAlg for implementing the Leiden algorithm **[42]**. Our specific weighting of the *k*NN graph for the clustering step was inspired by the methods outlined in PhenoGraph **[43]** and SNN-Cliq **[44]**. In summary, this approach embeds cells within a graph structure, such as a *k*-nearest neighbor (*k*NN) graph, where edges are established between cells exhibiting similar feature expression patterns. The goal is to partition this graph into highly interconnected ’quasi-cliques’ or ’communities.’ Following the methodology of PhenoGraph, we initially construct a *k*NN graph based on Euclidean distance in PCA space and subsequently refine the edge weights between any two cells by evaluating the shared overlap in their local neighborhoods using Jaccard similarity. During the construction of the KNN graph, we set the value of *k* as 1% of the total number of cells in the dataset. To obtain a robust partition, we keep the value of *k* fixed for building the NNgraph and vary the “resolution” parameter in the “find_partition()” function of the leidenAlg package. We start with a resolution of 0.5 and increment it by 0.1, running the algorithm 10 times by default.

#### 4.2.4 Calculating and ranking the Euclidean distance

Having established the cell communities (or clusters), assume there are *d* clusters, a set of cells *Ψ* ∈ {*C*_1_,⋯,*C*_*d*_} can be defined, we can calculate the coordinates of each cluster centroid by taking the average coordinates of the cells in a cluster in Eq 15:

$$T_{\Psi }=\frac{1}{\left|\Psi \right|}\cdot \sum_{i\in \Psi }\phi_{i}$$ (15)

where ***T***_***Ψ***_ is a *K*×1 vector, the coordinates of cell community *Ψ*, |*Ψ*| is the number of cells in community *Ψ*, and ***ϕ***_***i***_ is the coordinates of cell *i* in this low-dimensional space we defined above.

Once we have obtained the coordinates of each cluster centroid in the low-dimensional MCA space, we can calculate the Euclidean distances between each gene (+) and each cell centroid. In this low-dimensional MCA biplot representation, the distance between a column (representing a gene +) and a row (representing a cell) indicates the specificity of that gene to the corresponding cell. A shorter distance suggests a higher specificity. Therefore, to assess the specificity of a gene to a particular cell community, we calculate the Euclidean distance between each gene and each community centroid in Eq 16.

$$d_{\left(j,\Psi \right)}=\sqrt{{\left(g_{j+}-T_{\Psi }\right)}^{T}(g_{j+}-T_{\Psi })}$$ (16)

where *d*_(*j*,*Ψ*)_ is the Euclidean distance between gene *j*(+) and cell community centroid *Ψ*, and ***g***_***j***+_ is the coordinates of gene *j* in this low-dimensional space we defined above. ***T***_***Ψ***_ is the coordinate of cell community *Ψ*. Next, based on the Euclidean distances we have obtained, we can rank them by each community:

$$\left\{rd_{\left(1,\Psi \right)},\cdots ,rd_{\left(p,\Psi \right)}\}=rank\{d_{\left(1,\Psi \right)},\cdots ,d_{\left(p,\Psi \right)}\},\;\Psi \in \{C_{1},\cdots ,C_{d}\right\}$$ (17)

where *rd*_(*j*,*Ψ*)_ is the rank of gene *j* in the Euclidean distances between all genes to cell community *Ψ*’s centroid. We utilize ranks instead of the actual distances because the Euclidean distances can vary significantly in magnitude due to the cells being distributed across an irregular and high-dimensional space. The distances do not follow a normal distribution, making parametric models such as ANOVA less effective. This limitation is also present in the FEAST method, which employs F-statistics to assess feature significance based on mean gene expression **[20]**. However, scRNA-seq data often deviate from normal distributions, even after log normalization, primarily due to the high fraction of zero counts **[15,37]**. Nonetheless, the principle that “the closer a column is to a row, the more specific it is to it” can be applied in any high-dimensional space, regardless of the “curse of dimensionality”, and is not confined to any specific distribution. Thus, the rank of distances for genes within each cluster holds meaningful information. Consequently, we can generate a rank list *Ω*_*j*_ for each gene:

$$\Omega_{j}=\{rd_{\left(j,C_{1}\right)},\cdots ,rd_{\left(j,\Psi \right)},\cdots ,rd_{\left(j,C_{d}\right)}\}$$ (18)

The maximum and minimum order statistics of *Ω*_*j*_, denoted as *max*(*Ω*_*j*_) and *min*(*Ω*_*j*_), provide insights into gene variability. For instance, consider gene *m*_1_ with $min(\Omega_{m_{1}})$ equal to 5, indicating that it ranks significantly higher in a particular cell community compared to other genes. This suggests that gene *m*_1_ is highly expressed in this specific cell community. Conversely, its $max(\Omega_{m_{1}})$ is 10,000, indicating that gene *m*_1_ is not prominently expressed in another population of cells compared to other genes. The log ratio between $max(\Omega_{m_{1}})$ and $min(\Omega_{m_{1}})$, i.e., *log*(10000/5) = 7.6, is relatively large. Thus, gene *m*_1_ can be considered a candidate driver gene in our feature selection task. In contrast, let’s consider gene *m*_2_, where $min(\Omega_{m_{2}})$ is 12,000 and $max(\Omega_{m_{2}})$ is 14,000. These values indicate that gene *m*_2_ is distanced from any cell clusters compared to other genes. The log ratio between $max(\Omega_{m_{2}})$ and $min(\Omega_{m_{2}})$ is only 0.15, suggesting that gene *m*_2_ is unlikely to be selected as a feature. Similarly, a housekeeping gene *m*_3_, which is expressed in the majority of cell types, would be close to any of the cell clusters. In this case, we have $min(\Omega_{m_{3}})=200$ and $max(\Omega_{m_{3}})=800$, resulting in a log ratio of 1.4, which is also small compared to *m*_1_. Therefore, gene *m*_3_ would not be chosen as a feature in our method.

Hence, we define the metric as *logR*:

$$logR_{j}=log\left(\frac{max(\Omega_{j})}{min(\Omega_{j})}\right)$$ (19)

Then we rank *log R*_*j*_ from high to low, and get top *γ* genes specified by users who know how many genes they want:

$$\Gamma =\{g_{j}:|\forall j\in \{1,\cdots ,p\}:\;\underset{j}{rank}(logR_{j},\;\mathrm{descending})\leq \gamma \}$$ (20)

where *Γ* is the final gene set selected. *γ* is the parameter can be tuned by users who have domain knowledge. Descending stands for ranking from high to low.

#### 4.2.5. Statistical testing

The approach described above requires users to specify the number of informative genes to be selected, which relies on domain knowledge and may vary across different samples. However, since the number of genes detected can differ among samples, a more data-driven method is required.

Suppose after Leiden community detection, there are *d* clusters detected, and suppose there are *p* detected genes, and for gene *j*, suppose the rank pattern is: {*X*_1*j*_, ⋯ *X*_*ij*_, ⋯*X*_*dj*_}, *X*_*ij*_ is an integer in 1 to *p*, indicating the Euclidean distance rank for gene *j* in cluster *i*. Our assumption is that for each gene the random variable of rank pattern *X*_*ij*_ is i.i.d. and follows uniform distribution (discrete).


$$P(X_{ij}=k)=\frac{1}{p},1\leq k\leq p$$ (21)

Suppose *X*_(1)_<*X*_(2)_<⋯<*X*_(*d*)_ are the order statistic of random variable *X*. We can derive the joint distribution of minimum *X*_(1)_ and maximum *X*_(*d*)_ as:

$$P(X_{\left(1\right)}=k_{1},X_{\left(d\right)}=k_{2})={\left(\frac{k_{2}-k_{1}-1}{p}\right)}^{d}+{\left(\frac{k_{2}-k_{1}+1}{p}\right)}^{d}-2{\left(\frac{k_{2}-k_{1}}{p}\right)}^{d}$$ (22)

Let $V=logR=log\frac{X_{\left(d\right)}}{X_{\left(1\right)}}$ as the log ratio between the maximum and the minimum rank. The distribution of *V* is,

$$P(V=v)=\sum_{u\in U}[{\left(\frac{ue^{v}-u-1}{p}\right)}^{d}+{\left(\frac{ue^{v}-u+1}{p}\right)}^{d}-2{\left(\frac{ue^{v}-u}{p}\right)}^{d}]$$ (23)

where $U=\{u:ue^{v}\in Z^{+},ue^{v}\leq p\}$.

The proofs of Eqs 22 and 23 are in **S1 Methods**.

This implies that if our assumption (null hypothesis) holds, stating that ranks are chosen with equal probability for each position, the random variable $V=log(\frac{X_{\left(d\right)}}{X_{\left(1\right)}})$ will follow the distribution described in Eq 23 as *Rdis*(*p*, *d*), with two parameters *p* and *d*. Let *Q*(*V* = *v*) = *P*(*V*≤*v*) be the cumulative distribution function defined in Eq 23. For a specific gene, if its observed *V* value is $\tilde{v}$ and $Q(\tilde{v})>1-\alpha$, where *α* is the type I error typically set at 0.05, we can reject the null hypothesis. This suggests that the observed maximum or minimum rank is not randomly selected from the range of 1 to *p*. Instead, it indicates that the gene is more likely to have a higher probability of being assigned the maximum rank among larger numbers or the minimum rank among smaller numbers. This observation implies that the gene may carry informative characteristics.

In real datasets, it is important to acknowledge that the variables *X*_*j*_ are not independent. This is because the presence of one cluster can influence another cluster. While this assumption is strong, it provides a simple model to work with. Furthermore, if we reject the null hypothesis *H*_0_, which is based on the assumption of independent and identically distributed (i.i.d.) variables *X*_*j*_, it suggests that the true distribution of *X*_*j*_ under gene *j* does not follow Eq 21, and therefore, the clusters may not be independent of each other. Additionally, when dealing with a large number of variables *p* (which is typically greater than 10,000 in real datasets), there will be a vast number of possible observed *v* values, given by $v=log(\frac{X_{\left(d\right)}}{X_{\left(1\right)}})$. For instance, if *p* = 15,000, there would be approximately 112,507,500 (about 0.1 billion) possible observations. After removing duplicates, we are left with approximately 68,394,316 (68 million) unique *v* values. Considering this, if we obtain an observation $\tilde{v}$, calculating its p-value using the exact probability mass function from Eq 23 would be time-consuming, as it requires summing up probabilities one by one due to the discrete nature of the distribution. To address this, we employ Monte Carlo simulation to estimate p-values. In each simulation iteration, we randomly draw a rank from 1 to *p* and compute *v* as $log(\frac{X_{\left(d\right)}}{X_{\left(1\right)}})$. This process is repeated *T* times, where *T* is typically set to a large number such as 20,000 or 30,000, in order to generate a simulated distribution. Consequently, p-values can be obtained by comparing the observed $\tilde{v}$ to the simulated distribution. Since each time we input a different resolution parameter for Leiden community detection, we may obtain a different number of clusters *d* from Leiden community detection. When the Leiden algorithm is run *t* times, for time *t* we detect *d*_*t*_ clusters, then, under the null hypothesis, the overall log ratio between maximum rank and minimum rank will follow the distribution:

$$\mathrm{Mixture}\;\mathrm{of}\;\log‐\mathrm{ratio}∼Rdis(p,d_{1})+Rdis(p,d_{2})+\cdots +Rdis(p,d_{t})$$ (24)

Observed log ratios can be calculated by summing all observed data, that is, $\tilde{v}\sim {\tilde{v}}_{1}+{\tilde{v}}_{2}+\cdots +{\tilde{v}}_{t}$. The upper-tailed p-value of each gene is then determined by using the Monte Carlo simulation described above.

#### 4.2.6. Multiple-testing issue

To address the issue of multiple testing, we will utilize the Benjamini-Hochberg (BH) procedure **[45]** to control the false discovery rate. It is important to note that the BH procedure assumes that p-value statistics follow a uniform distribution under the null hypotheses. However, in the case of discrete distributions for the test statistics, this assumption is violated. Our test statistic also follows a discrete distribution. To overcome this limitation, Joshua and Edsel [49s] proposed a stochastic process framework that enables p-value statistics to satisfy the condition of uniformity by introducing a uniform variate for test statistics with discrete distributions. However, in our testing scenario, this correction for p-values has minimal impact. Despite the discrete nature of our test statistic’s distribution, the large number of possible values for the test statistics mentioned earlier results in an approximately continuous distribution. Moreover, scRNA-seq datasets typically contain numerous genes with upper-tailed p-values close to 1, which violates the assumption of uniformity. Therefore, we remove genes with p-values greater than 0.9. Consequently, the remaining p-values under the null hypothesis will be approximately uniformly distributed, allowing us to apply the BH procedure.

### 4.3 Evaluation metrics and other FS methods

In the context of feature selection, the goal is to identify the most relevant features that effectively describe and comprehend the datasets. Specifically, this involves identifying subsets of genes that, when used as inputs in a clustering algorithm, can yield a clustering solution where each cluster represents a potential cell type. To address this, we approached the problem from two perspectives: 1) the accuracy of the selected genes, and 2) the performance of the clustering algorithm.

For assessing the accuracy of the selected genes, we used the Jaccard similarity index as the evaluation metric. The Jaccard similarity index is a statistical measure used to assess the similarity and dissimilarity between two sample sets. It calculates the similarity by dividing the size of the intersection of the sets by the size of their union:

$$J(A,B)\;=\;\frac{\left|A\cap B\right|}{\left|A\cup B\right|}\;=\;\frac{\left|A\cap B\right|}{\left|A|+|B|-|A\cup B\right|}$$ (25)

0≤*J*(*A*, *B*)≤1, where *J*(*A*, *B*) close to 1 indicates a higher similarity between the sets *A* and *B*. In our context, the gene sets selected by different feature selection methods represent set *A*, while the (semi-) ground truth of HVGs for each dataset, as specified in Section 3, represents set *A*. By comparing the Jaccard similarity index, we can evaluate the accuracy of the selected HVGs.

To assess the clustering performance of different feature selection methods, we initially created reduced-dimensional datasets using the selected informative gene sets. Next, we utilized PCA to transform the scRNA-seq data into a lower-dimensional space, specifically reducing the dimensionality to the first 15 principal components (PCs). Subsequently, we applied the *k*-means algorithm to cluster the data within this reduced space. We opted for *k*-means due to its effectiveness in low-dimensional spaces and its suitability when the number of cell clusters in both artificial and real-world datasets is known, allowing for straightforward specification of the value of *k*. After applying the *k*-means algorithm, we obtained the set of clustering denoted as *T*_1_, while the true class is denoted as *T*_2_.

To evaluate the quality of the clustering results, we employed various metrics, including the silhouette score, purity, adjusted Rand index (ARI), normalized mutual information (NMI), and the neighborhood hit. These metrics provide insights into different aspects of clustering performance and help us compare the effectiveness of each feature selection method.

The silhouette value is a measure of how well an object is assigned to its own cluster compared to other clusters **[46]**. It ranges from -1 to 1, with a higher value indicating a better match to its own cluster and a poorer match to neighboring clusters. A higher average silhouette value, known as the silhouette score, indicates that the clustering is more suitable, whereas a lower or negative silhouette score may suggest an insufficient number of clusters or an incorrect clustering configuration. The purity of a cluster is a measure of the proportion of data points belonging to the most frequent class within that cluster **[47]**. It is calculated by summing the number of data points in each cluster that belong to the most common class and dividing it by the total number of data points *N*. The purity can be defined as follows:

$$\frac{1}{N}\sum_{a\in T_{1}}{max}_{b\in T_{2}}|a\cap b|$$ (26)

Rand Index is a measure of similarity between two data clusters in statistics, and in particular in data clustering **[48]**. Normalized Mutual Information is a variation of a measure in information theory known as Mutual Information **[49]**. Mutual information refers to the amount of information about a distribution that can be derived from a second distribution. It is a good measure for determining the quality of clustering. A major reason that it is usually considered is that it has a comprehensive meaning and can be used to compare two partitions, even when there is a difference in number of clusters. Normalized Mutual Information is given by:

$$NMI(T_{2},T_{1})=\frac{2\times I(T_{2};T_{1})}{H(T_{2})+H(T_{1})}$$ (27)

where *H*(∙) is the entropy function, $I(T_{2};T_{1})=H(T_{2})-H(T_{2}|T_{1})$ is the mutual formation between *T*_2_ and *T*_1_.

Neighborhood hit **[50]**, with values ranging from 0 to 1, where 1 indicates the best outcome. In our study, this metric represents the proportion of the *K* neighbors $N_{i}^{\left(K\right)}$ of a point *i* in the embedded low-dimensional space (constructed using the top 15 PCs from MCA and calculated with Euclidean distance) that share the same label l as point i. This proportion is averaged over all points in the embedded space. It measures how well the labeled data is separable in this projection, which helps in evaluating whether the feature selection process can identify informative genes, thus enhancing the distinguishable spatial structure of the embedded space. We conducted the analysis using a value of *K* = 30 as the number of neighbors for calculating the neighborhood hit.


$$\sum_{i=1}^{N}\frac{\left|j\in N_{i}^{\left(K\right)}:l_{j}=l_{i}\right|}{KN}$$ (28)

As mentioned in **Sec. 1.3**, we compared Mcadet with 7 existing feature selection methods. In addition, we included a “Random” method as a reference for comparison. The details of each method are as follows:

1. Random: Randomly select 2,000 genes from the datasets.
2. Seurat Disp: Uses the FindVariableFeatures() function with “method = disp” from the Seurat package.
3. Seurat Vst: Uses the FindVariableFeatures() function with “method = vst” from the Seurat package, which is equivalent to the scran method described in Section 2.
4. Seurat Mvp: Uses the FindVariableFeatures() function with “method = mvp” from the Seurat package.
5. Brennecke: Uses the BrenneckeGetVariableGenes() function from the M3Drop package.
6. NBdrop: Uses the NBumiFeatureSelectionCombinedDrop() function from the M3Drop R package.

M3drop: Uses the M3DropFeatureSelection() function from the M3Drop R package.

## Supporting information

S1 Table Number of HVGs selected by different feature selection methods by default. (Simulated datasets).
(DOCX)

S2 Table Highly variable gene designation for simulated dataset. (DOCX)

S3 Table Summary statistics of PBMC datasets. (DOCX)

S1 Fig Impact of varying splitting probabilities (0.1 to 0.4) on gene selection. (DOCX)

S2 Fig Density plot of log mean expression for selected genes in PBMC coarse-resolution datasets. (DOCX)

S3 Fig Comparison of the mean gene expression of gene CHMP7 by different fine resolution PBMC cell types. (DOCX)

S4 Fig Comparison of the mean gene expression of gene ADPRM by different fine resolution PBMC cell types. (DOCX)

S5 Fig Comparison of the mean gene expression of gene CITED4 by different fine resolution PBMC cell types. (DOCX)

S6 Fig Comparison of the mean gene expression of gene BCAS4 by different fine resolution PBMC cell types. (DOCX)

S7 Fig Silhouette score for comparing feature selection performance on PBMC and simulated datasets. (DOCX)

S8 Fig Purity for comparing feature selection performance on PBMC and simulated datasets. (DOCX)

S9 Fig ARI for comparing feature selection performance on PBMC and simulated datasets. (DOCX)

S10 Fig NMI for comparing feature selection performance on PBMC and simulated datasets. (DOCX)

S11 Fig Neighborhood hit for comparing feature selection performance on PBMC and simulated datasets. (DOCX)

S12 Fig The trend of mean averaged clustering metrics as the number of selected genes increases on PBMC and simulated datasets. (DOCX)

S13 Fig Randomly generated phylogenetic tree (bifurcating tree). (DOCX)

S14 Fig Randomly generated phylogenetic tree (three nodes). (DOCX)

S15 Fig Randomly generated phylogenetic tree (four nodes). (DOCX)

S16 Fig Randomly generated phylogenetic tree (five nodes). (DOCX)

S17 Fig Randomly generated phylogenetic tree (six nodes). (DOCX)

S18 Fig UMAP visualization of the same fine-resolution PBMC dataset with Fig 14 but in different random seed, with true annotated labels and *k*-means clustering labels by different FS methods. (DOCX)

S19 Fig UMAP visualization of the same fine-resolution PBMC dataset with Fig 14 but in different random seed, with true annotated labels and *k*-means clustering labels by different FS methods. (DOCX)

S20 Fig UMAP visualization of the same fine-resolution PBMC dataset with Fig 14, colored by true annotated labels by different FS methods. (DOCX)

S21 Fig Comparison of Mean Jaccard Similarity with and without pre-processing. (DOCX)

S22 Fig Running time of different FS methods as number of cells and number of genes increase. (DOCX)

S1 Methods Proof of Eq 22. (DOCX)
