# Supplementary material for: Mcadet: A feature selection method for fine-resolution single-cell RNA-seq data based on multiple correspondence analysis and community detection
Source: PLoS Comput Biol. 2024 Oct 28;20(10):e1012560. doi: 10.1371/journal.pcbi.1012560 (PMC11542852; doi:10.1371/journal.pcbi.1012560)
Supplement: S1 Table — (Simulated datasets). (DOCX) [file pcbi.1012560.s001.docx]

**Table S1. Number of HVGs selected by different feature selection methods by default. (Simulated datasets)**

| Simulated | Coarse-resolution datasets  2000 (2000, 2000) | | Fine-resolution datasets  2000 (2000, 2000) | |
| --- | --- | --- | --- | --- |
|  | N  Median (IQR) | Jaccard Similarity  Median (IQR) | N  Median (IQR) | Jaccard Similarity  Median (IQR) |
| Mcadet | 1356 (1288, 1424) | 0.42 (0.41, 0.43) | 924 (758, 1087) | 0.28 (0.23, 0.34) |
| NBDrop | 506 (473, 547) | 0.17 (0.15, 0.19) | 314 (273, 345) | 0.07 (0.06, 0.09) |
| M3Drop | 6303 (6253, 6365) | 0.13 (0.12, 0.13) | 6420 (6392, 6451) | 0.11 (0.11, 0.12) |
| Brennecke | 1385 (1336, 1434) | 0.20 (0.16, 0.23) | 1231 (1188, 1256) | 0.09 (0.08, 0.10) |
| Seurat Mvp | 682 (640, 713) | 0.23 (0.21, 0.26) | 547 (515, 577) | 0.12 (0.10, 0.14) |
| Seurat Vst | 2000 (2000, 2000) | 0.27 (0.25, 0.29) | 2000 (2000, 2000) | 0.19 (0.16, 0.20) |
| Seurat Disp | 2000 (2000, 2000) | 0.20 (0.19, 0.22) | 2000 (2000, 2000) | 0.14 (0.13, 0.15) |
| Scry | 2000 (2000, 2000) | 0.10 (0.10, 0.11) | 2000 (2000, 2000) | 0.09 (0.09, 0.09) |
| random | 2000 (2000, 2000) | 0.07 (0.07, 0.07) | 2000 (2000, 2000) | 0.07 (0.07, 0.07) |
