# Supplementary material for: Mcadet: A feature selection method for fine-resolution single-cell RNA-seq data based on multiple correspondence analysis and community detection
Source: PLoS Comput Biol. 2024 Oct 28;20(10):e1012560. doi: 10.1371/journal.pcbi.1012560 (PMC11542852; doi:10.1371/journal.pcbi.1012560)
Supplement: S2 Table — (DOCX) [file pcbi.1012560.s002.docx]

| Cell population  (N, %) | Driver genes designation | HVGs designation  (Drive gene excluded) | Number of HVGs (N, %)  (Driver gene included) |
| --- | --- | --- | --- |
| Type 1 (60, 1) | Gene 1-20 | Gene 201-400, 801-1000, 1401-1600 | 620 (4.1) |
| Type 2 (180, 3) | Gene 21-40 | Gene 401-600, 801-1000, 1201-1400, 1601-1800 | 820 (5.5) |
| Type 3 (240, 4) | Gene 41-60 | Gene 201-600, 1401-1600, 1801-2000 | 820 (5.5) |
| Type 4 (360, 6) | Gene 61-80 | Gene 401-600, 1001-1600, 1801-2000 | 1000 (6.7) |
| Type 5 (420, 7) | Gene 81-100 | Gene 201-400, 601-800, 1601-2000 | 820 (5.5) |
| Type 6 (480, 8) | Gene 101-120 | Gene 401-600, 1001-1200, 1401-1600, 1801-2000 | 820 (5.5) |
| Type 7 (600, 10) | Gene 121-140 | Gene 201-400, 601-800, 1201-1400 | 620 (4.1) |
| Type 8 (960 16) | Gene 141-160 | Gene 601-800, 1001-1200, 1601-1800 | 620 (4.1) |
| Type 9 (1200, 20) | Gene 161-180 | Gene 201-400, 1201-1400, 1601-2000 | 820 (5.5) |
| Type 10 (1500, 25) | Gene 181-200 | Gene 801-1200, 1401-1800 | 820 (5.5) |

**Table S2. Highly variable gene designation for simulated dataset**
