# Supplementary material for: Mcadet: A feature selection method for fine-resolution single-cell RNA-seq data based on multiple correspondence analysis and community detection
Source: PLoS Comput Biol. 2024 Oct 28;20(10):e1012560. doi: 10.1371/journal.pcbi.1012560 (PMC11542852; doi:10.1371/journal.pcbi.1012560)
Supplement: S3 Table — (DOCX) [file pcbi.1012560.s003.docx]

**Table S3. Summary statistics of PBMC datasets**

| Datasets | | Coarse-resolution datasets | | Fine-resolution datasets | |
| --- | --- | --- | --- | --- | --- |
| Donor | Batch | Number of Cells | Number of HVGs | Number of Cells | Number of HVGs |
| 1 | 1 | 6,443 | 1,650 | 3,440 | 1,615 |
| 1 | 2 | 5,917 | 1,605 | 3,481 | 1,671 |
| 1 | 3 | 5,775 | 1,642 | 3,011 | 1,615 |
| 2 | 1 | 5,978 | 1,646 | 3,434 | 1,601 |
| 2 | 2 | 5,714 | 1,586 | 3,644 | 1,561 |
| 2 | 3 | 5,513 | 1,635 | 3,175 | 1,542 |
| 3 | 1 | 4,698 | 1,695 | 2,105 | 1,113 |
| 3 | 2 | 5,002 | 1,656 | 1,997 | 1,200 |
| 3 | 3 | 4,960 | 1,656 | 2,677 | 1,247 |
| 4 | 1 | 5,307 | 1,653 | 2,851 | 1,305 |
| 4 | 2 | 5,793 | 1,629 | 3,755 | 1,463 |
| 4 | 3 | 5,990 | 1,584 | 2,809 | 1,159 |
| 5 | 1 | 7,020 | 1,669 | 2,394 | 2,352 |
| 5 | 2 | 6,933 | 1,668 | 2,089 | 2,200 |
| 5 | 3 | 7,858 | 1,746 | 3,105 | 2,486 |
| 6 | 1 | 6,093 | 1,684 | 1,510 | 1,183 |
| 6 | 2 | 7,583 | 1,839 | 3,318 | 1,841 |
| 6 | 3 | 7,066 | 1,731 | 1,776 | 1,555 |
| 7 | 1 | 8,909 | 1,597 | 2,645 | 2,448 |
| 7 | 2 | 8,200 | 1,756 | 3,769 | 2,302 |
| 7 | 3 | 8,762 | 1,712 | 2,963 | 2,076 |
| 8 | 1 | 8,916 | 1,723 | 4,026 | 2,602 |
| 8 | 2 | 9,203 | 1,736 | 4,990 | 2,809 |
| 8 | 3 | 8,131 | 1,752 | 4,076 | 2,473 |
