# Supplementary material for: Mcadet: A feature selection method for fine-resolution single-cell RNA-seq data based on multiple correspondence analysis and community detection
Source: PLoS Comput Biol. 2024 Oct 28;20(10):e1012560. doi: 10.1371/journal.pcbi.1012560 (PMC11542852; doi:10.1371/journal.pcbi.1012560)
Supplement: S1 Fig — (DOCX) [file pcbi.1012560.s004.docx]

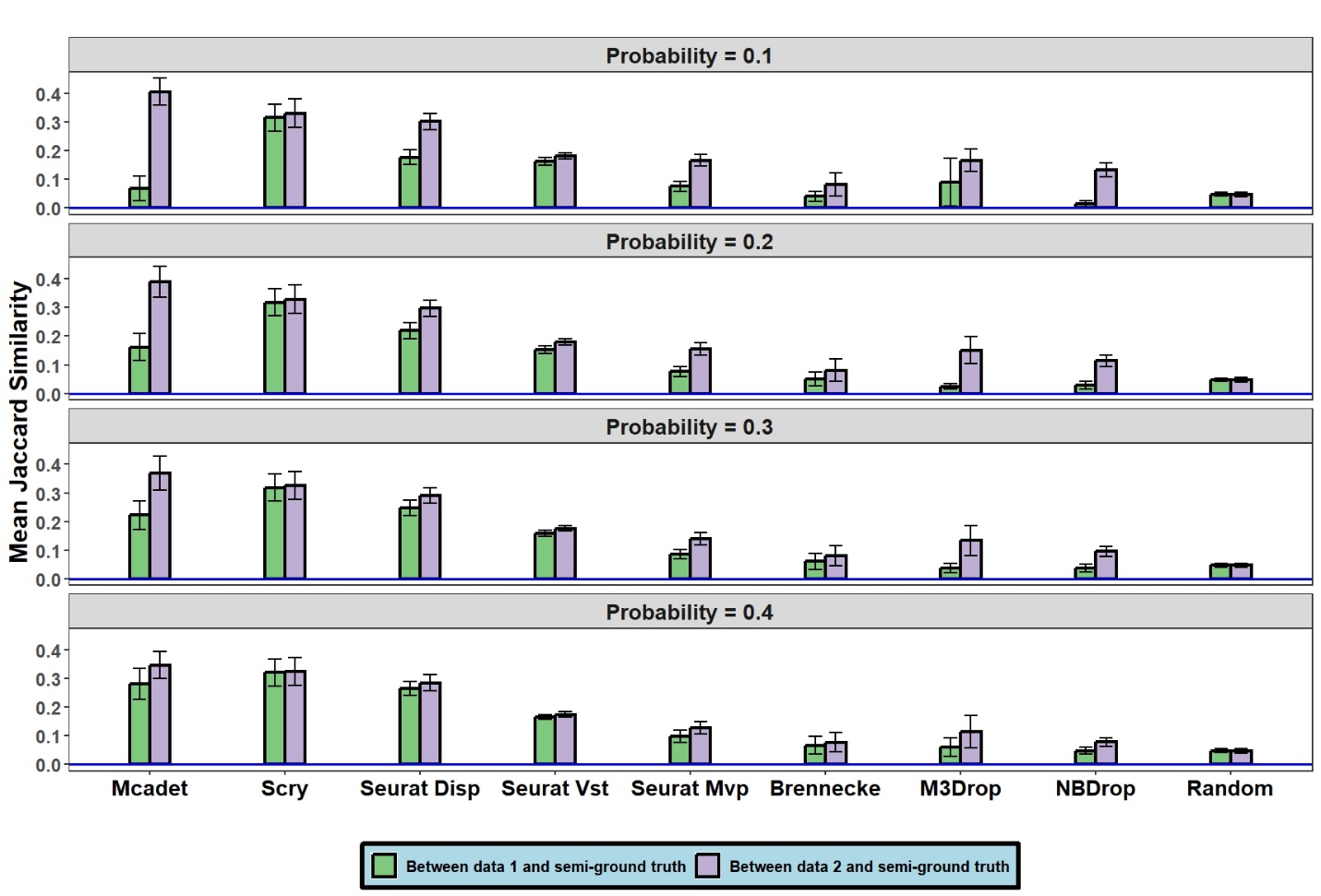


**Figure S1: Impact of varying splitting probabilities (0.1 to 0.4) on gene selection.** The top panel shows 0.1 (left) vs. 0.9 (right). Error bars represent the standard deviations. Data 1 and 2 are the two split datasets of original datasets.
