# Supplementary material for: Mcadet: A feature selection method for fine-resolution single-cell RNA-seq data based on multiple correspondence analysis and community detection
Source: PLoS Comput Biol. 2024 Oct 28;20(10):e1012560. doi: 10.1371/journal.pcbi.1012560 (PMC11542852; doi:10.1371/journal.pcbi.1012560)
Supplement: S2 Fig — (DOCX) [file pcbi.1012560.s005.docx]

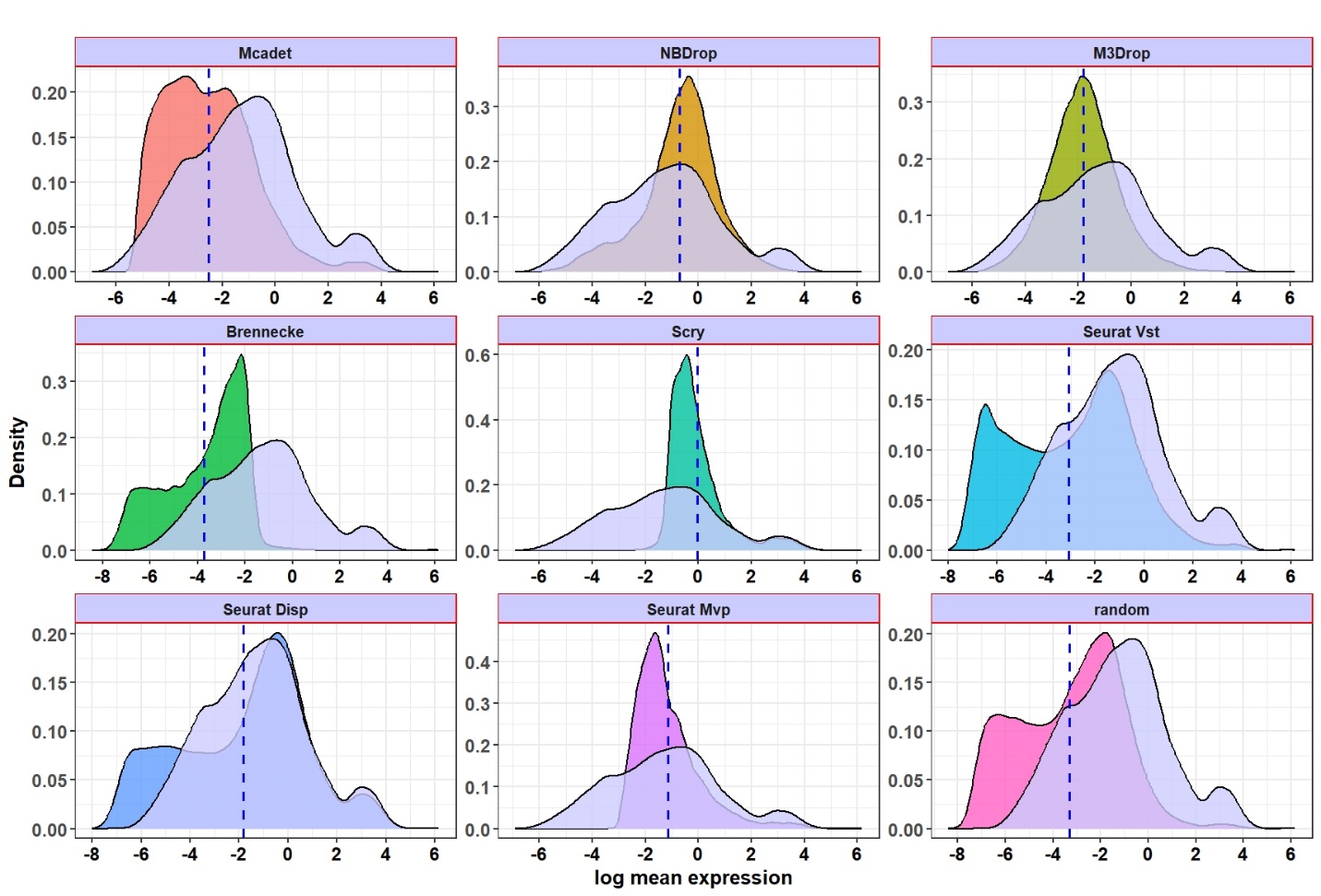


**Figure S2: Density plot of log mean expression for selected genes in PBMC coarse-resolution datasets.**
