# Supplementary material for: Mcadet: A feature selection method for fine-resolution single-cell RNA-seq data based on multiple correspondence analysis and community detection
Source: PLoS Comput Biol. 2024 Oct 28;20(10):e1012560. doi: 10.1371/journal.pcbi.1012560 (PMC11542852; doi:10.1371/journal.pcbi.1012560)
Supplement: S3 Fig — (DOCX) [file pcbi.1012560.s006.docx]

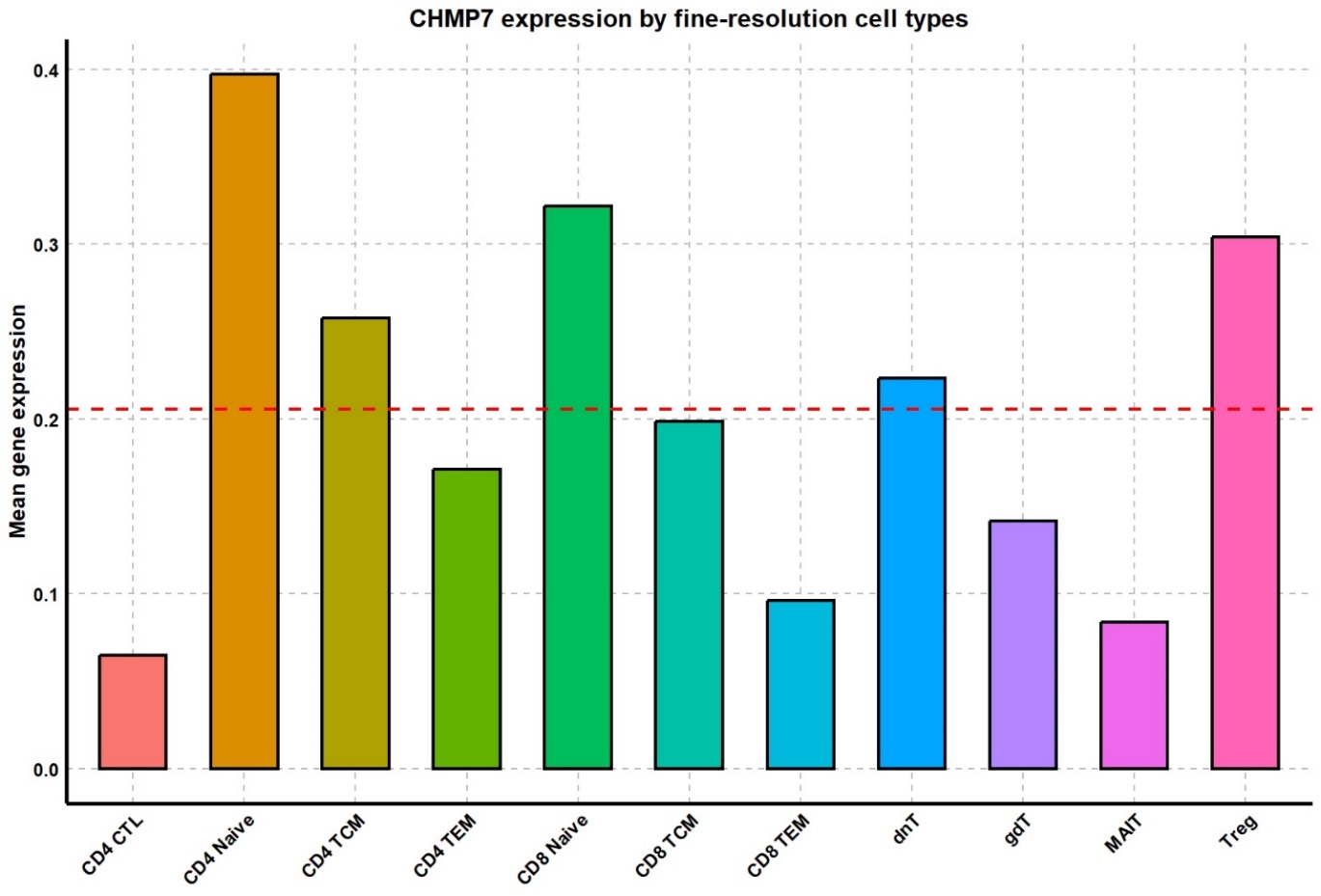


**Figure S3: Comparison of the mean gene expression of gene CHMP7 by different fine-resolution PBMC cell types.**

CHMP7 (Charged Multivesicular Body Protein 7): It is involved in endosomal sorting and the formation of multivesicular bodies. It plays a role in the ESCRT (endosomal sorting complexes required for transport) pathway, which is crucial for various cellular processes including membrane repair, cytokinesis, and viral budding [1].

1. Dombecki CR, Chiang AC, Kang HJ, Bilgir C, Stefanski NA, Neva BJ, et al. The chromodomain protein MRG-1 facilitates SC-independent homologous pairing during meiosis in Caenorhabditis elegans. Developmental cell. 2011 Dec 13;21(6):1092-103.
