# Supplementary material for: Mcadet: A feature selection method for fine-resolution single-cell RNA-seq data based on multiple correspondence analysis and community detection
Source: PLoS Comput Biol. 2024 Oct 28;20(10):e1012560. doi: 10.1371/journal.pcbi.1012560 (PMC11542852; doi:10.1371/journal.pcbi.1012560)
Supplement: S4 Fig — (DOCX) [file pcbi.1012560.s007.docx]

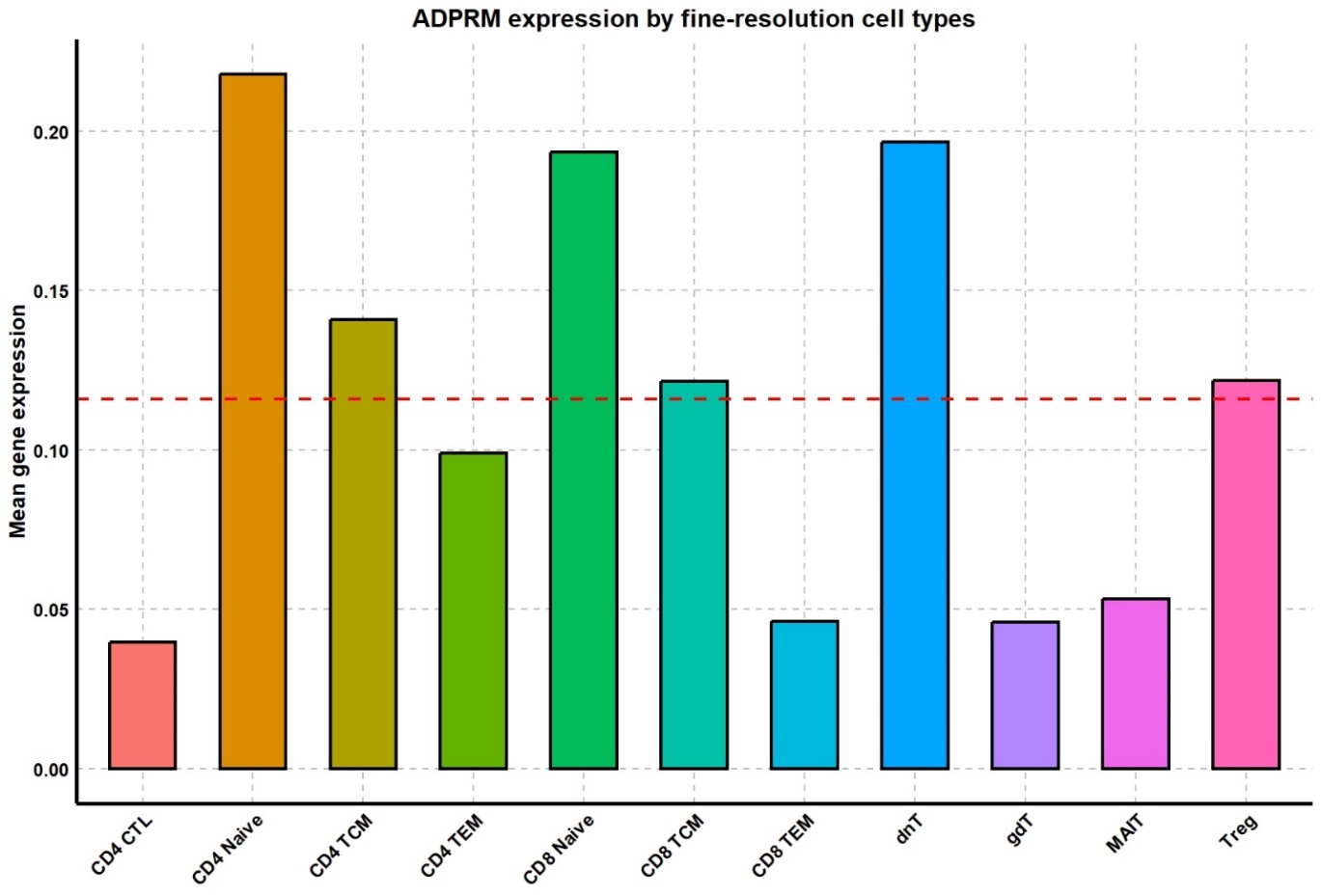


**Figure S4: Comparison of the mean gene expression of gene ADPRM by different fine-resolution PBMC cell types.**

ADPRM (ADP-ribose/CDP-alcohol diphosphatase, manganese-dependent): It hydrolyzes ADP-ribose and CDP-alcohols. It is involved in immune cell signaling and may play a role in cellular response to oxidative stress [1].

1. Uhlén M, Fagerberg L, Hallström BM, et al. Proteomics. 2015; Available from: [Human Protein Atlas](https://www.proteinatlas.org/ENSG00000170222-ADPRM).
