# Supplementary material for: Mcadet: A feature selection method for fine-resolution single-cell RNA-seq data based on multiple correspondence analysis and community detection
Source: PLoS Comput Biol. 2024 Oct 28;20(10):e1012560. doi: 10.1371/journal.pcbi.1012560 (PMC11542852; doi:10.1371/journal.pcbi.1012560)
Supplement: S5 Fig — (DOCX) [file pcbi.1012560.s008.docx]

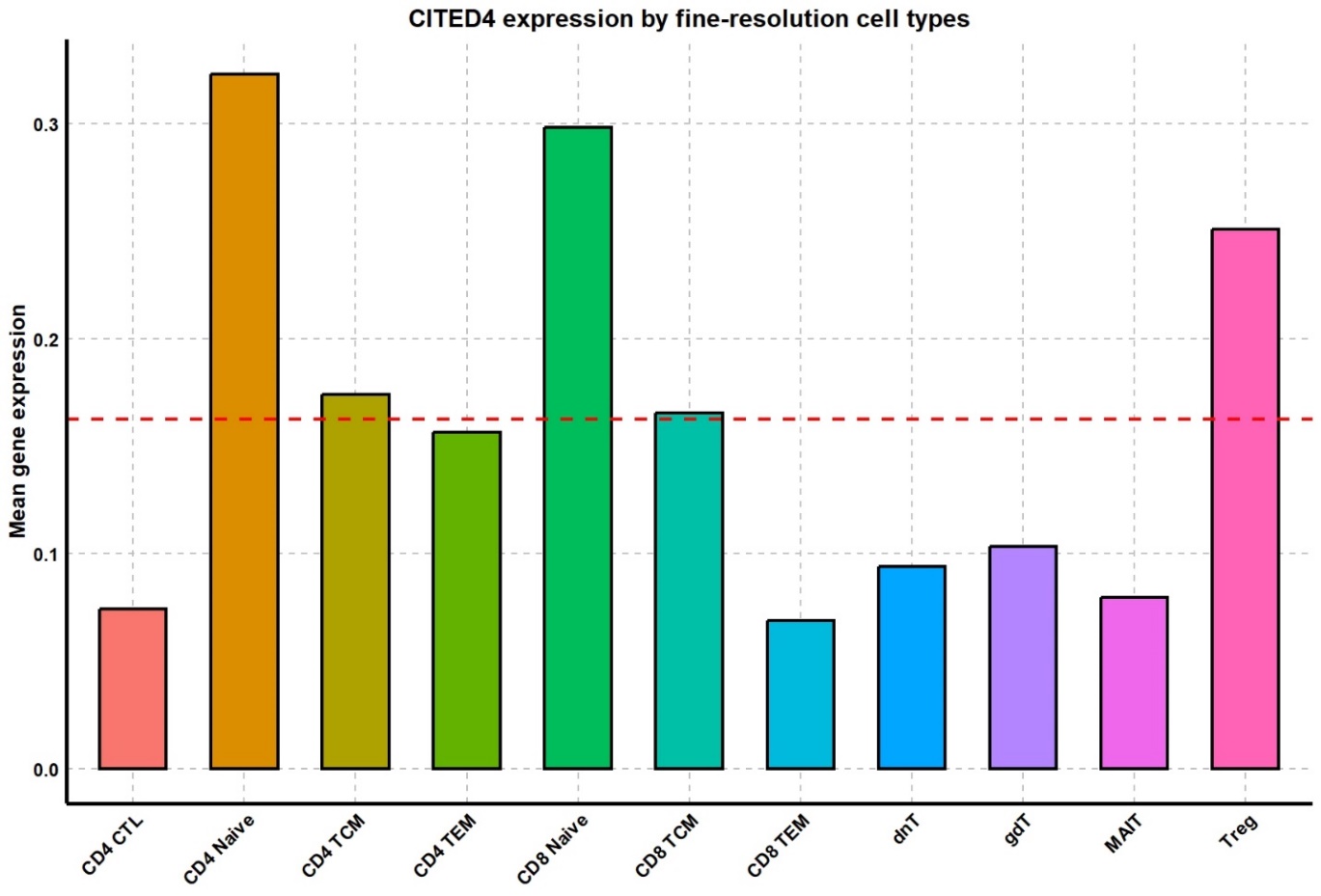


**Figure S5: Comparison of the mean gene expression of gene CITED4 by different fine-resolution PBMC cell types.**

CITED4 (Cbp/p300-Interacting Transactivator with Glu/Asp-Rich Carboxy-Terminal Domain 4): It is a transcriptional coactivator involved in the regulation of gene expression, particularly in response to hypoxia. It interacts with the transcription factor HIF-1α and p300/CBP to enhance the transcription of hypoxia-responsive genes [1].

1. Bhattacharya S, Michels CL, Leung MK, Arany ZP, Kung AL, Livingston DM. Functional role of p35srj, a novel p300/CBP binding protein, during transactivation by HIF-1. Genes & development. 1999 Jan 1;13(1):64-75.
