# Supplementary material for: Mcadet: A feature selection method for fine-resolution single-cell RNA-seq data based on multiple correspondence analysis and community detection
Source: PLoS Comput Biol. 2024 Oct 28;20(10):e1012560. doi: 10.1371/journal.pcbi.1012560 (PMC11542852; doi:10.1371/journal.pcbi.1012560)
Supplement: S6 Fig — (DOCX) [file pcbi.1012560.s009.docx]

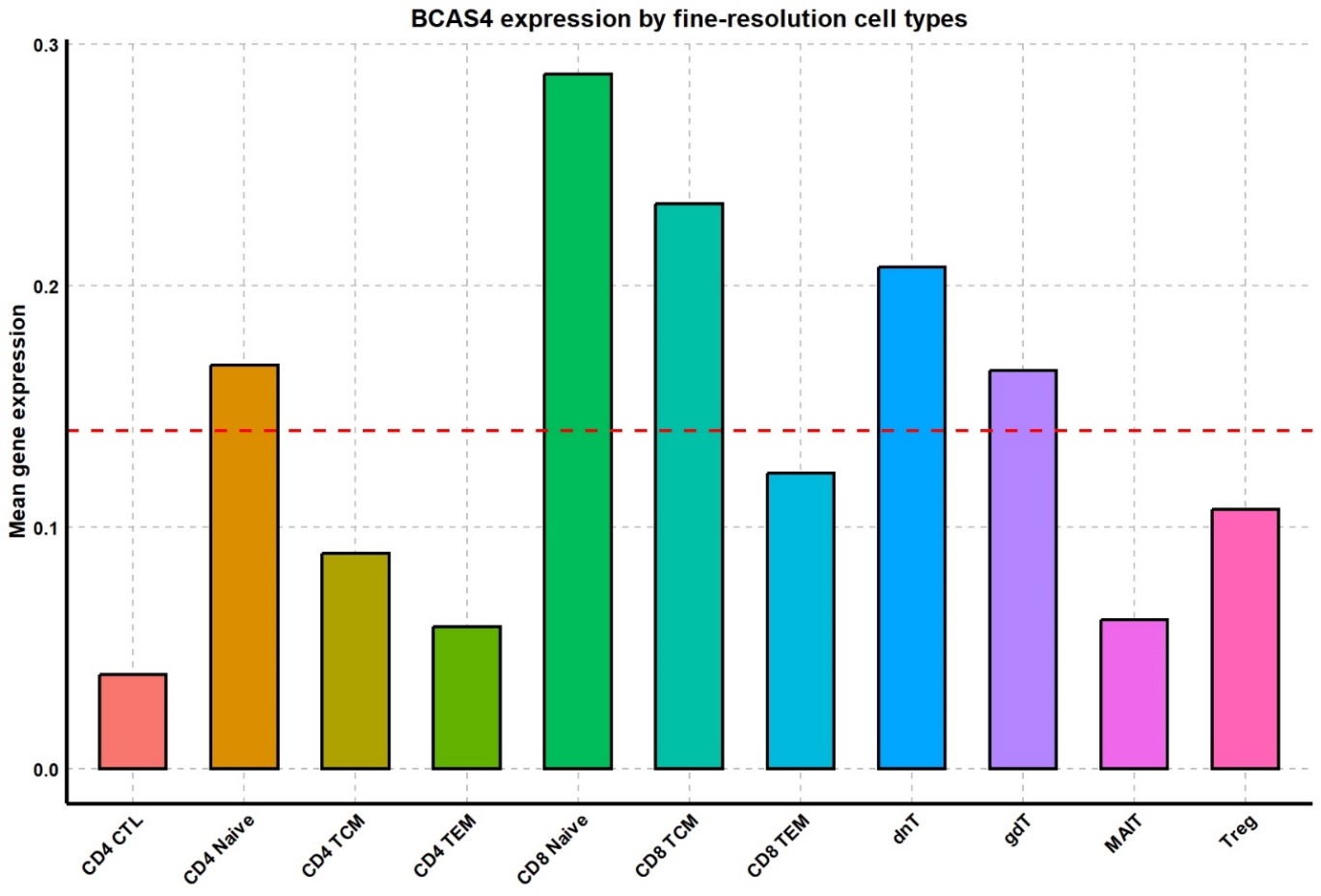


**Figure S6: Comparison of the mean gene expression of gene BCAS4 by different fine-resolution PBMC cell types.**

BCAS4 (Breast Carcinoma Amplified Sequence 4): It is implicated in the progression of breast cancer. It is often found co-amplified with BCAS3 in breast cancer and may play a role in tumorigenesis by affecting cell proliferation and survival [1].

1. Nguyen JT, Hoopes JD, Le MH, Smee DF, Patick AK, Faix DJ, et al. Triple combination of amantadine, ribavirin, and oseltamivir is highly active and synergistic against drug resistant influenza virus strains in vitro. PloS one. 2010 Feb 22;5(2):e9332.
