# Supplementary figures and images for: Mcadet: A feature selection method for fine-resolution single-cell RNA-seq data based on multiple correspondence analysis and community detection
Source: PLoS Comput Biol. 2024 Oct 28;20(10):e1012560. doi: 10.1371/journal.pcbi.1012560 (PMC11542852; doi:10.1371/journal.pcbi.1012560)

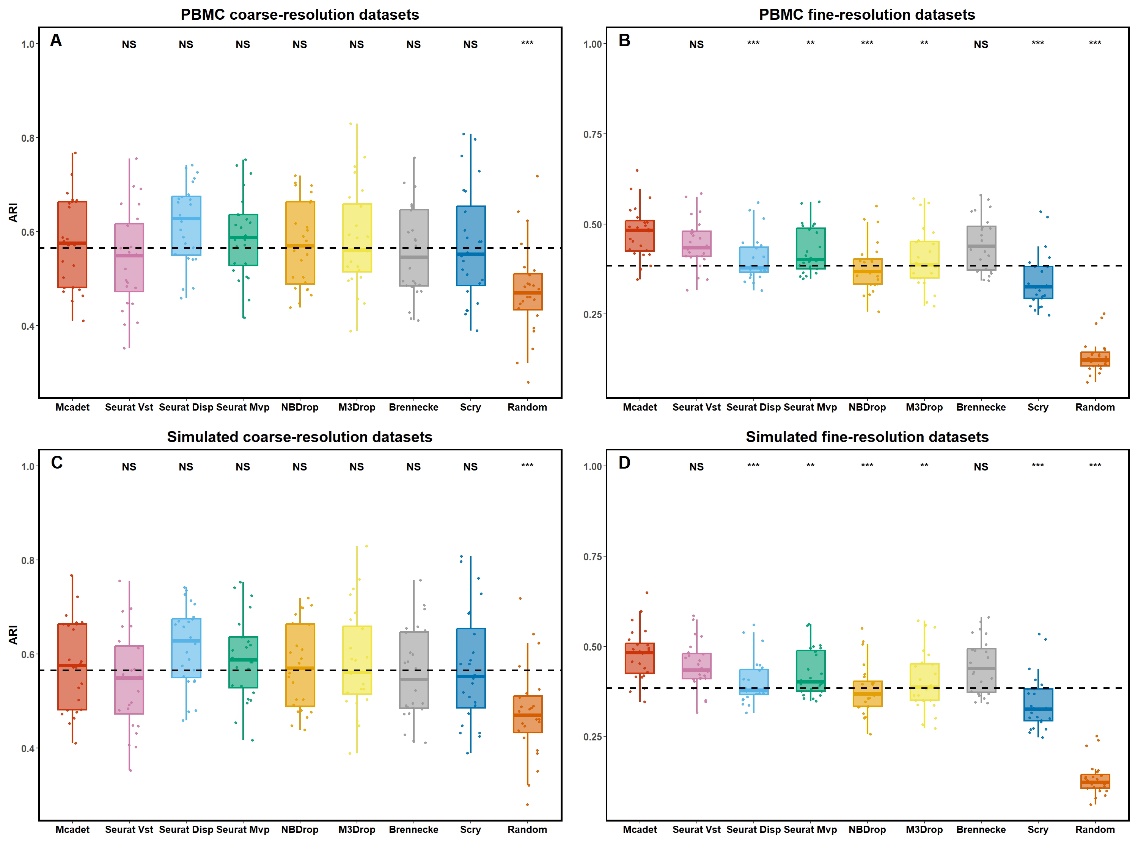


**Figure S9. ARI for comparing feature selection performance on PBMC (A & B) and simulated datasets (C & D).**

Supplement: S9 Fig — (DOCX) [file pcbi.1012560.s012.docx]

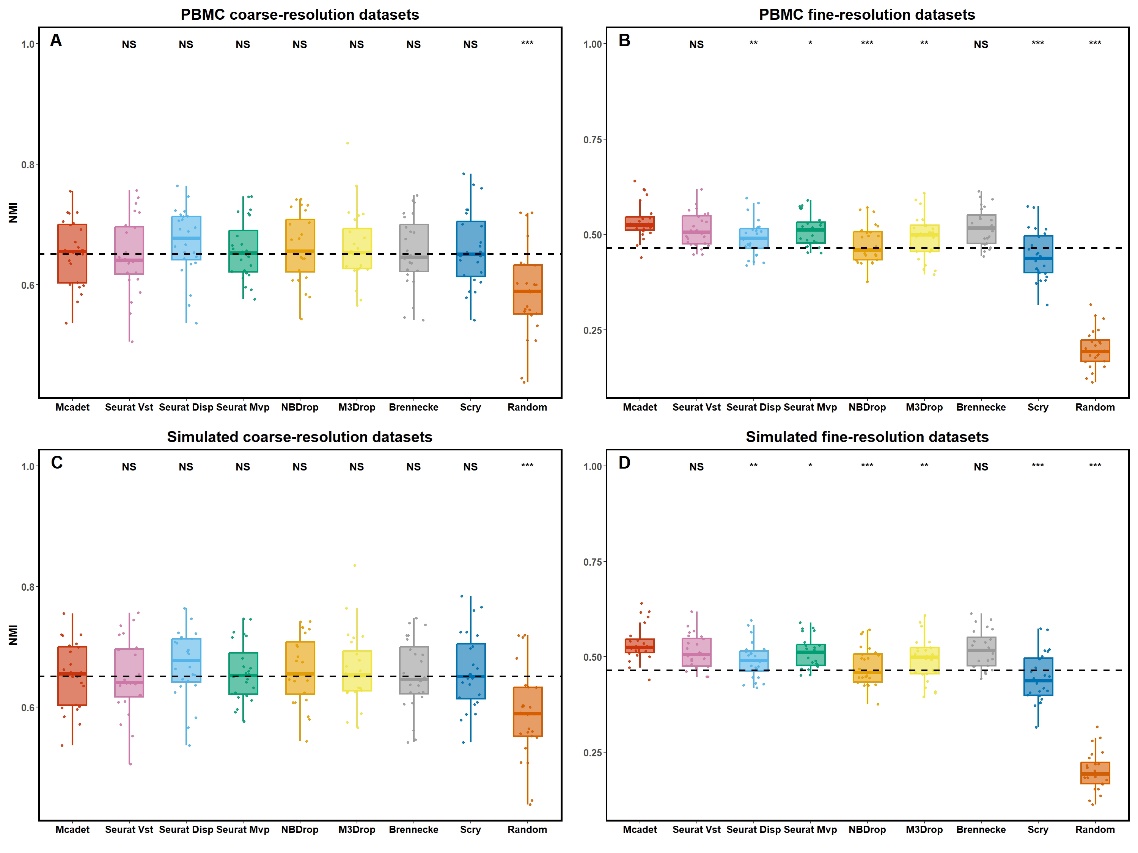


**Figure S10. NMI for comparing feature selection performance on PBMC (A & B) and simulated datasets (C & D).**

Supplement: S10 Fig — (DOCX) [file pcbi.1012560.s013.docx]

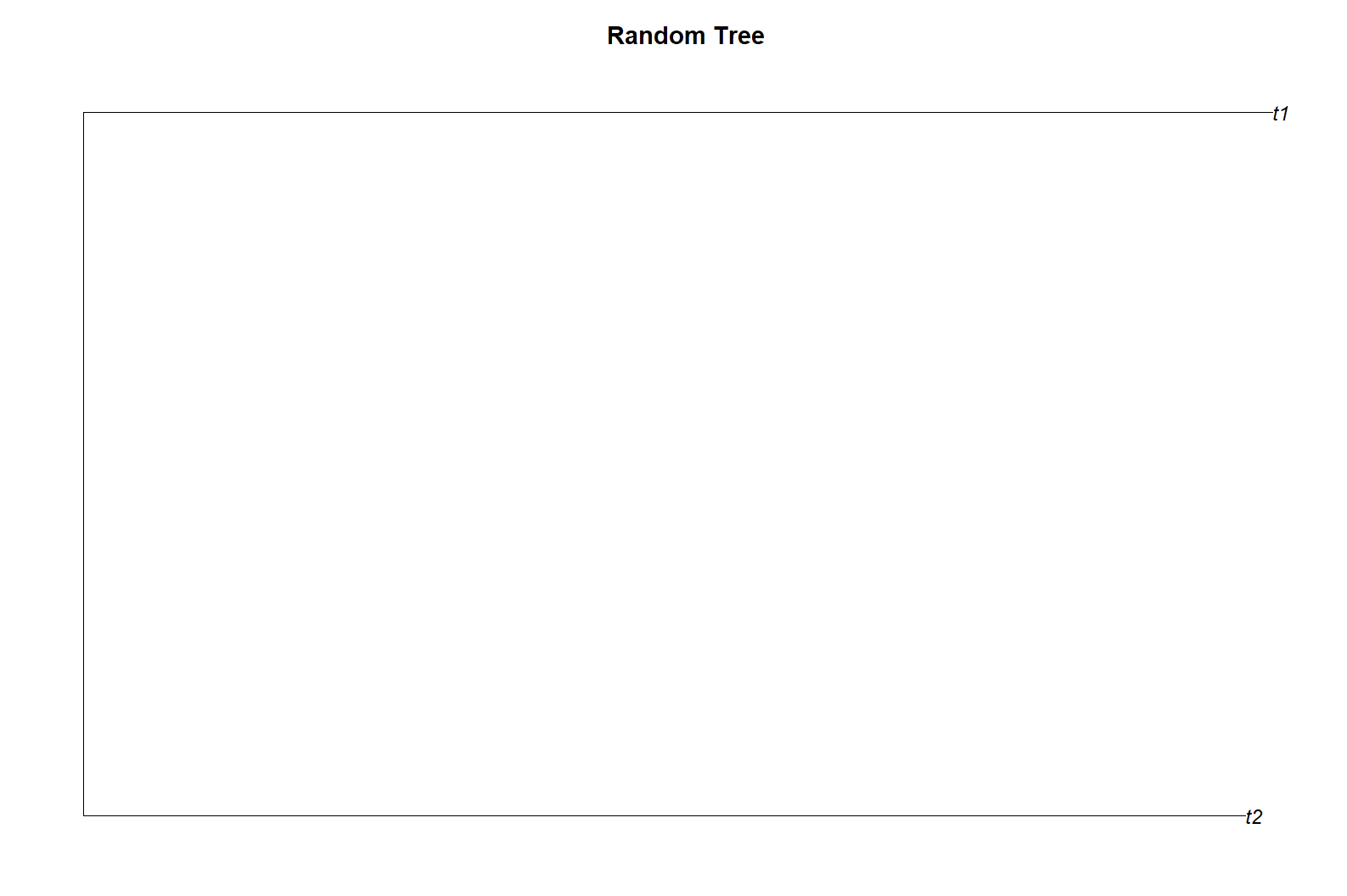


**Figure S13. Randomly generated phylogenetic tree (bifurcating tree)**

Supplement: S13 Fig — (DOCX) [file pcbi.1012560.s016.docx]

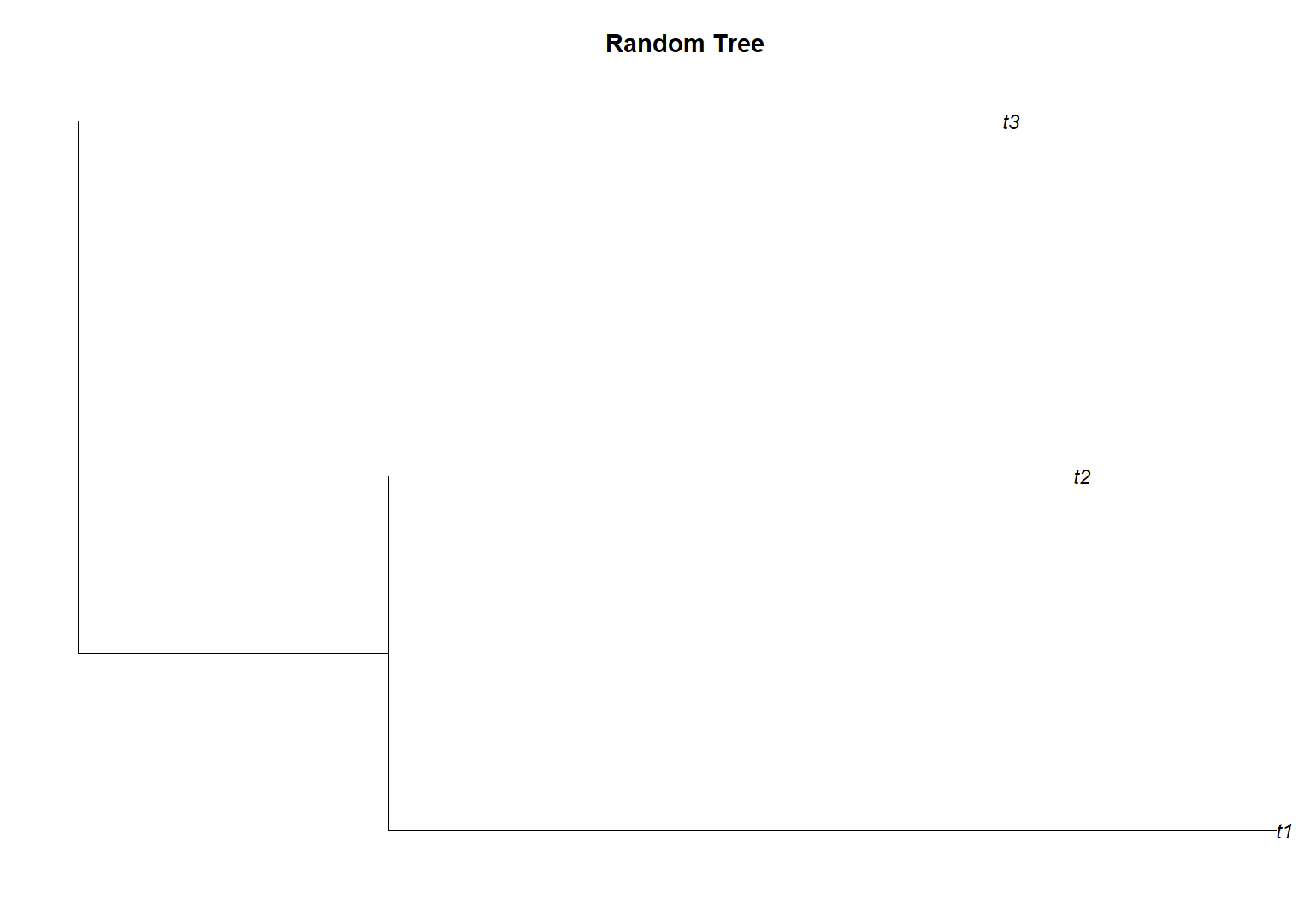


**Figure S14. Randomly generated phylogenetic tree (three nodes)**

Supplement: S14 Fig — (DOCX) [file pcbi.1012560.s017.docx]

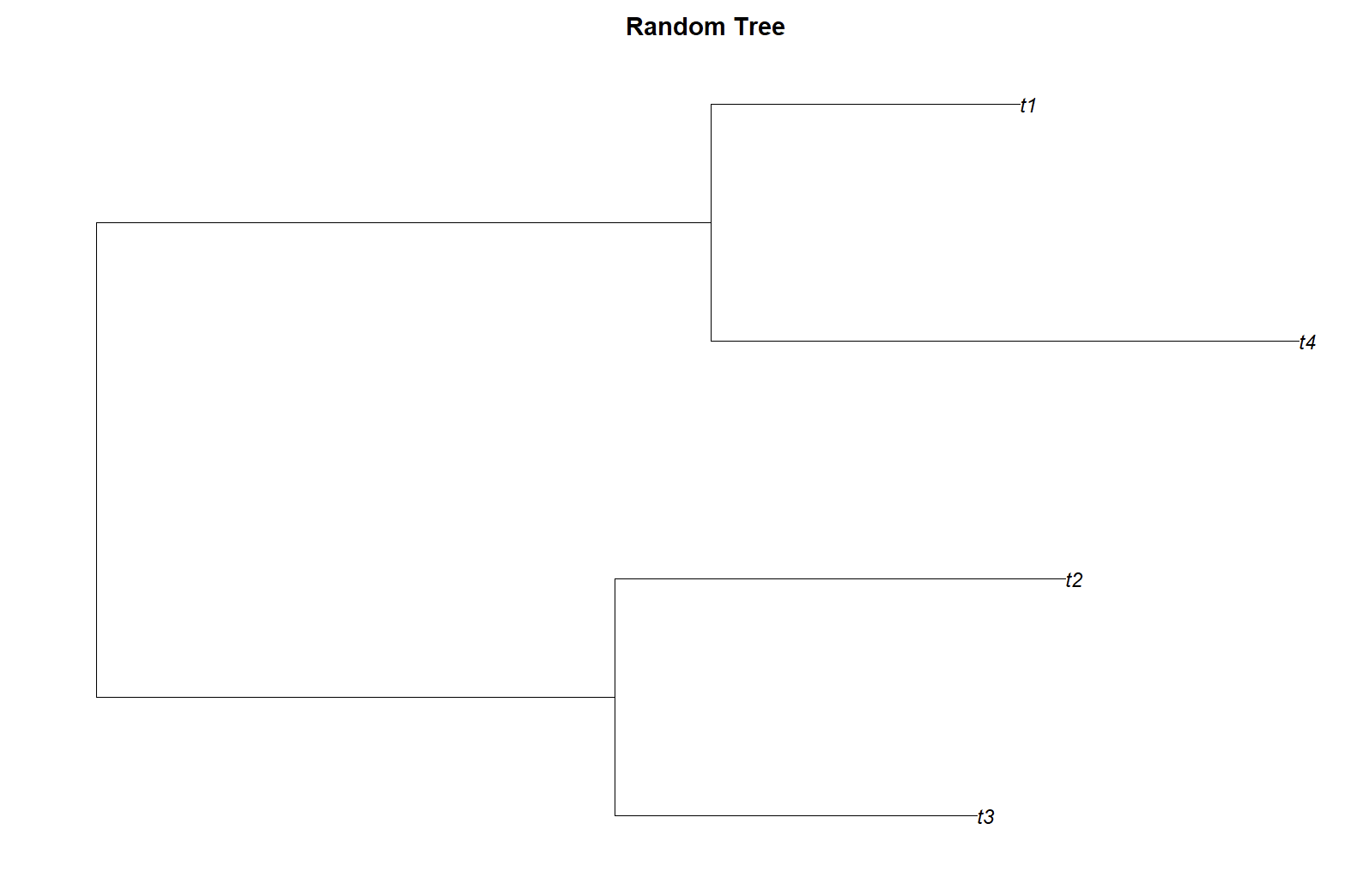


**Figure S15. Randomly generated phylogenetic tree (four nodes)**

Supplement: S15 Fig — (DOCX) [file pcbi.1012560.s018.docx]

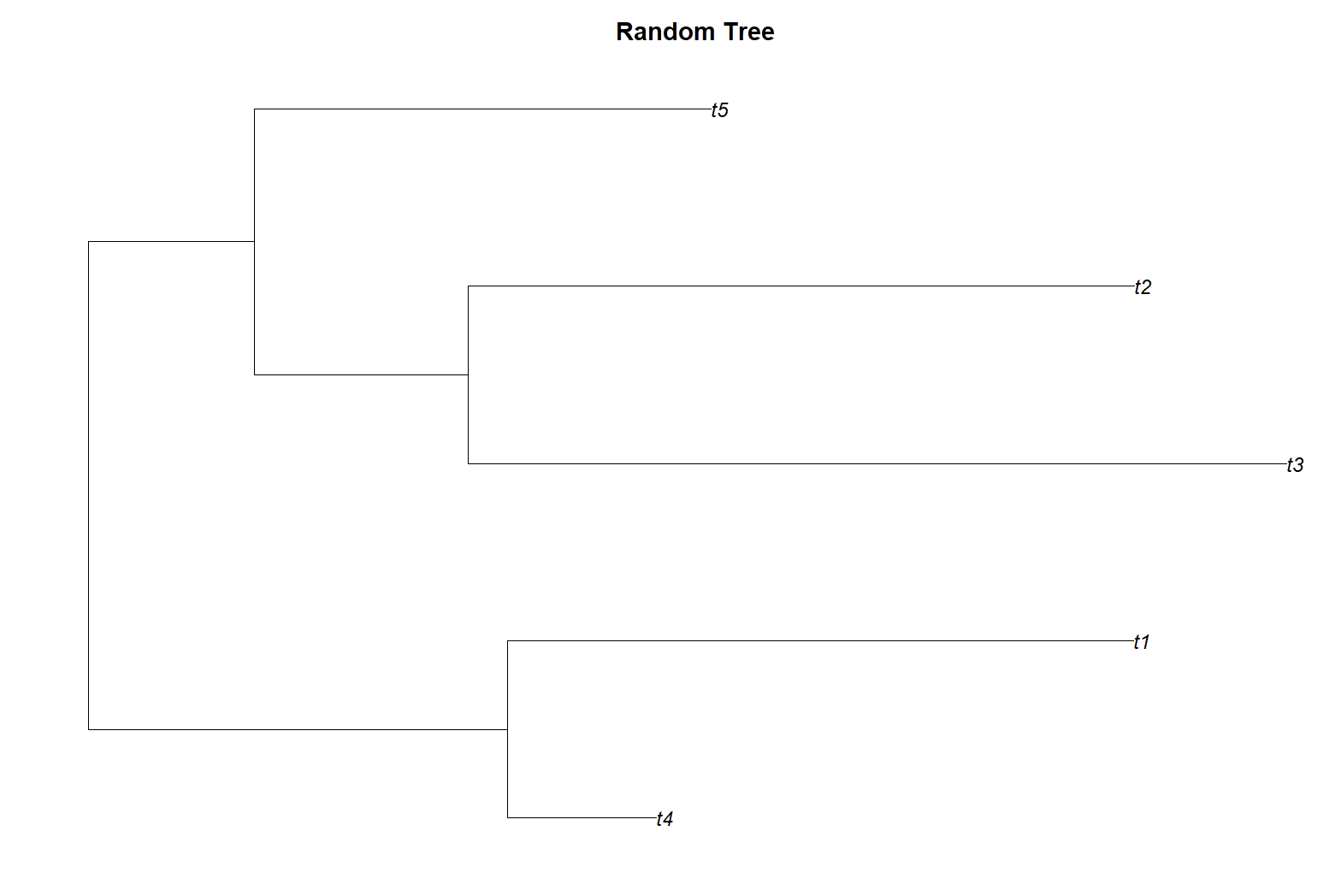


**Figure S16. Randomly generated phylogenetic tree (five nodes)**

Supplement: S16 Fig — (DOCX) [file pcbi.1012560.s019.docx]

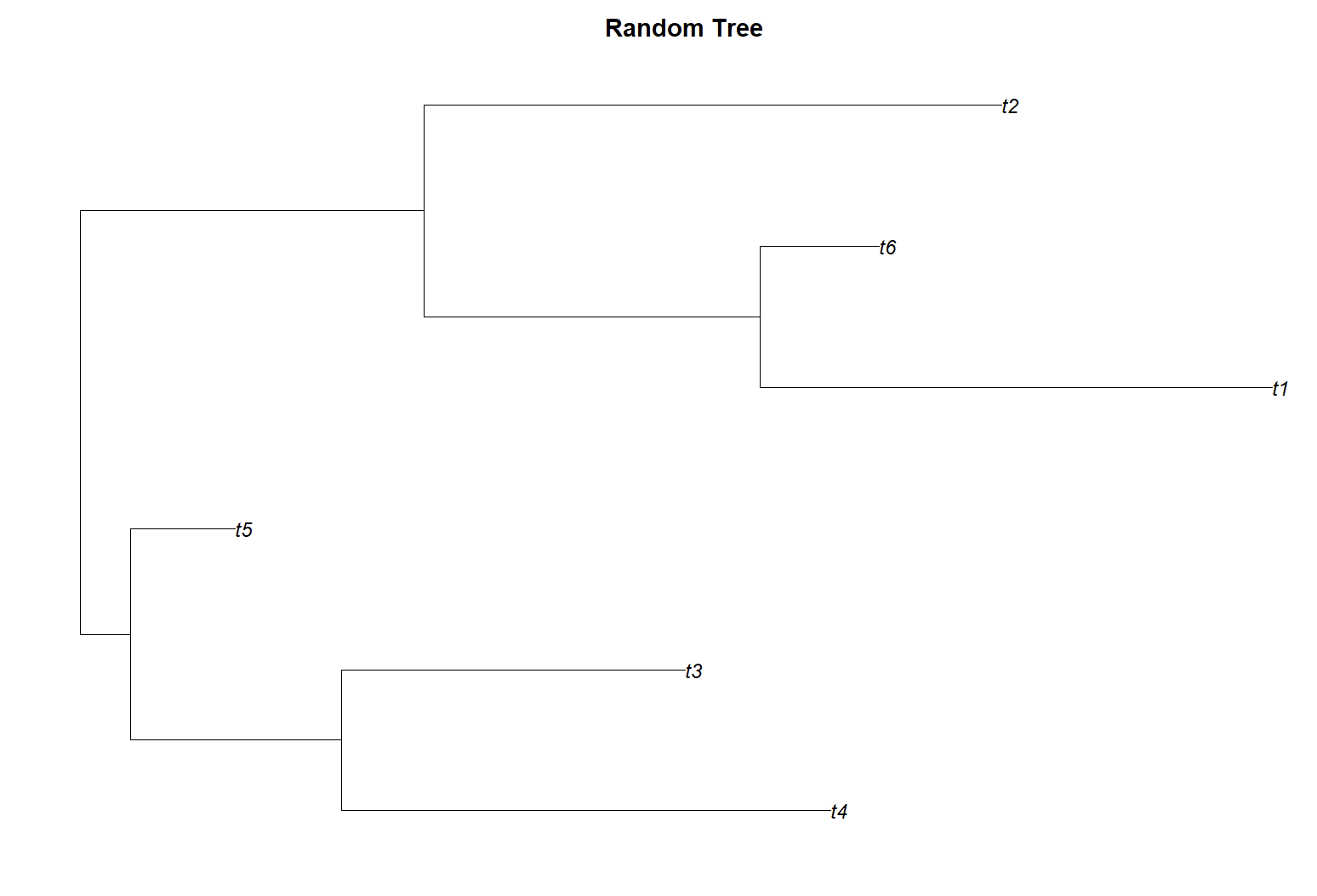


**Figure S17. Randomly generated phylogenetic tree (six nodes)**

Supplement: S17 Fig — (DOCX) [file pcbi.1012560.s020.docx]
