# Supplementary material for: Mcadet: A feature selection method for fine-resolution single-cell RNA-seq data based on multiple correspondence analysis and community detection
Source: PLoS Comput Biol. 2024 Oct 28;20(10):e1012560. doi: 10.1371/journal.pcbi.1012560 (PMC11542852; doi:10.1371/journal.pcbi.1012560)
Supplement: S12 Fig — (DOCX) [file pcbi.1012560.s015.docx]

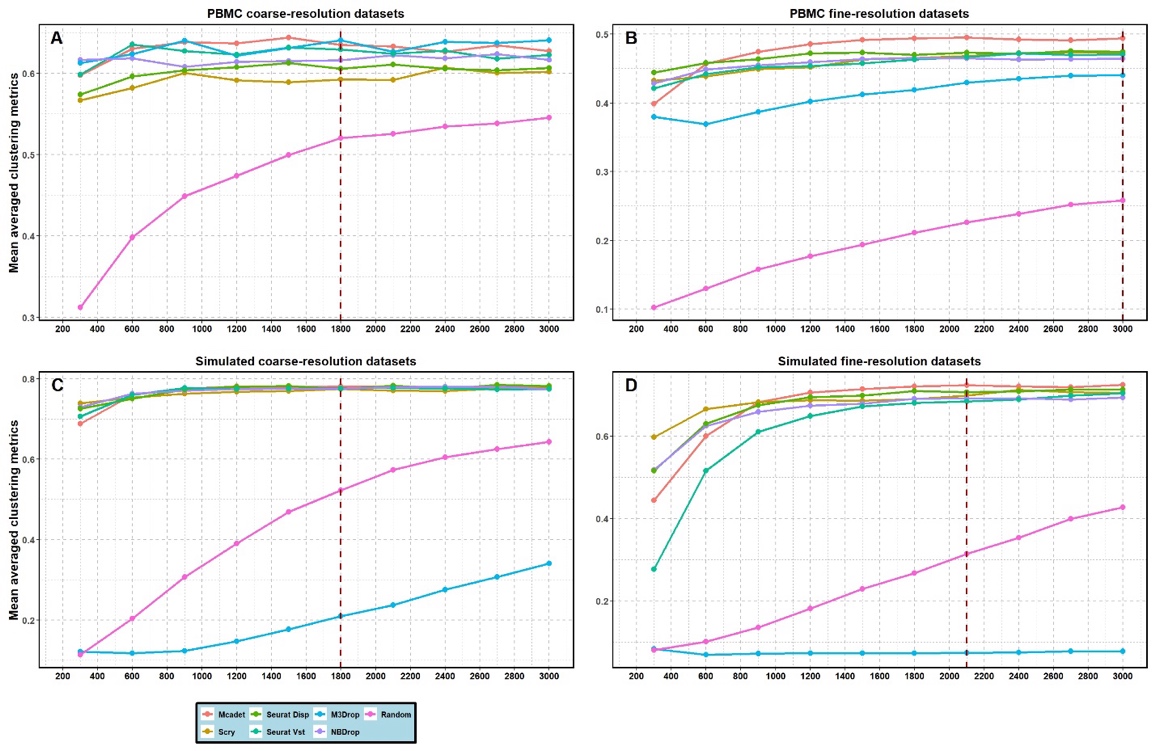


**Figure S12. The trend of mean averaged clustering metrics as the number of selected genes increases on PBMC (A and B) and simulated datasets (C and D).** The number of selected genes range from 200 to 3,000. The Brennecke method, which does not allow for specifying the number of HVGs needed, are excluded from this comparison.
