# Supplementary material for: Mcadet: A feature selection method for fine-resolution single-cell RNA-seq data based on multiple correspondence analysis and community detection
Source: PLoS Comput Biol. 2024 Oct 28;20(10):e1012560. doi: 10.1371/journal.pcbi.1012560 (PMC11542852; doi:10.1371/journal.pcbi.1012560)
Supplement: S20 Fig — (DOCX) [file pcbi.1012560.s023.docx]

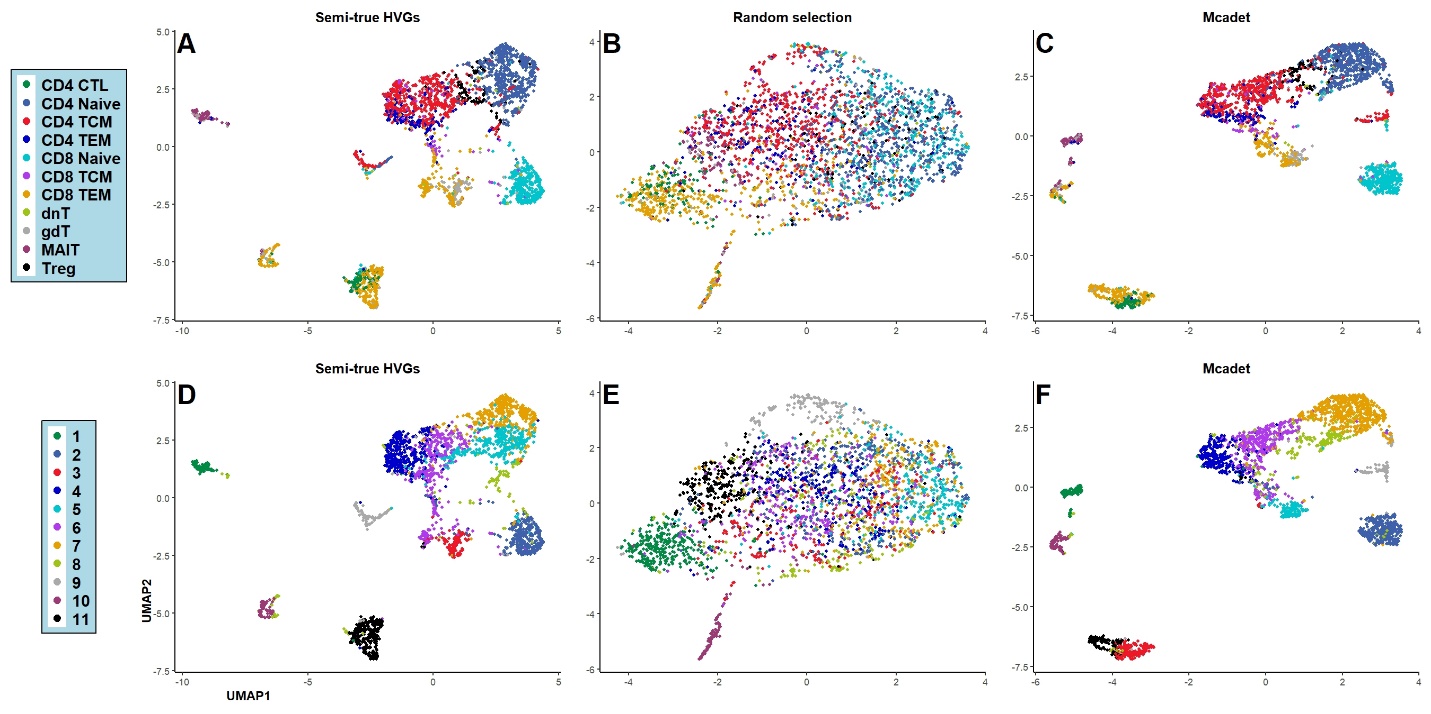


**Figure S20: UMAP visualization of the same fine-resolution PBMC dataset with Figure 14, with true annotated labels (A – C) and** $\boldsymbol{k}$**-means clustering labels (D – F) by HVGs of semi-ground truth, genes selected by random selection and HVGs selected by Mcadet.** (random seed = 123456)
