# Supplementary material for: Mcadet: A feature selection method for fine-resolution single-cell RNA-seq data based on multiple correspondence analysis and community detection
Source: PLoS Comput Biol. 2024 Oct 28;20(10):e1012560. doi: 10.1371/journal.pcbi.1012560 (PMC11542852; doi:10.1371/journal.pcbi.1012560)
Supplement: S21 Fig — (DOCX) [file pcbi.1012560.s024.docx]

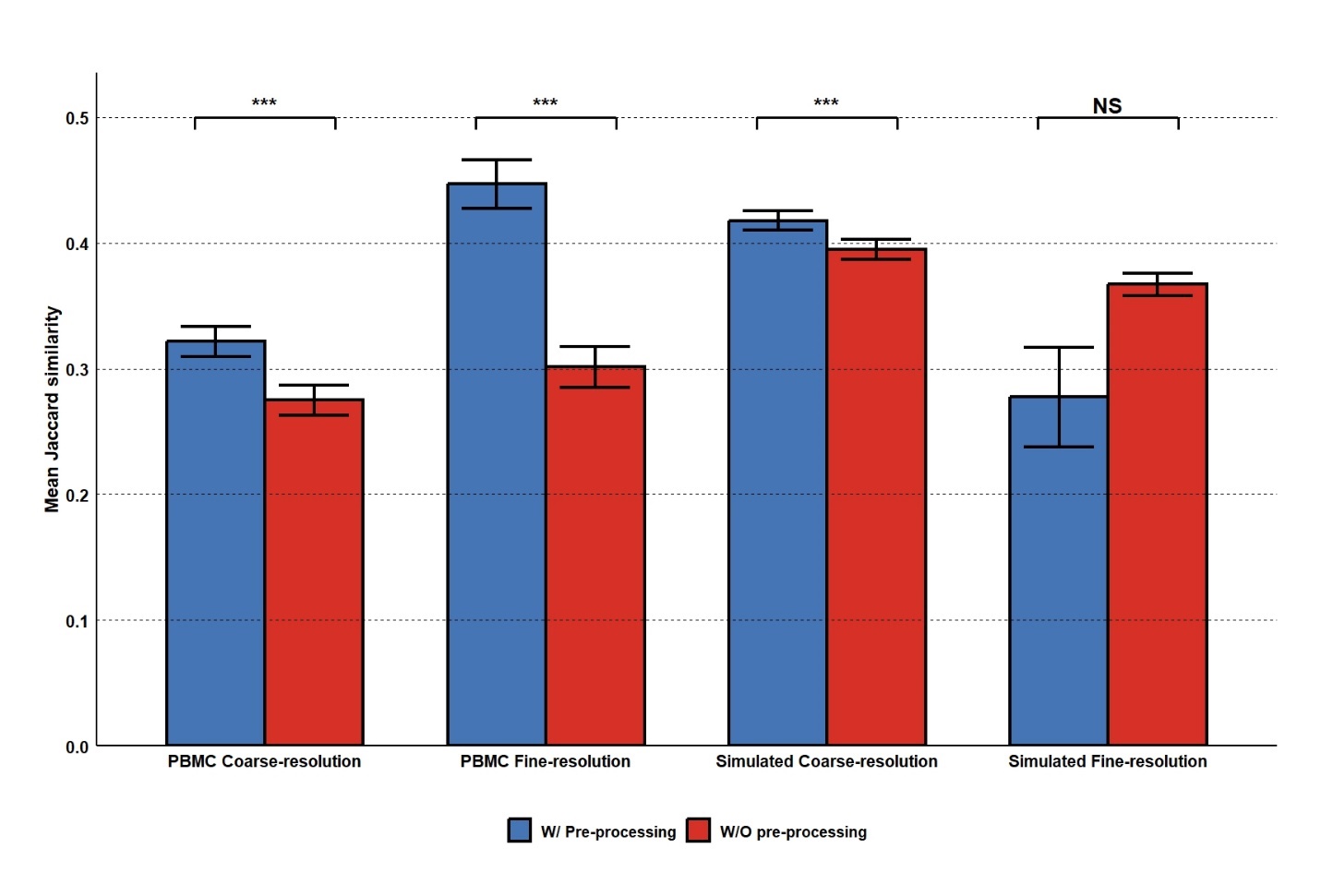
 **Figure S21: Comparison of Mean Jaccard Similarity with and without pre-processing.** x-axis: Data types; y-axis: Mean Jaccard similarity between true HVGs and genes selected by Mcadet. The p-values, obtained through one sided two sample t-test, indicate the significance of the differences between Mcadet method and each other FS method. Ns: non-significance, NS: p $\geq0.05$, *: $0.01\leq$ p < 0.05, **: $0.001\leq$ p < 0.01, ***: p < 0.001,* p < 0.05; **: p < 0.01; ***: p < 0.001.
