# Supplementary material for: Mcadet: A feature selection method for fine-resolution single-cell RNA-seq data based on multiple correspondence analysis and community detection
Source: PLoS Comput Biol. 2024 Oct 28;20(10):e1012560. doi: 10.1371/journal.pcbi.1012560 (PMC11542852; doi:10.1371/journal.pcbi.1012560)
Supplement: S22 Fig — (DOCX) [file pcbi.1012560.s025.docx]

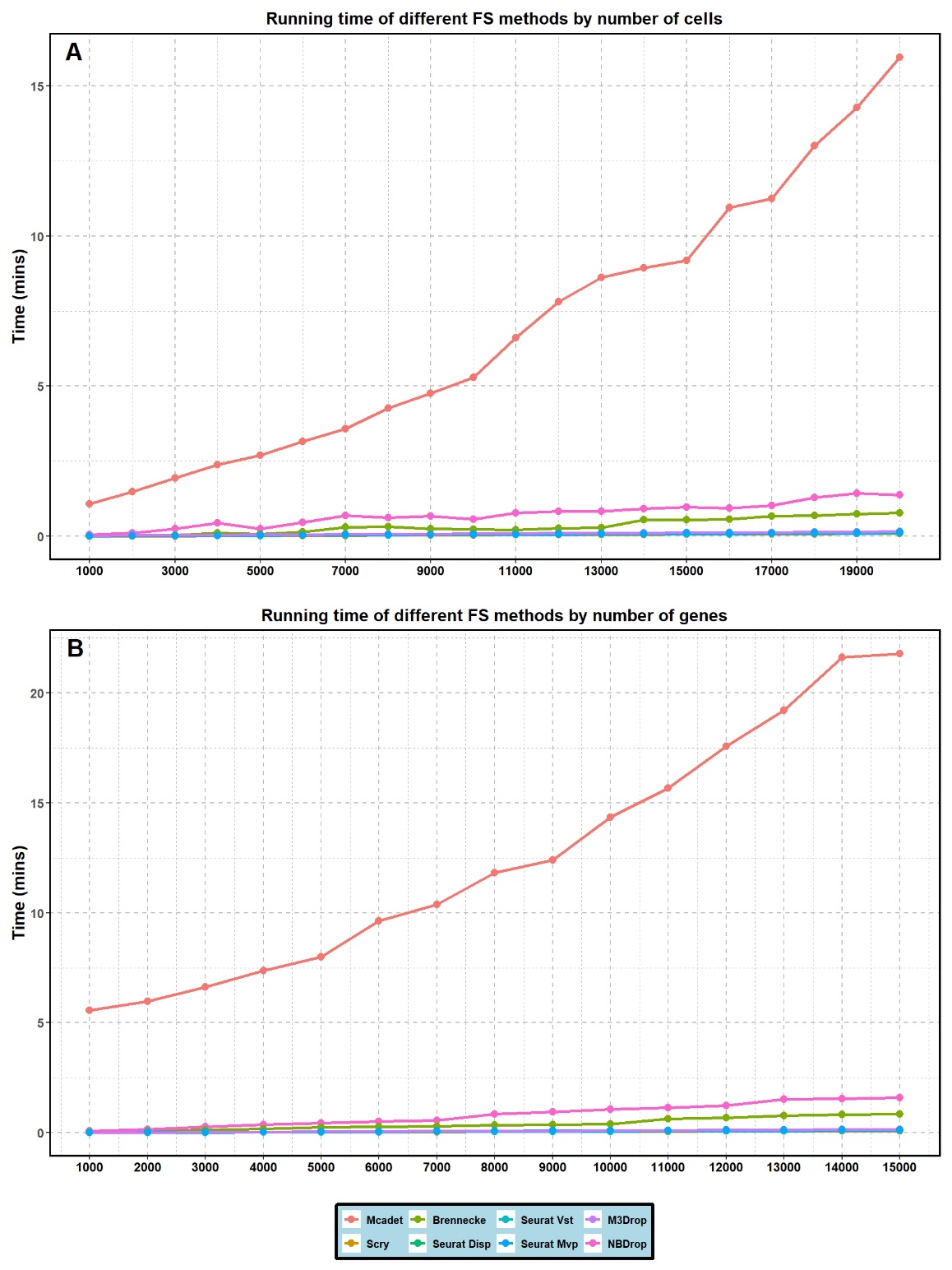


**Figure S22. Running time of different FS methods as number of cells (A) and number of genes (B) increase.** The experiments were conducted using a simulated dataset on a desktop with an AMD Ryzen 9 7950X 16-Core Processor and 64 GB RAM.
