# Supplementary material for: Mcadet: A feature selection method for fine-resolution single-cell RNA-seq data based on multiple correspondence analysis and community detection
Source: PLoS Comput Biol. 2024 Oct 28;20(10):e1012560. doi: 10.1371/journal.pcbi.1012560 (PMC11542852; doi:10.1371/journal.pcbi.1012560)
Supplement: S1 Methods — (DOCX) [file pcbi.1012560.s026.docx]

**B. Methods**

Proof of Eq. (22) and (23) in manuscript:

According to the order statistic and (21), we know that:

$$P\left( X_{\left( d \right)}\leq k \right)=\left( \frac{k}{p} \right)^{d}$$

then,

$$P\left( X_{\left( d \right)}=k \right)=P\left( X_{\left( d \right)}\leq k \right)-P\left( X_{\left( d \right)}\leq k-1 \right)=\left( \frac{k}{p} \right)^{d}-\left( \frac{k-1}{p} \right)^{d}$$

and

$$P\left( X_{\left( 1 \right)}\geq k_{1},X_{\left( d \right)}\leq k_{2} \right)=P\left( k_{1}\leq X_{\left( 1 \right)}\leq k_{2} \right)\cdot P\left( k_{1}\leq X_{\left( 2 \right)}\leq k_{2} \right)\cdots P\left( k_{1}\leq X_{\left( d \right)}\leq k_{2} \right)$$

$$=\left( \frac{k_{2}-k_{1}}{p} \right)^{d}$$

Based on the above formula, we can derive the joint distribution of $X_{\left( 1 \right)}$ and $X_{\left( d \right)}$:

$$P\left( X_{\left( d \right)}\leq k_{2} \right)=P\left( X_{\left( 1 \right)}\leq k_{1},X_{\left( d \right)}\leq k_{2} \right)+P\left( X_{\left( 1 \right)}\geq k_{1},X_{\left( d \right)}\leq k_{2} \right)$$

$$\Rightarrow P\left( X_{\left( 1 \right)}\leq k_{1},X_{\left( d \right)}\leq k_{2} \right)=P\left( X_{\left( d \right)}\leq k_{2} \right)-P\left( X_{\left( 1 \right)}\geq k_{1},X_{\left( d \right)}\leq k_{2} \right)$$

$$=\left( \frac{k_{2}}{p} \right)^{d}-\left( \frac{k_{2}-k_{1}}{p} \right)^{d}$$

then,

$$P\left( X_{\left( 1 \right)}\leq k_{1},X_{\left( d \right)}=k_{2} \right)=P\left( X_{\left( 1 \right)}\leq k_{1},X_{\left( d \right)}\leq k_{2} \right)-P\left( X_{\left( 1 \right)}\leq k_{1},X_{\left( d \right)}\leq k_{2}-1 \right)$$

$$=\left( \frac{k_{2}}{p} \right)^{d}-\left( \frac{k_{2}-k_{1}}{p} \right)^{d}-\left( \frac{k_{2}-1}{p} \right)^{d}+\left( \frac{k_{2}-k_{1}-1}{p} \right)^{d}$$

and,

$$P\left( X_{\left( 1 \right)}=k_{1},X_{\left( d \right)}=k_{2} \right)=P\left( X_{\left( 1 \right)}\leq k_{1},X_{\left( d \right)}=k_{2} \right)-P\left( X_{\left( 1 \right)}\leq k_{1}-1,X_{\left( d \right)}=k_{2} \right)$$

$$=\left( \frac{k_{2}-k_{1}-1}{p} \right)^{d}+\left( \frac{k_{2}-k_{1}+1}{p} \right)^{d}-2\left( \frac{k_{2}-k_{1}}{p} \right)^{d}$$

Therefore, (22) has proved.

Let $R=\frac{X_{\left( d \right)}}{X_{\left( 1 \right)}}$, $U=X_{\left( 1 \right)}\Rightarrow X_{\left( 1 \right)}=U,X_{\left( d \right)}=UR$. Using bivariate transformation to get the joint distribution of $R$ and $U$:

$$P\left( R=r,U=u \right)=P\left( X_{\left( 1 \right)}=U,X_{\left( d \right)}=UR \right)$$

$$=\left( \frac{ur-u-1}{p} \right)^{d}+\left( \frac{ur-u+1}{p} \right)^{d}-2\left( \frac{ur-u}{p} \right)^{d}$$

Then we can get the marginal distribution of $R$: $P\left( R=r \right)=$

$$\sum_{u:\{ur\in Z^{+},ur\leq p\}} P\left( R=r,U=u \right)=\sum_{u:\{ur\in Z^{+},ur\leq p\}} \left[ \left( \frac{ur-u-1}{p} \right)^{d}+\left( \frac{ur-u+1}{p} \right)^{d}-2\left( \frac{ur-u}{p} \right)^{d} \right]$$

Let $V=logR=log\frac{X_{\left( d \right)}}{X_{\left( 1 \right)}}$ as the log ratio between the maximum and the minimum rank. The distribution of $V$ can be derived,

$$P\left( V=v \right)=\sum_{u\in\mathbf{U}} \left[ \left( \frac{ue^{v}-u-1}{p} \right)^{d}+\left( \frac{ue^{v}-u+1}{p} \right)^{d}-2\left( \frac{ue^{v}-u}{p} \right)^{d} \right]$$

where $\mathbf{U}=\{u:ue^{v}\in Z^{+},ue^{v}\leq p\}$.

Therefore, (23) has proved.
